# Enhanced microRNA accumulation and gene silencing efficiency through optimized precursor base pairing

**DOI:** 10.1111/tpj.70665

**Published:** 2026-01-08

**Authors:** Juan‐José Llorens‐Gámez, Pedro José García‐Cano, Sara Rico‐Rodrigo, Lucía Duyos‐Casanova, Sara Toledano‐Franco, Alberto Carbonell

**Affiliations:** ^1^ Instituto de Biología Molecular y Celular de Plantas (Consejo Superior de Investigaciones Científicas–Universitat Politècnica de València) 46022 Valencia Spain

**Keywords:** RNA silencing, artificial microRNA, microRNA precursor, *Nicotiana benthamiana*, Arabidopsis

## Abstract

MicroRNAs (miRNAs) are endogenous 21‐nucleotide small RNAs that direct sequence‐specific silencing of complementary messenger RNAs to regulate a wide range of biological processes. In plants, miRNA precursors are processed from imperfect foldback structures by the RNase III enzyme DICER‐LIKE1, in coordination with accessory proteins. While mismatches flanking the miRNA/miRNA* duplex in endogenous precursors can strongly influence miRNA accumulation, their impact has not been thoroughly examined in the context of artificial miRNAs (amiRNAs) used for targeted gene silencing in plants. Here, using silencing sensor systems in *Nicotiana benthamiana*, we systematically investigated how base pairing at or near DCL1 cleavage sites affects amiRNA production from the recently described minimal *shc* precursor. Independent pairing of naturally mismatched positions revealed that introducing a G–C pair immediately upstream of the mature amiRNA remarkably enhances amiRNA accumulation and silencing efficiency. This effect was further validated in Arabidopsis transgenic lines targeting endogenous genes and confirmed by deep sequencing, which revealed highly accurate processing and predominant release of the intended amiRNAs, supporting the specificity of the approach. Our findings show that a single structural modification in an amiRNA precursor can significantly enhance the efficacy of amiRNA‐mediated gene silencing. This optimized amiRNA platform is well suited for large‐scale functional genomics screens and should facilitate the development of next‐generation crops with enhanced resilience to environmental stresses.

## Significance Statement

A large‐scale mutational analysis revealed that introducing a single G–C base pair upstream of the first DCL1 cleavage site in *shc* precursors enhances artificial microRNA accumulation and silencing efficiency, providing an optimized amiRNA precursor for improved gene regulation and biotechnological applications.

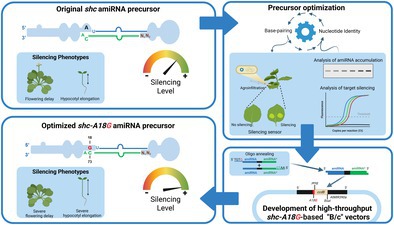

A large‐scale mutational analysis of how base pairing at or near DCL1 cleavage sites affects artificial microRNA (amiRNA) production from the minimal *shc* precursor revealed that introducing a single G–C base pair immediately upstream of the first DCL1 cleavage site significantly boosts amiRNA accumulation and gene silencing efficiency. The incorporation of this structural tweak into a high‐efficiency amiRNA platform provides an optimized, highly specific RNAi tool for functional genomics and crop engineering.

## INTRODUCTION

MicroRNAs (miRNAs) are ∼21‐nucleotide (nt) non‐coding small RNAs (sRNAs) that guide ARGONAUTE (AGO) proteins to complementary messenger RNAs (mRNAs), leading to target cleavage or translational inhibition. In plants, miRNAs regulate genes encoding transcription factors and other proteins involved in critical biological processes including development, stress responses, and hormone signaling (Bologna & Voinnet, 2014; Zhan & Meyers, 2023). They originate from longer precursors transcribed by RNA polymerase II, termed primary miRNAs (pri‐miRNAs), which fold into characteristic stem‐loop foldback structures recognized and sequentially cleaved by the DICER‐LIKE 1 (DCL1) endoribonuclease in the nucleus [reviewed recently in (Yu et al., 2025)]. A miRNA duplex of approximately 21 nt that features 2‐nt 3′ overhangs is excised, and typically one strand—the guide miRNA—is incorporated into an ARGONAUTE (AGO) protein, where it functions to direct gene silencing by base pairing with complementary RNA targets (Carbonell, 2017b; Fang & Qi, 2016).

Plant miRNA precursors are highly diverse in size but share a conserved structural architecture, typically comprising an approximately15–17 bp basal stem (BS), a central miRNA/miRNA* duplex, and a distal stem‐loop (DSL) region that varies in length and conformation and is bordered by single‐stranded regions (Bologna et al., 2009; Cuperus et al., 2011). In the predominant base‐to‐loop processing pathway, the DICER‐LIKE1 (DCL1) enzyme—alongside cofactors SERRATE (SE) and HYPONASTIC LEAVES1 (HYL1)—first cleaves the precursor at the basal stem to generate a shorter hairpin intermediate. A second cleavage, positioned approximately 21 nt from the initial site, releases the miRNA/miRNA* duplex, which possesses characteristic 2‐nt 3′ overhangs and is subsequently stabilized by 2′‐O‐methylation via the methyltransferase HUA ENHANCER1 (HEN1) (Mateos et al., 2010; Song et al., 2010; Werner et al., 2010; Zhu et al., 2013). Alternatively, some miRNA precursors follow an alternative loop‐to‐base processing mode, in which DCL1 initiates cleavage at the terminal loop and proceeds toward the base to excise the miRNA duplex (Bologna et al., 2013; Chorostecki et al., 2017, p. 20178; Addo‐Quaye et al., 2009; Bologna et al., 2009). Despite key structural features required for accurate and efficient miRNA processing having been identified (Bologna et al., 2013; Chorostecki et al., 2017; Cuperus, Montgomery, et al., 2010; Mateos et al., 2010; Moro et al., 2018; Song et al., 2010; Werner et al., 2010; Zhu et al., 2013), the importance of specific sequences in this matter has been largely unknown. Recently, a genome‐wide examination of base pairing interactions at the DCL1 cleavage sites in natural *Arabidopsis thaliana* (Arabidopsis) and eudicot miRNA precursors revealed an enrichment of base pairs among the four positions flanking the miRNA/miRNA* duplex, with sequence biases at specific positions (Rojas et al., 2020). Interestingly, the base pairing of naturally occurring mismatches generally altered miRNA accumulation, with the nucleotide identity and position affecting processing efficiency. Finally, other studies have shown that increasing GC pairing or closing mismatches at miRNA duplexes alters miRNA accumulation and processing efficiency across diverse precursor types, highlighting duplex stability as a tunable determinant of miRNA biogenesis (Narjala et al., 2020; Rosatti et al., 2024).

Artificial miRNAs (amiRNAs) exploit the native plant miRNA biogenesis pathway to induce targeted gene silencing with high specificity, becoming a versatile tool for plant functional genomics and biotechnology (Cisneros et al., 2021). AmiRNAs are engineered 21‐nt sRNAs designed *in silico* to reprogram the endogenous miRNA processing and silencing pathways for specific repression of selected target transcripts with minimal off‐target effects (Carbonell, 2017a; Ossowski et al., 2008; Tiwari et al., 2014). AmiRNAs are typically generated *in planta* by expressing endogenous *MIRNA* precursors in which the native miRNA/miRNA* duplex is replaced with the synthetic amiRNA/amiRNA* duplex, thereby producing a functional pri‐miRNA precursor processed by the endogenous machinery. Choosing an optimal pri‐miRNA backbone is essential to ensure precise and efficient processing of the engineered precursor and accumulate high amiRNA levels required for effective silencing. The 521‐nt long Arabidopsis *MIR390a* (*AtMIR390a*) precursor is processed accurately and efficiently relative to other plant pri‐miRNAs commonly used for amiRNA production (Carbonell et al., 2014; Lunardon et al., 2021), and has been broadly applied for amiRNA expression across various plant species—including both model systems and crops—to achieve effective silencing of endogenous genes and viral RNAs (Berbati et al., 2023; Carbonell et al., 2019; Kadam & Barvkar, 2024; Lunardon et al., 2021; Vasav et al., 2022). Recently, the minimal structural and sequence requirements for producing effective amiRNAs from the *AtMIR390a* precursor were systematically analyzed (Cisneros et al., 2023). As a result, highly effective and accurately processed amiRNAs were produced from a shorted chimeric “*shc*” precursor of only 89 nt, including the complete BS of *AtMIR390a* (without additional ssRNA segments) and the DSL region derived from *Oryza sativa MIR390* with a 2‐nt deletion. Importantly, the *shc* precursor has a compact DSL region of only 15 nt, allowing the synthesis of the entire foldback with just two oligonucleotides. This simple structure has facilitated the development of a cost‐effective, high‐throughput cloning methodology for direct insertion of amiRNA sequences into a suite of “B/c” vectors incorporating the *AtMIR390a* BS, thereby simplifying and accelerating the production of amiRNA constructs (Cisneros et al., 2023).

Here, we used the recently described silencing sensor systems in *Nicotiana benthamiana* (Cisneros et al., 2023; Cisneros & Carbonell, 2025) to systematically investigate how base pairing mismatches at or near DCL1 cleavage sites within the minimal *shc* precursor affect amiRNA biogenesis and function. We functionally screened a large collection of constructs expressing amiRNAs targeting two *N. benthamiana* genes from modified *shc*‐based precursors with distinct base pairing configurations. By combining phenotypic, biochemical, and molecular assays, we show that *shc* precursors in which adenine at position 18 is substituted with a guanine (A18G) yield increased amiRNA levels and markedly enhanced silencing. Finally, the superior performance of A18G‐modified *shc* precursors was further validated in Arabidopsis transgenic plants expressing amiRNAs against endogenous genes whose silencing induced a visible and quantifiable phenotype. Furthermore, high‐throughput sequencing‐based analysis of precursor processing showed that A18G‐modified *shc* precursors are accurately processed and release authentic amiRNAs.

## RESULTS

### Enhanced accumulation of miR390a from a modified *AtMIR390a* precursor without mismatches at DCL1 first cleavage site

**Figure 1:**
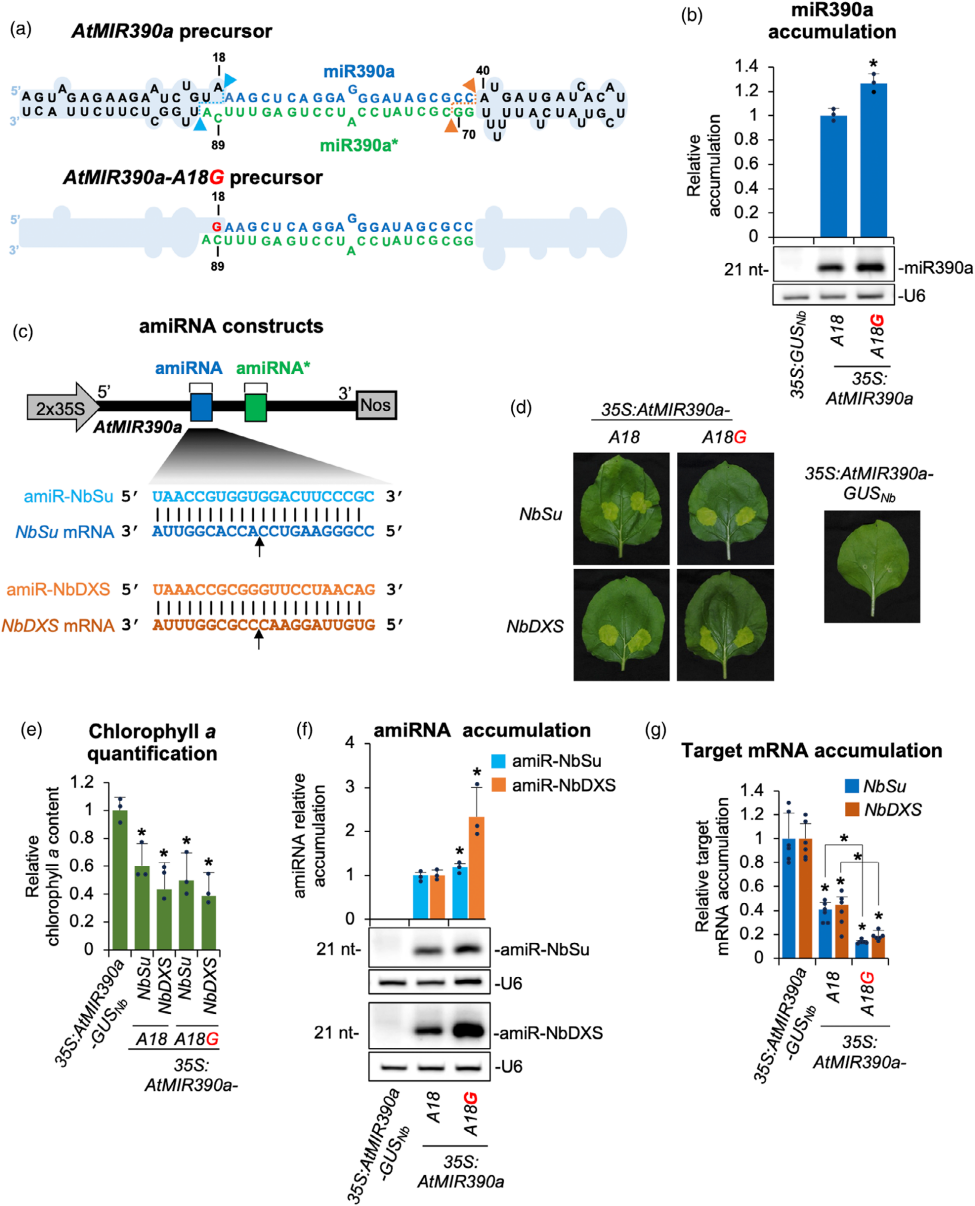
Functional analysis of endogenous and modified Arabidopsis *MIR390a* (AtMIR390a)‐based precursors without mismatches at DCL1 first cleavage site. (a) *AtMIR390a* and *AtMIR390a‐A18G* foldback diagrams. DCL1 first and second cleavage sites are marked with blue and orange arrows, respectively. *AtMIR390a*, miR390a and miR390a* nucleotides are highlighted in black, blue, and green, respectively. Mutated G at position 18 is shown in red. Shapes corresponding to *AtMIR390a* basal stem and distal stem‐loop are in light blue. (b) Northern blot detection of miR390a in RNA preparations from agroinfiltrated leaves at 2 days post‐agroinfiltration (2 dpa). The graph at top shows the mean (*n* = 3) + standard deviation miR390a relative accumulation (*35S:AtMIR390a* = 1). Bar with the asterisk “*” is significantly different from that of the *35S:AtMIR390a* control sample (*P* < 0.05 in pairwise Student's *t*‐test comparison). A representative blot from three biological replicates is shown (c) Diagram of *AtMIR390a*‐based amiRNA constructs including the base pairing of amiRNAs and target mRNAs. Nucleotides corresponding to the guide strand of the amiRNA against *NbSu* and *NbDXS* are in blue and orange, respectively, while nucleotides of target mRNAs are in dark blue and orange, respectively. The arrows indicate the amiRNA‐predicted cleavage site. (d) Photos at 7 dpa of leaves agroinfiltrated with different constructs. (e) Bar graph showing the relative content of chlorophyll *a* in patches agroinfiltrated with different constructs (*35S:AtMIR390a‐GUS* = 1.0). Bars with an asterisk “*” are significantly different from that of the *35S:AtMIR390a‐GUS* control sample (*P* < 0.05 in pairwise Student's *t*‐test comparisons). (f) Northern blot detection of amiR‐NbSu and amiR‐NbDXS in RNA preparations from agroinfiltrated leaves at 2 dpa. Other details are as in (b). (g) Target mRNA accumulation in agroinfiltrated leaves. Mean relative level (*n* = 3) + standard error of *NbSu* or *NbDXS* mRNAs after normalization to *PROTEIN PHOSPHATASE 2A* (*PP2A*), as determined by quantitative RT‐PCR (qPCR) (*35S:AtMIR390a‐GUS*_*Nb*_ = 1.0 in all comparisons). Bars with the asterisk are significantly different from that of the control sample *35S:AtMIR390a‐GUS*_*Nb*_ (*P* < 0.05 in pairwise Student's *t*‐test comparisons). Additional significant pairwise comparisons are also indicated with an asterisk.

To assess the impact of eliminating mismatches at the DCL1 initial cleavage site on miR390a biogenesis, we engineered a modified Arabidopsis *MIR390a* precursor in which the adenine at position 18 was substituted with guanine (A18G), thus restoring base pairing with cytosine at position 89 (C89) (Figure 1a). This modified construct (*35S:AtMIR390a‐A18G*) and a wild‐type control (*35S:AtMIR390a*) were transiently expressed in *N. benthamiana* leaves through *Agrobacterium* infiltration. Each construct was agroinfiltrated into two leaves per plant across three biological replicates. A *35S:GUS* construct expressing *Escherichia coli* β‐glucuronidase *uidA* gene served as a negative control. sRNA blot analysis at 2 days post‐agroinfiltration (dpa) revealed a significant 26.5% increase in miR390a accumulation from the mismatch‐corrected precursor compared with the wild‐type (Figure 1b).

### Increased amiRNA accumulation and activity from the modified *AtMIR390a‐A18G* precursor

To determine whether the *AtMIR390a‐A18G* precursor could enhance accumulation of artificial miRNAs (amiRNAs), we used two previously described gene silencing reporters targeting the endogenous *N. benthamiana SULFUR* (*NbSu*) and *DXS* (*NbDXS*) genes, which encode magnesium chelatase subunit CHLI and 1‐deoxy‐D‐xylulose‐5‐phosphate synthase, respectively (Cisneros et al., 2023). Visible bleaching phenotypes indicate efficient silencing of these targets. The use of two distinct amiRNA sequences, amiR‐NbSu and amiR‐NbDXS, allowed us to specifically evaluate the impact of precursor base pairing independently of the primary amiRNA sequence expressed. Constructs expressing amiR‐NbSu and amiR‐NbDXS from the modified precursor were generated (Figure 1c) and agroinfiltrated into two regions per leaf of two leaves across three plants. Parallel infiltrations with constructs expressing amiR‐NbSu, amiR‐NbDXS, and amiR‐GUS (an amiRNA targeting *E. coli* β *uidA* gene) (Cisneros et al., 2022) from the wild‐type *AtMIR390a* precursor were also performed as controls. At 7 dpa, leaf sectors expressing amiR‐NbSu or amiR‐NbDXS displayed strong bleaching phenotypes (Figure 1d), correlating with significant reductions in chlorophyll *a* content compared with the control (Figure 1e). Next, two leaves of three different plants were independently agroinfiltrated in the whole leaf surface with each of the amiRNA constructs described above. RNA blot analysis at 2 dpa of leaves fully infiltrated with each amiRNA construct confirmed that amiR‐NbSu and amiR‐NbDXS accumulated at higher levels when expressed from the A18G‐modified precursor (Figure 1f), showing 19 and 133% increases, respectively, compared with the wild‐type precursor. Consistently, RT‐qPCR analysis revealed significantly reduced *NbSu* and *NbDXS* transcript levels in samples expressing the corresponding amiRNAs from the modified precursor (Figure 1g). Specifically, *NbSu* and *NbDXS* mRNA levels decreased to 40.7 and 44.9%, respectively, of the amiR‐GUS control when expressed from the wild‐type precursor, and to 13.3 and 18.9%, respectively, when expressed from the A18G‐modified precursor, confirming the enhanced silencing efficiency conferred by the A18G modification.

### Analysis of amiRNA accumulation in modified *sch* precursors without mismatches at DCL1 first cleavage site

**Figure 2:**
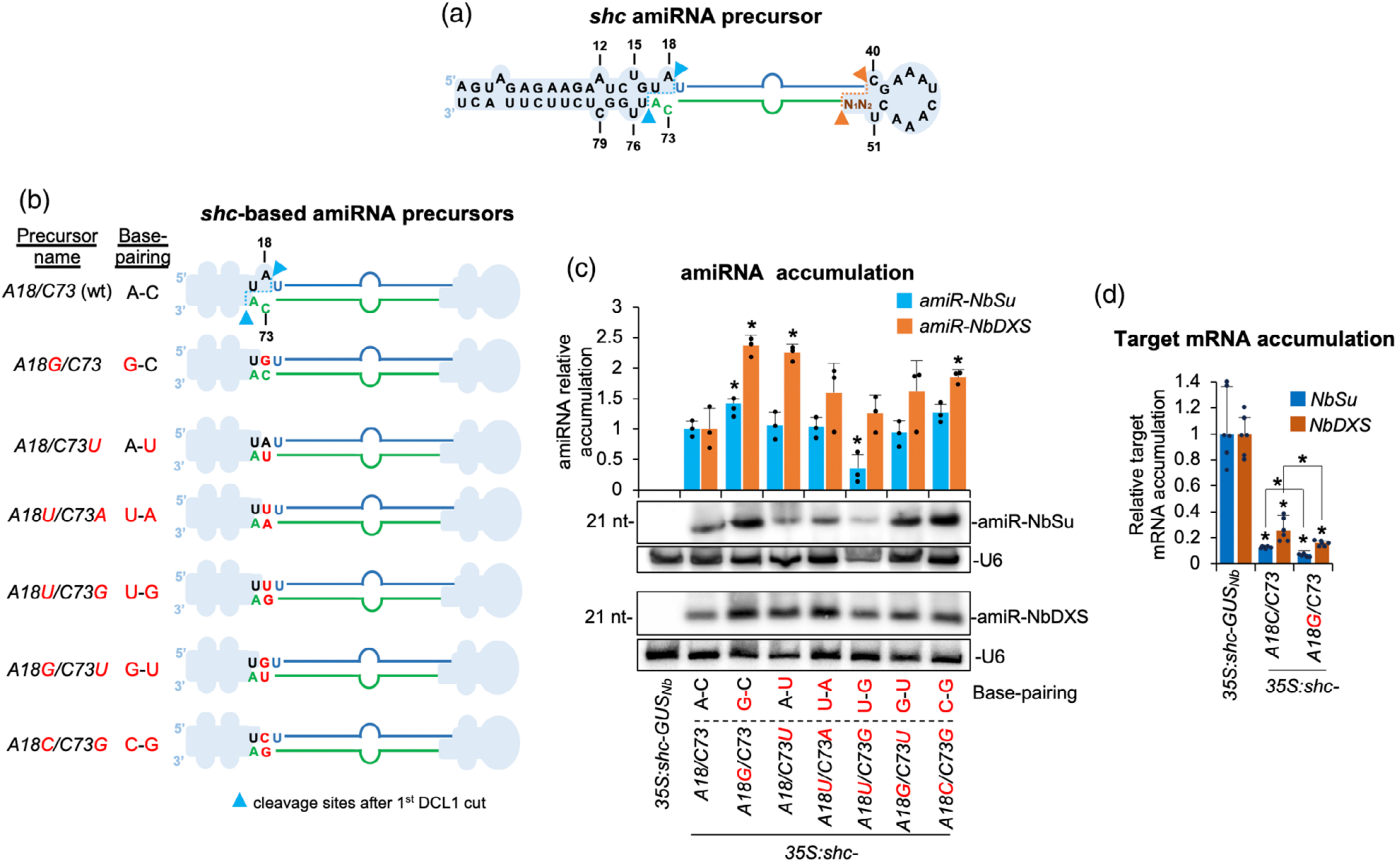
Functional analysis of wild‐type and modified *shc‐*based precursors without mismatches at DCL1 first cleavage site. (a) *shc* foldback diagrams with DCL1 first and second cleavage sites are marked with blue and orange arrows, respectively. miR390a and miR390a* nucleotides are highlighted in blue and green, respectively. Relevant unpaired positions are numbered. (b) Diagrams of the amiRNA precursors with mutated nucleotides at position 18 in red. Nucleotides of the precursor, amiRNA and amiRNA* are in black, blue and green, respectively. The shapes corresponding to *shc* basal stem or distal stem‐loop are in light blue. (c) Northern blot detection of amiR‐NbSu and amiR‐NbDXS in RNA preparations from agroinfiltrated leaves at 2 dpa. Bars with an asterisk “*” are significantly different from that of the corresponding wild‐type *shc‐NbSu/shc‐NbDXS* control samples (*P* < 0.05 in all pairwise Student's *t*‐test comparisons). A representative blot from three biological replicates is shown. (d) Target mRNA accumulation in agroinfiltrated leaves. Mean relative level (*n* = 3) + standard error of *NbSu* or *NbDXS* mRNAs after normalization to *PROTEIN PHOSPHATASE 2A* (*PP2A*), as determined by quantitative RT‐PCR (qPCR) (*35S:shc‐GUS*_*Nb*_ = 1.0 in all comparisons). Bars with an asterisk “*” are significantly different from that of the control sample *35S:shc‐GUS*_*Nb*_ (*P* < 0.05 in all pairwise Student's *t*‐test comparisons). Additional significant pairwise comparisons are also indicated with an asterisk.

To further investigate whether the base pairing at DCL1 first cleavage site enhances amiRNA accumulation from other precursors, we analyzed the recently described *shc* amiRNA precursor (Figure 2a) (Cisneros et al., 2023). Mutations were introduced at positions 18 and 73 of the basal stem to assess the effect on amiRNA accumulation of nucleotide identity and specific base pair combinations at the DCL1 first cleavage site. Constructs expressing amiR‐NbSu and amiR‐NbDXS from *shc‐*based variant precursors including all possible paired nucleotide combinations at the DCL1 first cleavage site (positions 18/73) were generated (Figure 2b). These constructs, along with the GUS‐targeting control, were transiently expressed in *N. benthamiana* leaves through agroinfiltration, as previously described.

sRNA blot analyses of RNA samples from agroinfiltrated leaves collected at 2 dpa revealed that all precursor variants produced detectable levels of mature amiRNAs (Figure 2c). Regarding amiR‐NbSu, accumulation was significantly higher in samples expressing the A18G/C73 variant relative to the wild‐type configuration, showing a 42.3% increase in amiRNA abundance (Figure 2c). In the case of amiR‐NbDXS, all modified precursors exhibited enhanced accumulation, although only those with A18G, C73U, or A18G/C73G modifications showed statistically significant increases, displaying 137, 126, and 85% higher amiRNA levels, respectively, compared with the wild‐type precursor (Figure 2c). Finally, target transcript levels were analyzed in tissues expressing amiR‐NbSu or amiR‐NbDXS from the A18G/C73 precursor, which produced the highest (and significant) accumulation of both amiRNAs. RT‐qPCR analysis revealed a significant reduction in *NbSu* and *NbDXS* transcript abundance in these samples (Figure 2d). Specifically, *NbSu* and *NbDXS* mRNA levels decreased to 12.1 and 25.7%, respectively, of the amiR‐GUS control when expressed from the wild‐type precursor, and to 7.7 and 16.2%, respectively, when expressed from the A18G‐modified precursor, therefore confirming the enhanced silencing efficiency conferred by this modified precursor.

### Analysis of amiRNA accumulation in modified *sch* precursors without mismatches at DCL1 second cleavage site or at other basal stem positions

**Figure 3:**
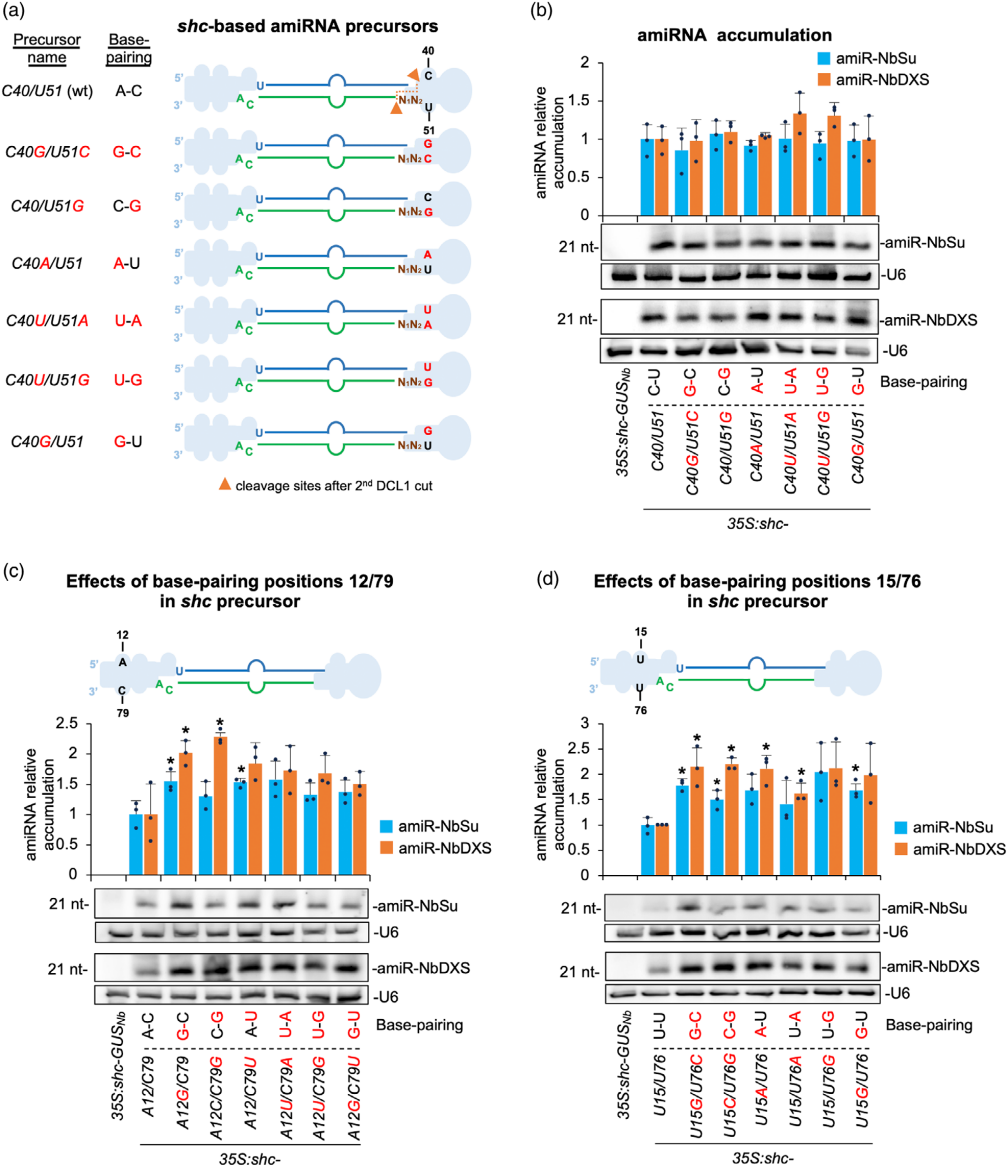
Functional analysis of wild‐type and modified *shc‐*based precursors without mismatches at DCL1 second cleavage site and at positions 12/79 and 15/76. (a) Diagrams of the amiRNA precursors with mutated nucleotides at position 40/51 (corresponding to DCL1 second cleavage site) in red. Other details are as in Figure 2b. (b) Northern blot detection of amiRNAs. Bars with an asterisk “*” are significantly different from that of the corresponding wild‐type shc‐NbSu/shc‐NbDXS control samples (*P* < 0.05 in all pairwise Student's *t*‐test comparisons). A representative blot from three biological replicates is shown. (c) Top, diagram of wild‐type *shc* amiRNA precursor with unpaired position 12/79 highlighted. Other details are as in Figure 2b. Bottom, Northern blot detection of amiRNAs. Other details are as in (b). (d) Top, diagram of wild‐type *shc* amiRNA precursor with unpaired position 15/76 highlighted. Other details are as in Figure 2b. Bottom, Northern blot detection of amiRNAs. Other details are as in (b).

Next, we sought to determine how nucleotide identity and base pairing affect amiRNA accumulation at the DCL1 second cleavage site. For that purpose, we further modified the *shc* precursor at positions 40 and 51, which define the second DCL1 cleavage site (Figure 3a). A series of *shc‐*based precursors containing all possible nucleotide combinations at positions 40/51 were generated (Figure 3a), each expressing amiR‐NbSu or amiR‐NbDXS. These constructs, along with the GUS‐targeting control, were transiently expressed in *N. benthamiana* leaves through agroinfiltration, as previously described. sRNA blot analyses of RNA samples from agroinfiltrated leaves collected at 2 dpa revealed detectable levels of amiR‐NbSu and amiR‐NbDXS from all modified precursor variants (Figure 3b), with no significant differences among the different variants, indicating that base pairing at the DCL1 second cleavage site in *shc* has not a significant effect on amiRNA accumulation.

To further dissect how internal stem base pairing influences amiRNA processing, we independently altered nucleotide identity and pairing at positions 12/79 and 15/76, two sites proximal to the DCL1 first cleavage site within the basal stem of the *shc* precursor and assessed their impact on amiRNA accumulation. For the 12/79 position, sRNA blot analysis showed higher accumulation of both amiRNAs from all modified precursor variants compared with wild‐type configuration (Figure 3c). Notably, amiR‐NbSu accumulation was significantly higher (*P* < 0.05, *t*‐test) in samples expressing the single‐mutant A12G or C79U precursor variants only relative to the wild‐type precursor (54 and 53% increases, respectively), while amiR‐NbDXS levels were significantly higher (*P* < 0.05, *t*‐test) for the single‐mutant A12G or C79G variants (102 and 129% increases, respectively) (Figure 3c). Similarly, at position 15/76, amiRNA accumulation was generally higher across all variants, with the U15G/U76C‐U15C/U76G‐U15G/U76 and U15G/U76C‐U15C/U76G‐U15A/U76‐U15/U76A variants producing significantly higher amounts of amiR‐NbSu and amiR‐NbDXS, respectively (Figure 3d). Together, these results indicate that base pairing at internal positions 12/79 and 15/76 from *shc* basal stem generally increases amiRNA accumulation.

### Combined effects of multiple base pairing modifications on amiRNA accumulation in *shc* precursors

**Figure 4:**
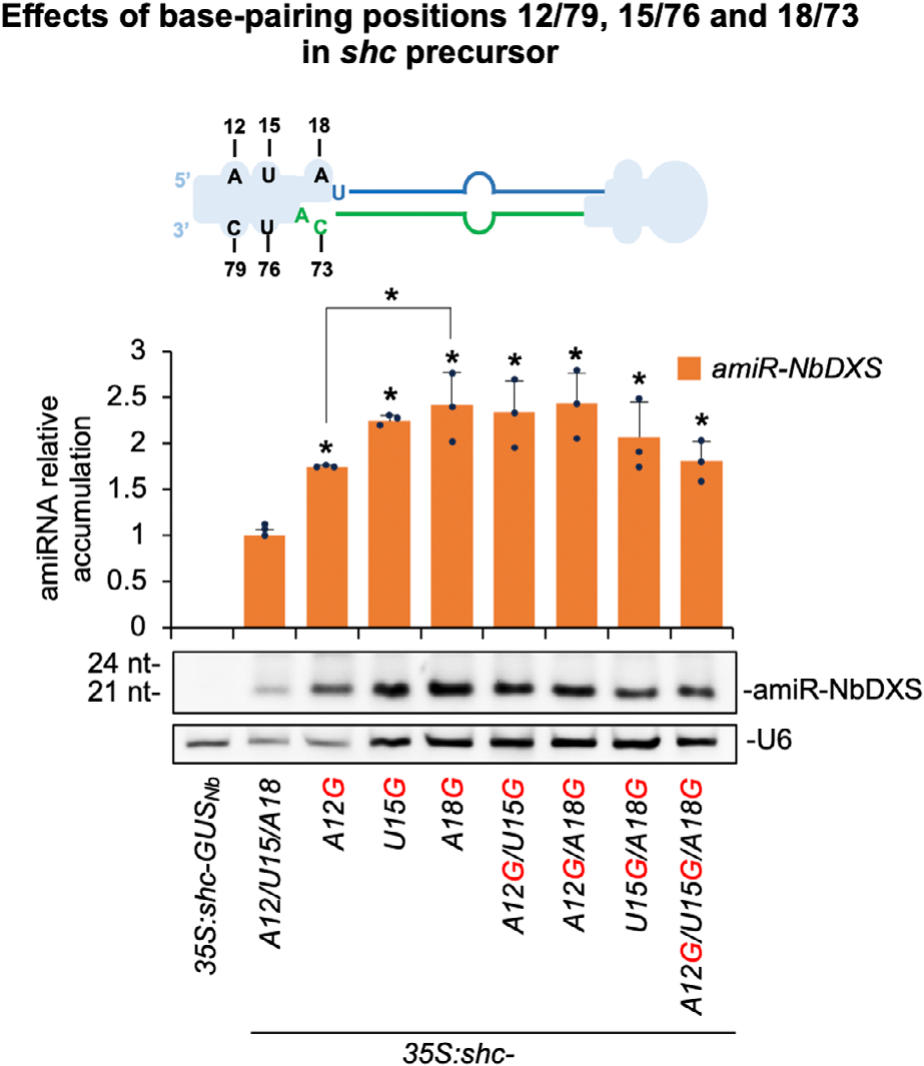
Functional analysis of wild‐type and modified *shc‐*based precursors without mismatches at positions 12/79, 15/76 and 18/73 in single, double, and triple combinations. Top, diagram of wild‐type *shc* amiRNA precursor with unpaired positions 12/79, 15/76 and 18/73 highlighted. Other details are as in Figure 2b. Bottom, Northern blot detection of amiR‐NbDXS. Bars with an asterisk “*” are significantly different from that of the corresponding wild‐type *35S:shc‐NbDXS or 35S:shc‐A18G‐NbDXS* samples, respectively (*P* < 0.05 in all pairwise Student's *t*‐test comparisons). Additional significant pairwise comparisons are also indicated with an asterisk. Other details are as in Figure 2c.

To assess the cumulative impact of introducing multiple base pair changes within the basal stem of the *shc* precursor, we generated double and triple mutants targeting positions 12/79, 15/76, and 18/73, regions located in close proximity to the DCL1 first cleavage site (Figure 4), and compared amiRNA accumulation from these variants to that observed in the corresponding single mutants. These modifications were selected based on previous analyses showing that single substitutions A12G, U15G, and A18G significantly enhanced amiRNA accumulation (Figures 2, 3, 4). Given the comparable responses observed for amiR‐NbSu and amiR‐NbDXS in earlier experiments, only amiR‐NbDXS‐expressing constructs were used in this analysis for simplicity. All constructs were transiently expressed in *N. benthamiana* leaves, and amiRNA accumulation was analyzed at 2 dpa using sRNA blot assays.

All modified precursors produced significantly higher levels of amiR‐NbDXS relative to the wild‐type control (Figure 4). Among the single mutants, A18G supported the highest amiRNA accumulation, showing a 140% increase relative to the wild‐type; however, this increase was not significantly different from that observed with the U15G variant, while it was significantly higher than that conferred by the A12G variant, which showed reduced accumulation (Figure 4). Importantly, neither the double (A12G/U15G, A12G/A18G, U15G/A18G) nor the triple (A12G/U15G/A18G) mutants conferred further enhancement relative to the A18G single mutant, indicating a lack of additive or synergistic effects in this context. Based on these results, the *shc‐A18G* variant, which reproducibly supports robust amiRNA accumulation, was selected for subsequent analyses.

### Processing accuracy of *shc* and *shc‐A18G* precursors releasing amiR‐NbDXS

**Figure 5:**
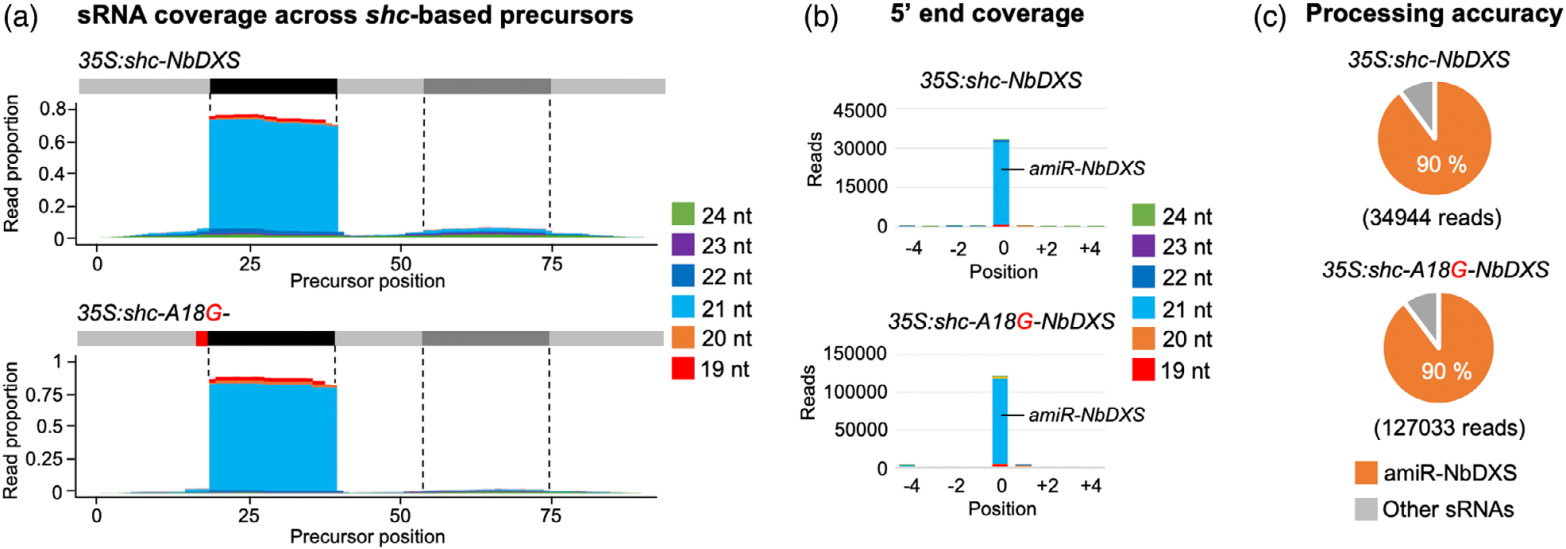
Processing of amiR‐NbDXS from wild‐type and A18G‐modified *shc* precursors. (a) Small RNA (sRNA) coverage across *shc*‐based precursors. The *x*‐axis indicates the position on the precursor in nucleotides, from 5′ to 3′. At the top of each plot, the light gray line corresponds to the precursor backbone; the position of the amiRNA and amiRNA* in the precursors are indicated in black and dark gray respectively, and the A18G substitution in red. The *y*‐axis is the sRNA coverage in proportion of reads for each nucleotide position aligning to the positive strand. Coverage of reads of different lengths is shown in separate colors, stacked from bottom to top as indicated in the legend on the right. (b) sRNA 5′ coverage around the artificial miRNA (amiRNA) 5′ end in *shc*‐based precursors. In the *x*‐axis, 0 indicates the 5′ end of the amiRNA, −4 and + 4 indicate 4 nt upstream and downstream of them. They *y*‐axis is the sRNA 5′ coverage in total reads. The light blue portion of the bar at 0 represents authentic amiR‐NbDXS reads. Other details are as in (a). (c) amiRNA processing accuracy from *shc*‐based precursors. Pie charts show percentages of reads corresponding to expected, accurately processed 21‐nt mature amiR‐NbDXS (orange sectors) or to other 19‐24‐nt sRNAs (gray sectors).

To further confirm processing accuracy of *shc‐A18G* precursors, sRNA libraries were prepared from plants expressing *35S:shc‐A18G‐NbDXS* and sequenced. For comparison, sRNA datasets from *35S:shc‐NbDXS* samples were also analyzed (Cisneros et al., 2023). In both precursors, read coverage concentrated almost exclusively within the predicted amiRNA/amiRNA* region, with negligible accumulation along the remaining backbone (Figure 5a). The size profile was strongly dominated by 21‐nt reads corresponding to the expected amiR‐NbDXS sequence, while other sRNA species contributed only marginally. Moreover, reads were aligned relative to the amiR‐NbDXS 5′ terminus; the high precision of processing became evident (Figure 5b). In both precursors, the vast majority of 21‐nt reads, corresponding to authentic amiR‐NbDXS, initiated exactly at position 0, with only minor offset reads detected at −1 or +1. The position‐0 signal greatly exceeded neighboring positions for both precursors, reflecting the high 5′‐end fidelity of the mature guide strand. Finally, quantification of processing accuracy, defined as the proportion of reads that perfectly matched the expected 21‐nt amiR‐NbDXS within the −4/+4 region surrounding the 5′ end, revealed a 90% accuracy for both precursors (Figure 5c). Collectively, these results show that the *shc‐A18G* variant enhances amiR‐NbDXS accumulation while fully preserving the defining features of accurate DCL1 processing: dominant 21‐nt production, confinement of reads to the guide strand region, and a sharp, precisely defined 5′ end.

### New high‐throughput vectors for expressing amiRNAs from *shc‐A18G*‐based precursors

To facilitate high‐throughput cloning and expression of amiRNAs from *shc‐A18G* precursors, we developed two new “B/c” vectors incorporating the A18G‐modified basal stem of *AtMIR390a* (Figure S1): (i) *pENTR‐BS‐AtMIR390a‐A18G‐B/c*, a Gateway‐compatible entry vector enabling direct insertion of amiRNA sequences and subsequent recombination into preferred expression vectors with customizable promoters, terminators, and regulatory features; and (ii) *pMDC32B‐BS‐AtMIR390a‐A18G‐B/c*, a binary vector suitable for direct *Agrobacterium*‐mediated transformation, eliminating intermediate subcloning steps (Figure S2). Both vectors contain the truncated *BS‐AtMIR390a‐A18G* region followed by a 1461‐bp DNA cassette encoding the *ccd*B negative selection marker (Bernard & Couturier, 1992), flanked by two inverted *Bsa*I restriction sites positioned downstream of the precursor sequence. amiRNA constructs are generated using an established and cost‐effective B/c cloning strategy (Carbonell et al., 2014; Cisneros et al., 2023). Briefly, amiRNA inserts are prepared by annealing two 58‐nt overlapping and partially complementary oligonucleotides carrying the amiRNA sequence, with 5′‐TGTG and 5′‐AATG overhangs, and directionally ligated into *Bsa*I‐digested *BS‐AtMIR390a‐A18G‐B/c* vectors (Figure S2 and Text S1). These vectors were subsequently used throughout this study for functional validation of amiRNAs expressed from optimized *shc‐A18G* precursors.

### Enhanced target silencing in transgenic Arabidopsis expressing A18G‐modified *shc* amiRNA precursors

**Figure 6:**
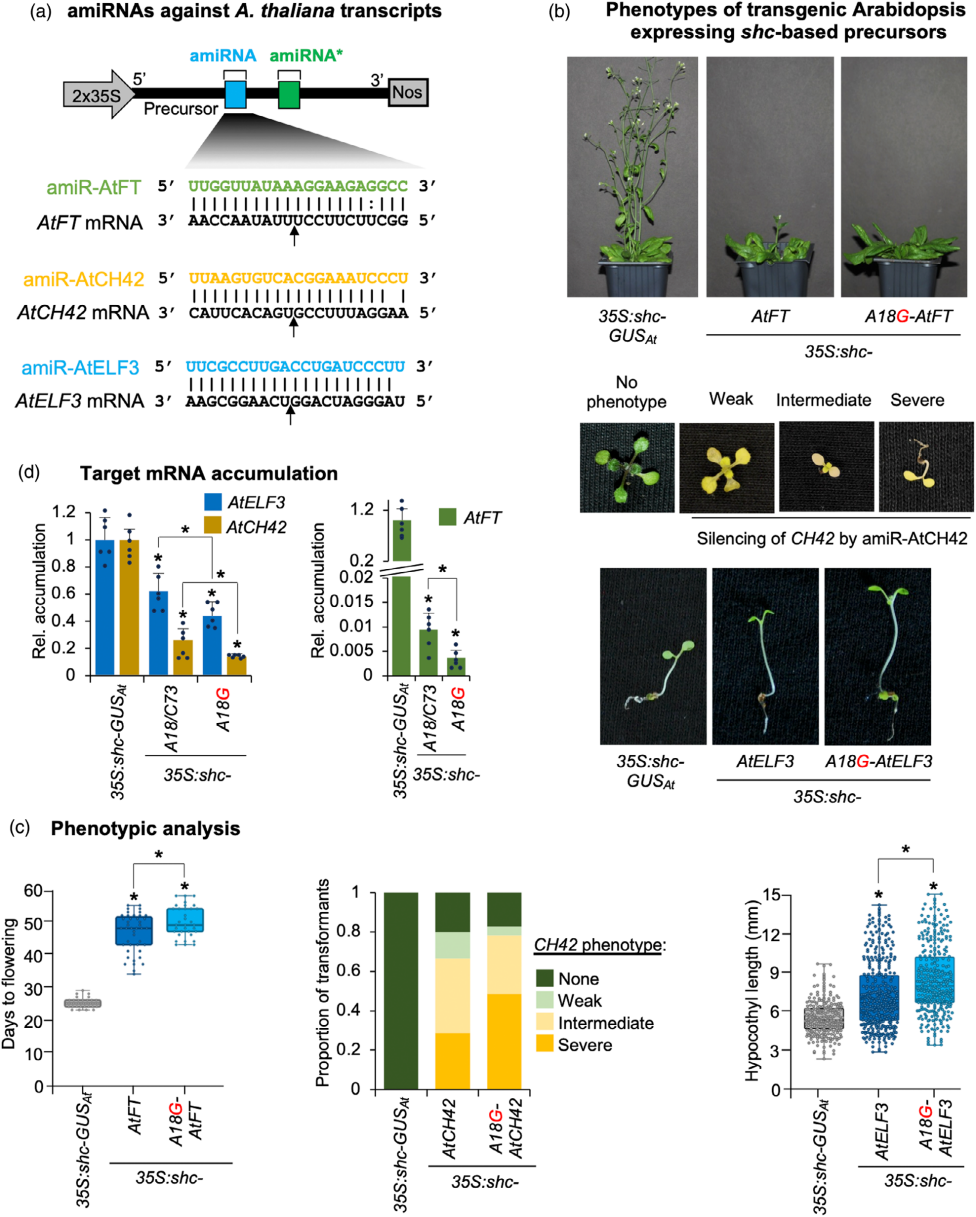
Functional analysis of constructs expressing the amiR‐AtFT, amiR‐AtCH42, and amiR‐AtELF3 amiRNAs against Arabidopis *FLOWERING LOCUS T* (*AtFT*), *CHLORINE 42* (*AtCH42*), or *EARLY FLOWERING 3* (*AtELF3*) from wild‐type and A18G‐modified *shc* precursors. (a) Diagram of *shc*‐based amiRNA constructs including the base pairing of amiRNAs and target mRNAs. Nucleotides corresponding to the guide strand of the amiRNA against *AtFT*, *AtCH42*, and *AtELF3* are in green, yellow, and blue, respectively, while nucleotides of target mRNAs are in black. The arrows indicate the amiRNA‐predicted cleavage site. (b) Representative images of Arabidopsis T1 transgenic plants expressing amiRNAs from different precursors. Top, 45‐day‐old adult plants expressing amiR‐GUS_At_ or amiR‐AtFT. Middle, 10‐day‐old T1 seedlings expressing amiR‐AtCH42 and showing bleaching phenotypes of diverse degrees. Bottom, 10‐day‐old T1 seedlings expressing amiR‐AtELF3 and showing elongated hypocotyls. (c) Phenotyping analysis. Left, box plot representing the mean flowering time of Arabidopsis T1 transgenic plants expressing amiR‐GUS_At_ or amiR‐AtFT from different precursors. Center, bar graph representing, for each line, the proportion of seedlings displaying a severe (black areas), intermediate (dark gray areas), or weak (light gray areas) bleaching phenotype, or with wild‐type appearance (white areas). Right, box plot representing the mean hypocotyl length of Arabidopsis T1 transgenic plants expressing amiR‐GUS_At_ or amiR‐AtELF3 from different precursors. Pairwise Student's *t*‐test comparisons are represented with an asterisk “*” if significantly different from *35S:Shc‐GUS*_*At*_ samples (*P* < 0.05). Additional significant pairwise comparisons are also indicated with an asterisk. A representative blot from three biological replicates is shown. (d) Target *AtFT*, *AtCH42*, and *AtELF3* mRNA accumulation in RNA preparations from Arabidopsis plants [mean relative level (*n* = 3) + standard error] after normalization to Actin 2, as determined by quantitative RT‐qPCR (*35S:Shc‐GUS*_*At*_ = 1). Bars with an asterisk “*” are significantly different from *35S:Shc‐GUS*_*At*_ samples (*P* < 0.05). Additional significant pairwise comparisons are also indicated with an asterisk. Each biological replicate is a pool of at least nine independent lines selected randomly.

To evaluate the performance of the A18G‐modified *shc* precursor in a stable genetic context, we generated transgenic Arabidopsis plants expressing amiRNAs amiR‐AtFT, amiR‐AtELF3, and amiR‐AtCH42 (Figure 6a) targeting endogenous *FLOWERING LOCUS T* (*AtFT*), *EARLY FLOWERING 3* (*AtELF3*), or *CHLORINA 42* (*AtCH42*) endogenous genes, respectively, from either the wild‐type or A18G‐modified *shc* precursors. Efficient silencing of *AtFT*, *AtELF3*, and *AtCH42* should result in a significant delay in flowering time, hypocotyl elongation, or intense bleaching, respectively, as described before (Kim & Somers, 2010; Schwab et al., 2006). Briefly, we introduced amiR‐AtFT, amiR‐AtELF3, and amiR‐AtCH42 into *pMDC32B‐BS‐AtMIR390a‐A18G‐B/c* to generate the *35S:shc‐A18G‐AtFT*, *35S:shc‐A18G‐AtELF3*, and *35S:shc‐A18G‐AtCH42* constructs, respectively. These constructs were independently transformed into Arabidopsis Col‐0 plants, along with control constructs *35S:shc‐AtFT*, *35S:shc‐AtELF3*, *35S:shc‐AtCH42*, and *35S:shc‐GUS*_*At*_, which express an amiRNA targeting *GUS* (with no predicted off‐targets in Arabidopsis) from the *shc* precursor (Cisneros et al., 2023). To systematically compare the processing and silencing efficacy of amiRNAs produced from wild‐type versus A18G precursors, we analyzed plant phenotypes, amiRNA accumulation, target mRNA levels, and processing accuracy in Arabidopsis T1 transgenic lines.

Phenotypic analyses revealed that all *35S:shc‐A18G‐AtFT* (*n* = 34) transgenic lines expressing amiR‐AtFT from the A18G‐modified *shc* precursor exhibited a significantly delayed flowering time relative to those expressing the same amiRNA from the wild‐type *shc* precursor (*35S:shc‐AtFT*, *n* = 43), with mean flowering times of 46.6 ± 6 and 50 ± 4.7 days, respectively (Figure 6b,c, left; Table S1). Similarly, *35S:shc‐A18G‐AtCH42* seedlings expressing amiR‐AtCH42 from the A18G‐modified precursor displayed stronger bleaching phenotypes, with a higher proportion of individuals (48.7%) exhibiting severe chlorosis compared with those transformed with wild‐type *shc 35S:shc‐AtCH42* (28.8%) (Figure 6b,c, right; Table S1). In the case of amiR‐AtELF3, phenotypic evaluation based on hypocotyl length under short‐day conditions revealed that *35S:shc‐A18G‐AtELF3* transformants showed, on average, significantly enhanced hypocotyl elongation relative to *35S:shc‐AtELF3* lines (Figure 6b,c, bottom; Table S1). These enhanced phenotypic effects were consistent with a stronger repression of their respective targets (Figure 6d) in lines expressing the A18G‐modified precursor. Specifically, *AtFT*, *AtCH42*, and *AtELF3* mRNA levels decreased to 0.94, 26.2, and 14.1%, respectively, of the amiR‐GUS control when expressed from the wild‐type precursor, and to 0.37, 14.1, and 44.1%, respectively, when expressed from the A18G‐modified precursor (Figure 6d).

**Figure 7:**
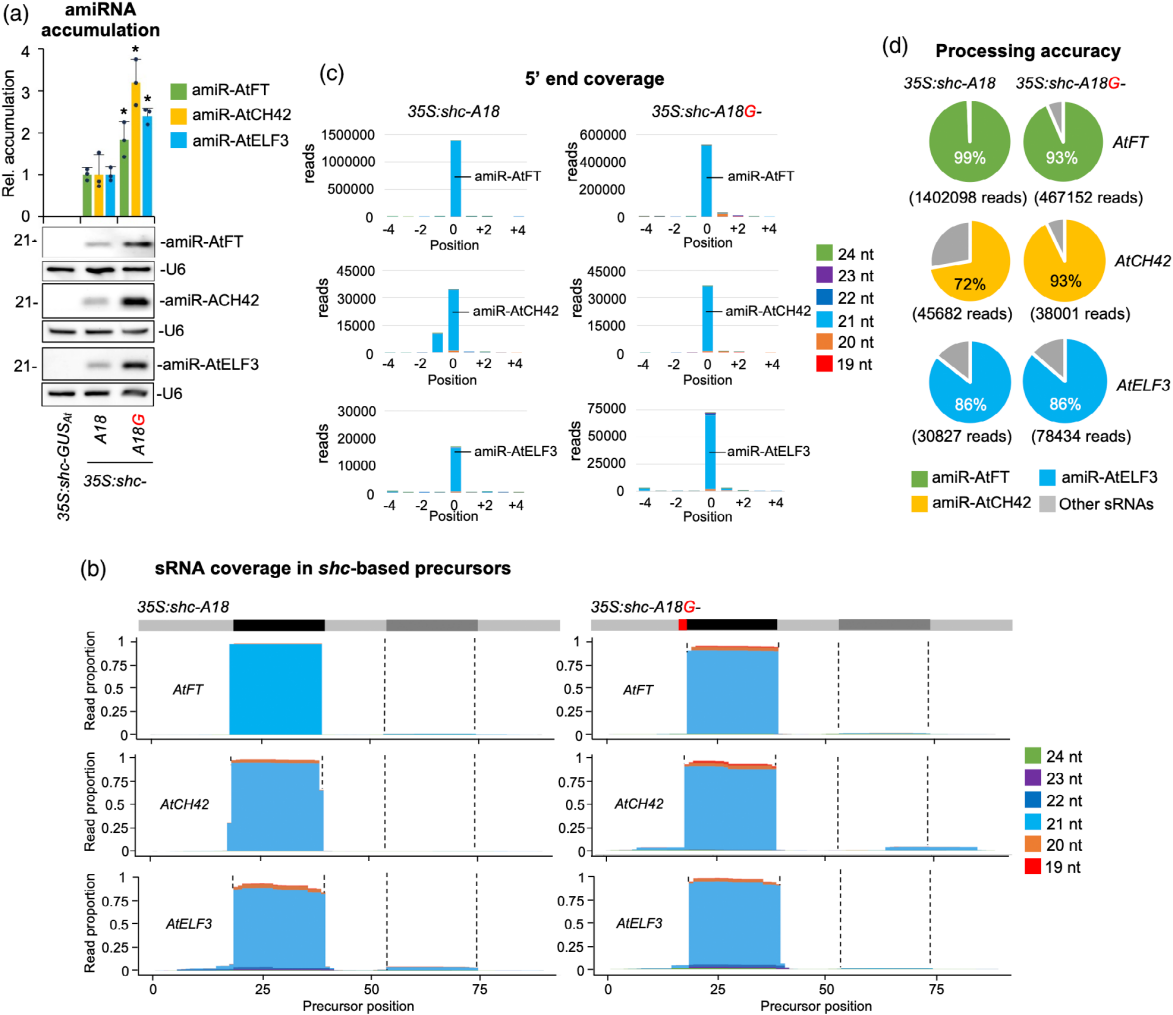
Accumulation and processing of amiR‐AtFT, amiR‐AtCH42, and amiR‐AtELF3 amiRNAs from wild‐type (A18) and A18G‐modified *shc* precursors. (a) Northern blot detection of amiR‐AtFT, amiR‐AtCH42, and amiR‐AtELF3 in RNA preparations from Arabidopsis plants. The graph at top shows the mean + standard deviation (*n* = 3) amiRNA relative accumulation (*35S:shc‐AtFT* = 1, *35S:shc‐AtCH42* = 1 and *35S:shc‐AtELF3* = 1). Bars with an asterisk “*” are significantly different from that of their corresponding *35S:shc‐AtFT, 35S:shc‐AtCH42* or *35S:shc‐AtELF3* control samples. A representative blot from three biological replicates is shown. Each biological replicate is a pool of at least nine independent lines selected randomly. U6 RNA blots are shown as loading controls. (b) Small RNA (sRNA) coverage across *shc*‐based precursors. The *x*‐axis indicates the position on the precursor in nucleotides, from 5′ to 3′. At the top of each plot, the light gray line corresponds to the precursor backbone; the position of the amiRNA and amiRNA* in the precursors are indicated in black and dark gray respectively, and the A18G substitution in red. The *y*‐axis is the sRNA coverage in proportion of reads for each nucleotide position aligning to the positive strand. Coverage of reads of different lengths is shown in separate colors, stacked from bottom to top as indicated in the legend on the right. (c) sRNA 5′ coverage around the artificial miRNA (amiRNA) 5′ end in *shc*‐based precursors. In the *x*‐axis, 0 indicates the 5′ end of the amiRNA, −4 and + 4 indicate 4 nt upstream and downstream of them. They *y*‐axis is the sRNA 5′ coverage in total reads. The light blue portion of the bars at 0 represents authentic amiR‐AtFT, amiR‐AtCH42 and amiR‐AtELF3 reads. Other details are as in (b). (d) amiRNA processing accuracy from *shc*‐based precursors. Pie charts show percentages of reads corresponding to expected, accurately processed 21‐nt mature amiR‐AtFT, amiR‐AtCH42 and amiR‐AtELF3 (green, yellow and light blue sectors, respectively) or to other 19‐24‐nt sRNAs (gray sectors).

Finally, amiRNA accumulation and precursor processing were compared in Arabidopsis lines expressing amiR‐AtFT, amiR‐AtCH42, and amiR‐ELF3 from wild‐type or A18G‐modified *shc* precursors (Figure 7). As shown by northern blot analysis, lines expressing amiRNAs from A18G‐modified *shc* precursors accumulated significantly higher levels of amiRNAs, which migrated as single, discrete bands (Figure 7a). Specifically, accumulation increased by approximately 80, 220, and 140% for amiR‐AtFT, amiR‐AtCH42, and amiR‐AtELF3, respectively, relative to the wild‐type precursor. To analyze the accuracy of the processing and to confirm the presence of authentic amiRNAs, high‐throughput sequencing of sRNAs was performed from Arabidopsis lines expressing each amiRNA from wild‐type or A18G‐modified *shc* precursors. Read coverage profiles revealed that sRNAs mapped almost exclusively to the predicted amiRNA/amiRNA* regions, with negligible reads along the remaining precursor backbone (Figure 7b). As with amiR‐NbDXS‐derived constructs, the distribution was strongly biased toward 21‐nt sRNAs corresponding to the expected mature amiRNAs, with very limited contributions from other size classes. When reads were anchored to the amiRNA 5′ termini, both wild‐type and A18G precursors showed a dominant peak at position 0, demonstrating highly precise DCL1 cleavage (Figure 7c). Importantly, processing accuracy was uniformly high across all amiRNAs, very similar for amiR‐AtFT and amiR‐AtELF3, and slightly higher for amiR‐AtCH42 produced from *shc‐A18G* precursors (Figure 7d).

These analyses indicate that the A18G‐modified precursor supports accurate and efficient DCL1 processing to release highly abundant amiRNAs. Altogether, these results show that stable expression of amiRNAs from the A18G‐modified *shc* precursors produces increased levels of accurately processed amiRNAs for enhanced target silencing efficacy and specificity in transgenic Arabidopsis plants.

## DISCUSSION

In this study, we show that restoring base pairing at the nucleotide immediately upstream of the DCL1 first cleavage site in both the native *AtMIR390a* and engineered *shc* precursors remarkably increases miRNA accumulation. These findings highlight the critical influence of precursor architecture on processing efficiency and identify a novel *shc‐A18G*‐modified precursor with enhanced silencing activity across different species.

Systematic mutational analyses of several plant miRNA precursors revealed that efficient, high‐fidelity miRNA biogenesis in plants depends on the structure of the precursor, particularly on the basal stem (Bajczyk et al., 2023; Li & Yu, 2021; Mateos et al., 2010; Song et al., 2010; Werner et al., 2010). In particular, earlier work has shown that the four positions flanking the miRNA/miRNA* duplex in natural Arabidopsis and eudicot miRNA precursors are usually base‐paired and display position‐specific sequence biases (Rojas et al., 2020). Interestingly, disrupting or restoring these pairings can substantially alter miRNA levels, with both the identity of the nucleotide and its precise position within the precursor affecting processing efficiency. For example, replacing the naturally mismatched 5′‐U in *AtMIR172a* with a canonical C‐G pair increased miR172a accumulation by 130%, whereas breaking adjacent pairs in *AtMIR172a* or *AtMIR164c* severely impaired processing (Mateos et al., 2010; Rojas et al., 2020). Conversely, introducing a mismatch at position 23 of *AtMIR164c* consistently reduced miRNA biogenesis (Rojas et al., 2020), and mutating the U‐G pair at position 13/78 (U‐G) in the basal stem of *AtMIR390a* significantly decreased miR390a levels (Cuperus, Montgomery, et al., 2010). Altogether, these findings support the idea that specific structural features of endogenous *miRNA* precursors are critical for efficient processing. Our present data add position 18 of *AtMIR390a* to this catalog, as pairing this nucleotide located immediately upstream to the mature miRNA substantially boosts miR390a accumulation. Remarkably, genome‐wide analyses indicate that this site is generally paired across eudicots (Rojas et al., 2020), suggesting that the wild‐type *AtMIR390a* architecture may have evolved not to maximize miR390a accumulation but to balance miR390a with *TAS3a* transcript levels. Such homeostasis is essential for proper accumulation of *TAS3a*‐derived trans‐acting siRNAs, which fine‐tune auxin signaling and govern developmental processes such as leaf polarity and patterning, lateral‐root formation, the timing of vegetative phase change and floral development (Adenot et al., 2006; Fahlgren et al., 2006; Garcia et al., 2006; Marin et al., 2010).

Because A18G pairing enhances miR390a production (Figure 1b), we tested whether the same substitution would improve amiRNA biogenesis for targeted gene silencing. In both the full‐length *AtMIR390a* and the recently described 89‐nt *shc* minimal precursor (Cisneros et al., 2023), A18G significantly increased amiRNA levels. In addition, systematic mutagenesis of *shc* revealed that converting any of the three natural mismatches at positions 12, 15, and 18 into a G–C pairs significantly increased mature amiRNA levels, whereas pairing the second DCL1 cleavage site (U51) had no effect (Figures 2, 3). The enhanced biogenesis likely reflects the higher thermodynamic stability of G–C pairs, which may rigidify the local stem and promote precise DCL1 activity. This interpretation aligns with genome‐wide analyses showing that plant miRNA precursors are enriched for G–C/C–G pairs around the miRNA/miRNA* duplex (Rojas et al., 2020). Interestingly, combining two or three pairing mutations did not yield additive benefits (Figure 4), indicating that the *shc* precursor reaches a saturation point beyond which additional basal stem stabilization no longer accelerates DCL1 processing. Our working model is that the introduction of a G–C pair at position 18:73 already optimizes the basal stem architecture for DCL1 recognition and cleave, bringin processing efficiency close to its upper limit. Further closure of nearby mismatches is therefore unlikely to enhance output. An alternative explanation could be that making the stem too perfectly base‐paired may redirect the precursor toward other Dicer‐like enzymes (DCL2, DCL3, and DCL4), which preferentially process long, perfectly paired dsRNAs into distinct size classes—22‐nt for DCL2, 24‐nt for DCL3, and predominantly 21‐nt for DCL4 (Qi et al., 2005). However, our RNA blot analyses revealed single, discrete ~21‐nt amiRNA bands without additional 22‐ or 24‐nt species, and we did not observe any reduction in 21‐nt accumulation in the double or triple mutants (Figure 4 and Figure S3), arguing against misrouting to other DCLs. These results suggest that DCL1 remains the primary processor of these optimized precursors and that efficient recognition requires a balance between stem stability and the conformational flexibility mediated by HYL1 and SERRATE. Importantly, sRNA deep sequencing confirmed that DCL1 processing of the A18G‐modified precursors is as accurate as that of the wild‐type *shc* scaffold, with a very high proportion of reads correspond to the intended 21‐nt amiRNA, with negligible alternative products (Figures 5 and 7). This high fidelity limits the release of ectopic sRNAs and therefore minimizes potential off‐target effects. Moreover, no 21‐nt secondary siRNAs in phase with the expected cleavage site were detected along the cognate target transcripts (Data S1), indicating that A18G‐mediated silencing does not trigger RDR6‐dependent transitivity and further reinforcing its specificity.

In conclusion, the *shc‐A18G* backbone constitutes a minimal, high‐efficiency platform for diverse gene silencing applications. Its robust performance in *N. benthamiana* and Arabidopsis, across multiple guide sequences, confirms its broad utility. Moreover, the accompanying high‐throughput B/c vectors, engineered with the A18G basal stem, simplify amiRNA construct assembly and cut oligonucleotide costs, an advantage for large‐scale functional genomics screens (Hauser et al., 2013; Jover‐Gil et al., 2014; Zhang et al., 2018), also significantly reducing the synthesis costs. Beyond basic research, this optimized amiRNA toolkit offers promise for agriculture, both in transgenic crops and in exogenous amiRNA treatments. Highly specific art‐sRNA technologies represent an important step toward next‐generation crops with improved resilience to environmental stresses and climate change.

## METHODS

### Plant species and growth conditions

*N. benthamiana* plants were cultivated in growth chambers maintained at 25°C with a 12 h light/12 h dark photoperiod. *A. thaliana* ecotype Columbia‐0 (Col‐0) was grown at 22°C under a 16 h light/8 h dark photoperiod, except for the *AtELF3* knock‐down experiment, in which plants were grown under a short‐day regime of 8 h light/16 h dark photoperiod. Arabidopsis transformation was conducted via the floral dip method using *Agrobacterium tumefaciens* strain GV3101 as previously described (Clough & Bent, 1998). Selection and propagation of T1 transgenic lines followed standard protocols (López‐Dolz et al., 2020). Plant images were captured using a Nikon D3000 digital camera equipped with an AF‐S DX NIKKOR 18–55 mm f/3.5–5.6G VR lens.

### Arabidopsis phenotyping

Phenotypic analyses in *A. thaliana* were conducted in a blind manner as previously described (López‐Dolz et al., 2020). Hypocotyl length was quantified from photographs of seedlings laid flat on agar plates alongside a ruler. Images were analyzed in ImageJ (Abramoff et al., 2004) by setting a scale based on the ruler, tracing hypocotyls using the segmented line tool, and extracting length values. Average hypocotyl lengths and standard deviations were calculated from these measurements. Flowering time was determined as the number of days from seed plating to the opening of the first floral bud (‘days to flowering’). A line was classified as exhibiting the “FT” phenotype if its flowering time exceeded the average value observed in the *35S:shc‐GUS*_*At*_ control set. The “CH42” phenotype was assessed in 10‐day‐old seedlings and categorized as “weak,” “intermediate,” or “severe” based on the number of leaf primordia: more than two leaves (weak), exactly two leaves (intermediate), or no true leaves (severe; only cotyledons present). The “ELF3” phenotype is scored in 10‐day‐old seedlings and was defined as a higher “hypocotyl” value when compared with the average hypocotyl length value of the *35S:shc‐GUS*_*At*_ control set.

### Artificial small RNA design

P‐SAMS script (https://github.com/carringtonlab/psams) (Fahlgren et al., 2016), configured to return unlimited optimal results, was used to obtain a list of optimal amiRNAs targeting *AtELF3* with high specificity (Data S2). Off‐target filtering was applied using the *A. thaliana* transcriptome Araport 11 (https://ftp.ncbi.nlm.nih.gov/genomes/all/GCF/000/001/735/GCF_000001735.4_TAIR10.1/) (Cheng et al., 2017) to enhance amiRNA specificity. AmiR‐GUS_Nb_, amiR‐NbSu, amiR‐NbDXS, amiR‐GUS_At_, amiR‐AtFT, and amiR‐AtCH42 guide sequences were described before (Cisneros et al., 2022; López‐Dolz et al., 2020; Schwab et al., 2006).

### DNA constructs

Oligonucleotides AC‐1268 and AC‐1269 were annealed and ligated into *pENTR‐D‐TOPO* to generate *pENTR‐BS‐AtMIR390a‐A18G‐BB* including *AtMIR390a* basal stem sequence interrupted by two inverted *Bsa*I restriction sites. The *BS‐AtMIR390a‐A18G‐BB* cassette from *pENTR‐AtMIR390a‐A18G‐BB* was transferred by LR recombination into *pMDC32B* (Carbonell et al., 2014), a version of *pMDC32* (Curtis & Grossniklaus, 2003) with a mutated *Bsa*I site, to generate *pMDC32B‐BS‐AtMIR390a‐A18G‐BB*. The B/c cassette was amplified from *pENTR‐AtMIR390a‐B/c* (Addgene plasmid #51778) with oligonucleotides AC‐1270 and AC‐1271, and ligated into *pENTR‐D‐TOPO*. Finally, the B/c cassette was excised by *Bsa*I digestion and inserted into *Bsa*I‐digested *pENTR‐BS‐AtMIR390a‐A18G‐BB* and *pMDC32B‐BS‐AtMIR390a‐A18G‐BB* to generate *pENTR‐BS‐AtMIR390a‐A18G‐B/c* (Addgene plasmid 246 715) and *pMDC32B‐BS‐AtMIR390a‐A18G‐B/c* (Addgene plasmid 246 716) which were deposited at Addgene (http://www.addgene.org/).

Constructs *35S:shc‐GUS*_*Nb*_, *35S:AtMIR390a*, *35S:shc‐C40G/U51C‐NbSu*, *35S:shc‐U51G‐NbSu*, *35S:shc‐C40A‐NbSu*, *35S:shc‐C40U/U51A‐NbSu, 35S:shc‐C40U/U51G‐NbSu*, *35S:shc‐C40G‐NbSu, 35S:shc‐C40G/U51C‐NbDXS*, *35S:shc‐U51G‐NbDXS, 35S:shc‐C40A‐NbDXS*, *35S:shc‐C40U/U51A‐NbDXS*, *35S:shc‐C40U/U51G‐NbDXS*, *35S:shc‐C40G‐NbDXS*, *35S:shc‐GUS*_*At*_, *35S:shc‐AtELF3*, were obtained by ligating annealed oligonucleotide pairs AC‐800/AC‐801, AC‐1272/AC‐1273, AC‐1114/AC‐1115, AC‐982/AC‐983, AC‐1116/AC‐117, AC‐1118/AC‐1119, AC‐1120/AC‐1121, AC‐1122/AC‐1123, AC‐1124/AC‐1125, AC‐886/AC‐887, AC‐1126/AC‐1127, AC‐1128/AC‐1129, AC‐1130/AC‐1131, AC‐1132/AC‐1133, AC‐1180/AC‐1181, AC‐1280/AC‐1281, respectively, into *pMDC32B‐BS‐AtMIR390a‐B/c* (Addgene plasmid #199560) (Cisneros et al., 2023).

Constructs *35S:AtMIR390a‐A18G*, *35S:AtMIR390a‐A18G‐NbSu*, *35S:AtMIR390a‐A18G‐NbDXS*, *35S:shc‐A18G‐NbSu*, *35S:shc‐C73U‐NbSu*, *35S:shc‐A18U/C73A‐NbSu*, *35S:shc‐A18U/C73G‐NbSu*, *35S:shc‐A18U/C73G‐NbSu*, *35S:shc‐A18G/C73U‐NbSu*, *35S:shc‐A18C/C73G‐NbSu*, *35S:shc‐A18G‐NbDXS*, *35S:shc‐A18C/C73G‐NbDXS*, *35S:shc‐C73U‐NbDXS*, *35S:shc‐A18U/C73A‐NbDXS*, *35S:shc‐A18U/C73G‐NbDXS*, *35S:shc‐A18G/C73U‐NbDXS*, *35S:shc‐A12G‐NbSu*, *35S:shc‐A12C/C79G‐NbSu*, *35S:shc‐C79U‐NbSu*, *35S:shc‐A12U/C79A‐NbSu*, *35S:shc‐A12U/C79G‐NbSu*, *35S:shc‐A12G/C79U‐NbSu*, *35S:shc‐A12G‐NbDXS*, *35S:shc‐A12C/C79G*, *35S:shc‐C79U‐NbDXS*, 3*5S:shc‐A12U/C79A‐NbDXS*, *35S:shc‐A12U/C79G‐NbDXS*, *35S:shc‐A12G/C79U‐NbDXS*, *35S:shc‐U15G/U76C‐NbSu*, *35S:shc‐U15C/U76G‐NbSu*, *35S:shc‐U15A‐NbSu*, *35S:shc‐U76A‐NbSu*, *35S:shc‐U76G‐NbSu*, *35S:shc‐U15G‐NbSu*, *35S:shc‐U15G/U76C‐NbDXS*, *35S:shc‐U15C/U76G‐NbDXS*, *35S:shc‐U15A‐NbDXS*, *35S:shc‐U76A‐NbDXS*, *35S:shc‐U76G‐NbDXS*, *35S:shc‐U15G‐NbDXS*, *35S:shc‐A12G/U15G‐NbDXS*, *35S:shc‐A12G/A18G‐NbDXS*, *35S:shc‐U15G/A18G‐NbDXS*, *35S:shc‐A12G/U15G/A18G‐NbDXS*, were obtained by ligating annealed oligonucleotide pairs AC‐1274/AC‐1275, AC‐1286/AC‐1287, AC‐1288/AC‐1289, AC‐949/AC‐950, AC‐951/AC‐952, AC‐953/AC‐954, AC‐955/AC‐956, AC‐957/AC‐958, AC‐959/AC‐960, AC‐961/AC‐962, AC‐878/AC‐879, AC‐969/AC‐970, AC‐963/AC‐964, AC‐965/AC‐966, AC‐967/AC‐968, AC‐974/AC‐975, AC‐1150/AC‐1151, AC‐1152/AC‐1153, AC‐1154/AC‐1155, AC‐1158/AC‐1159, AC‐1156/AC‐1157, AC‐882/AC‐883, AC‐1160/AC‐1161, AC‐1162/AC‐1163, AC‐1164/AC‐1165, AC‐1168/AC‐1169, AC‐1166/AC‐1167, AC‐1077/AC‐1078, AC‐1134/AC‐1135, AC‐976/AC‐977, AC‐1136/AC‐1137, AC‐1140/AC‐1141, AC‐1138/AC‐1139, AC‐1085/AC‐1086, AC‐1142/AC‐1143, AC‐884/AC‐885, AC‐1144/AC‐1145, AC‐1148/AC‐1149, AC‐1146/AC‐1147, AC‐1237/AC‐1238, AC‐1087/AC‐1088, AC‐1239/AC‐1240, AC‐1241/AC‐1242, respectively, into *pMDC32B‐B/c* (Addgene plasmid #227963) (Cisneros et al., 2025).

Constructs *35S:shc‐A18G‐AtFT*, *35S:shc‐A18G‐AtCH42*, and *35S:shc‐A18G‐AtELF3* were obtained by ligating annealed oligonucleotide pairs AC‐1276/AC‐1277, AC‐1278/AC‐1279, and AC‐1281/AC‐1282, respectively, into *pMDC32B‐BS‐AtMIR390a‐A18G‐B/c* (Addgene plasmid # 246716). A detailed protocol for cloning amiRNAs in new B/c vectors is described in Text S1. Constructs *35S:GUS*, *35S:AtMIR390a‐GUS*_*Nb*_, *35S:AtMIR390a‐NbSu*, *35S:AtMIR390a‐NbDXS*, *35S:shc‐NbSu*, *35S:shc‐NbDXS*, *35S:shc‐AtFT*, and *35S:shc‐AtCH42* were described before (Cisneros et al., 2022; Cisneros et al., 2023; Montgomery et al., 2008). The sequences of all miRNA/amiRNA precursors are listed in Text S2. The sequences of newly developed B/c vectors are listed in Text S3.

### Transient expression of constructs

*Agrobacterium*‐mediated infiltration of constructs into *N. benthamiana* leaves was performed as previously described (Carbonell et al., 2015; Cuperus, Carbonell, et al., 2010). Briefly, single colonies of *A. tumefaciens* strain GV3101 carrying the corresponding binary plasmids were inoculated into 5 ml LB medium supplemented with rifampicin (50 μg ml^−1^) and the vector‐specific kanamycin antibiotic (50 μg ml^−1^), and cultured overnight at 28°C with shaking (200 rpm). Four milliliters of this starter culture were then transferred to 50 ml of fresh LB medium containing the same antibiotics and grown for 4–6 h at 28°C until OD_600_ ≈ 0.5. For *vir* gene induction, bacterial cells were pelleted (5000 × ***g***, 10 min), resuspended in an equal volume of *vir*‐induction medium (M9 salts supplemented with 20 g L^−1^ glucose, 10 mm MES pH 5.2, 0.1 mm acetosyringone, 0.1 mm CaCl₂, and 2 mm MgSO₄), and incubated overnight (~14 h) at 28°C with shaking. The following morning, cells were harvested again (5000 × ***g***, 10 min) and resuspended in infiltration buffer (10 mm MgCl₂, 10 mm MES pH 5.2, 150 μm acetosyringone) to a final OD_600_ of 1.0. For co‐infiltration, equal volumes of normalized cultures were mixed prior to use. Two fully expanded leaves per plant were infiltrated on the abaxial side with 1 ml disposable syringes (without needles), and infiltrated areas were collected 48 h post‐infiltration for RNA extraction and downstream analyses.

### Chlorophyll extraction and analysis

Chlorophyll and other pigments were extracted from *N. benthamiana* leaves and analyzed as previously described (Carbonell et al., 2015; López‐Dolz et al., 2020). Briefly, pigments were extracted from 40 mg of infiltrated leaf tissue with 5 ml of 80% (v/v) acetone in the dark at room temperature for 24 h, and the extracts were centrifuged at 1000 × ***g*** for 2 min. One hundred microlitres of the supernatant were diluted 1:2 with 80% (v/v) acetone and transferred to flat‐bottom 96‐well plates. Absorbance was recorded from 400 to 750 nm using either a Multiskan GO microplate reader (Thermo Fisher Scientific, Waltham, MA, USA) with SkanIt Software v3.2 or a SpectraMax M2 microplate reader (Molecular Devices, Sunnyvale, CA, USA) with SoftMax Pro 5 software. Chlorophyll and carotenoid contents were calculated according to Lichtenthaler and Wellburn (1983) using the following equations: chlorophyll a (mg L^−1^) = 12.21 × A_663_ − 2.81 × A_647_; chlorophyll b (mg L^−1^) = 20.13 × A_647_ − 5.03 × A_663_; carotenoids (mg L^−1^) = [1000 × A_470_ − 3.27 × (chlorophyll a) − 104 × (chlorophyll b)]/227. Values were normalized to tissue fresh weight and expressed as mg pigment per g fresh weight.

### Total RNA preparation

Total RNA from *N. benthamiana* leaves or Arabidopsis seedlings or inflorescences was isolated as previously described (Cisneros et al., 2023). Briefly, frozen tissues were ground in liquid nitrogen to a fine powder and immediately homogenized in extraction buffer containing 1 m guanidinium thiocyanate, 1 m ammonium thiocyanate, 0.1 m sodium acetate, 5% (v/v) glycerol, and 38% (v/v) water‐saturated phenol. RNA was subsequently extracted with an equal volume of chloroform, and the aqueous phase was precipitated by adding 0.5 volumes of isopropanol and incubating for 20 min at room temperature. The RNA pellets were washed with 75% ethanol, air‐dried, and resuspended in RNase‐free water, and RNA concentration was verified by spectrophotometric quantification. Each biological replicate consisted of pools of two *N. benthamiana* leaves or 9–12 T1 Arabidopsis seedlings or inflorescences, and three independent biological replicates were analyzed for each construct.

### Real‐time RT‐qPCR

Real‐time RT‐qPCR was performed using the RNA samples previously analyzed by sRNA blotting as described (Cisneros et al., 2025). Briefly, Complementary DNA (cDNA) was synthesized from 500 ng of DNase I‐treated total RNA using the PrimeScript RT Reagent Kit (Perfect Real Time; Takara, Kusatsu, Shiga, Japan) according to the manufacturer's instructions. RNA samples originated from *N. benthamiana* leaves collected 2 dpa or from Arabidopsis T1 seedlings (10 days old) or inflorescences (60 days old) previously analyzed by sRNA blotting. Real‐time RT‐qPCR reactions were carried out in optical 96‐well plates using a QuantStudio 3 Real‐Time PCR System (Thermo Fisher Scientific). Each 20 μL reaction contained 10 μL of 2× TB Green Premix Ex Taq (Takara), 2 μL of diluted cDNA (1:5), 0.4 μL of 50× ROX II Reference Dye, and 300 nm of each gene‐specific primer. The amplification program consisted of an initial denaturation step of 20 sec at 95°C, followed by 40 cycles of 95°C for 3 sec and 60°C for 30 sec, and a final melt‐curve stage of 95°C for 15 sec, 60°C for 1 min, and 95°C for 15 sec. Primer sequences are listed in Table S2. Target mRNA expression was quantified relative to the reference gene *NbPP2A* or *AtACT2* in *N. benthamiana* and Arabidopsis, respectively, using the ΔΔCt comparative method in QuantStudio Design and Analysis software (v1.5.1; Thermo Fisher Scientific). Three independent biological replicates, each with two technical replicates, were analyzed. Each biological replicate consisted of pools of two *N. benthamiana* leaves or 9–12 T1 Arabidopsis seedlings or inflorescences.

### Small RNA blot assays

Total RNA from three independent biological replicates was analyzed, with each biological replicate consisting of pools of two *N. benthamiana* leaves or 9–12 T1 Arabidopsis seedlings or inflorescences. Twenty micrograms of total RNA was separated on 17% polyacrylamide gels (0.5× TBE, 7 m urea) and electrotransferred onto positively charged nylon membranes. DNA or LNA probes were 3′‐labeled with digoxigenin using the second‐generation DIG Oligonucleotide 3′‐End Labeling Kit (Roche, Basel, Basel‐Stadt, Switzerland) and hybridized at 38°C as described (Tomassi et al., 2017). Chemiluminescent detection was performed with DCP‐Star (Roche), and membranes were imaged using an ImageQuant 800 CCD imager (Cytiva, Marlborough, MA, USA). Signal intensities were quantified with ImageQuant TL software (v10.2; Cytiva) from non‐saturating exposures within the linear detection range. All blots used for amiRNA quantification are presented in Figure S3. Probe sequences are listed in Table S2.

### Small RNA sequencing and data analysis

Total RNA quality, purity, and integrity were verified using an Agilent 2100 Bioanalyzer (RNA 6000 Nano kit) prior to sRNA library preparation and single‐end 50‐nt sequencing (SE50) performed by BGI (Hong Kong, China) on a DNBSEQ‐G400 sequencer. Adapter‐trimmed and quality‐filtered reads provided by BGI were collapsed using the FASTX‐Toolkit (http://hannonlab.cshl.edu/fastx_toolkit) to merge identical sequences while preserving read counts. Each unique read was mapped to the forward strand of the corresponding amiRNA precursor (Data S3) using a custom Python script deposited at Github (https://github.com/acarbonell/map_sRNA_reads/) that allowed exact matches without gaps or mismatches, calculating read counts and reads per million mapped reads (RPM). sRNA alignments were visualized using sRNA_Viewer software (Axtell Lab, Pennsylvania State University; https://github.com/MikeAxtell/sRNA_Viewer). Processing accuracy was assessed by quantifying the proportion of 19–24 nt sRNA (+) reads mapping within ±4 nt of the predicted amiRNA guide's 5′ end (Carbonell et al., 2015; Cuperus, Carbonell, et al., 2010).

### Statistical analysis

All statistical analyses are detailed in the corresponding figure legends. For pairwise comparisons, statistical significance was determined using a two‐tailed Student's *t*‐test. Data are presented as mean ± standard deviation (SD) from three independent biological replicates. Each biological replicate consisted of pools of two *N. benthamiana* leaves or 9–12 independent T1 Arabidopsis seedlings or inflorescences. Statistical significance levels are represented as *P* < 0.05 (*). Scatter bar plots were used to display individual data points and variability across replicates.

## ACCESSION NUMBERS

Gene identifiers used in this study are as follows: Arabidopsis: *AtACT2* (AT3G18780), *AtCH42* (AT4G18480), *AtELF3* (AT2G25930), and *AtFT* (AT1G65480). *N. benthamiana: NbSu* (Nbv5.1tr6204879), *NbDXS* (Nbv5.1tr6224823), and *NbPP2A* (Nbv5.1tr6224808). The *Escherichia coli* β‐glucuronidase (GUS) gene sequence corresponds to GenBank accession S69414.1. High‐throughput sequencing data can be found in the Sequence Read Archive (SRA) database under accession number PRJNA957136 and PRJNA1312446.

## AUTHOR CONTRIBUTIONS

J‐JLG did the majority of the experimental work with assistance from PGC, SRR, LDC, and STF. J‐JLG and AC analyzed the data. AC conceived the study, supervised the project, and wrote the manuscript with input from all authors.

## CONFLICT OF INTEREST

None declared.

## Supporting information


**Figure S1.** *BS‐AtMIR390a‐A18G‐B/c*‐based vectors for direct cloning of amiRNAs.
**Figure S2.** Direct amiRNA cloning in *AtMIR390a‐A18G‐B/c* (*Bsa*I/*ccd*B)‐based vectors including a *ccd*B cassette flanked by two *Bsa*I sites.
**Figure S3.** sRNA blots and densitometry analyses.
**Table S1.** Phenotypic penetrance of amiRNAs expressed in *A. thaliana* Col‐0 T1 transgenic.
**Table S2.** Name, sequence, and use of oligonucleotides used in this study.
**Text S1.** Protocol to design and clone amiRNAs in *BS‐AtMIR390a‐A18G‐B/c*‐based vectors *pENTR‐BS‐AtMIR390a‐A18G‐B/c* and *pMDC32B‐BS‐AtMIR390a‐A18G‐B/c*.
**Text S2.** DNA sequence in FASTA format of all precursors used to express amiRNAs in plants.
**Text S3.** DNA sequence of *BS‐AtMIR390a‐A18G‐B/c*‐based vectors used for direct cloning of amiRNAs.

**Data S1.** 21‐nt sRNA reads mapping amiRNA targets in amiRNA‐expressing tissues.

**Data S2.** P‐SAMS designs of art‐sRNA sequences.

**Data S3.** sRNA reads mapping amiRNA precursors in amiRNA‐expressing tissues.

## Data Availability

The data that support the findings of this study are openly available in Sequence Read Archive at https://www.ncbi.nlm.nih.gov/sra, reference number PRJNA1312446.
