# Supplementary material for: Enhanced microRNA accumulation and gene silencing efficiency through optimized precursor base pairing
Source: Plant J. 2026 Jan 8;125(1):e70665. doi: 10.1111/tpj.70665 (PMC12782649; doi:10.1111/tpj.70665)
Supplement: Supplementary file 1 — Figure S1. BS‐AtMIR390a‐A18G‐B/c‐based vectors for direct cloning of amiRNAs. Figure S2. Direct amiRNA cloning in AtMIR390a‐A18G‐B/c (BsaI/ccdB)‐based vectors including a ccdB cassette flanked by two BsaI sites. Figure S3. sRNA blots and densitometry analyses. Table S1. Phenotypic penetrance of amiRNAs expressed in A. thaliana Col‐0 T1 transgenic. Table S2. Name, sequence, and use of oligonucleotides used in this study. Text S1. Protocol to design and clone amiRNAs in BS‐AtMIR390a‐A18G‐B/c‐based vectors pENTR‐BS‐AtMIR390a‐A18G‐B/c and pMDC32B‐BS‐AtMIR390a‐A18G‐B/c. Text S2. DNA sequence in FASTA format of all precursors used to express amiRNAs in plants. Text S3. DNA sequence of BS‐AtMIR390a‐A18G‐B/c‐based vectors used for direct cloning of amiRNAs. [file TPJ-125-0-s001.pdf]

SUPPORTING MATERIAL

Data S1. 21-nt sRNA reads mapping amiRNA targets in amiRNA-expressing tissues.

Data S2. P-SAMS designs of art-sRNA sequences.

Data S3. sRNA reads mapping amiRNA precursors in amiRNA-expressing tissues.

Figure S1. *BS-AtMIR390a-A18G-B/c*-based vectors for direct cloning of amiRNAs.

Figure S2. Direct amiRNA cloning in *AtMIR390a-A18G-B/c* (*Bsa*I/*ccd*B)-based vectors including a *ccd*B cassette flanked by two *Bsa*I sites.

Figure S3. sRNA blots and densitometry analyses.

Table S1. Phenotypic penetrance of amiRNAs expressed in *A. thaliana* Col-0 T1 transgenic.

Table S2. Name, sequence and use of oligonucleotides used in this study.

Text S1. Protocol to design and clone amiRNAs in *BS-AtMIR390a-A18G-B/c*-based vectors *pENTR-BS-AtMIR390a-A18G-B/c* and *pMDC32B-BS-AtMIR390a-A18G-B/c*.

Text S2. DNA sequence in FASTA format of all precursors used to express amiRNAs in plants.

Text S3. DNA sequence of *BS-AtMIR390a-A18G-B/c*-based vectors used for direct cloning of amiRNAs.

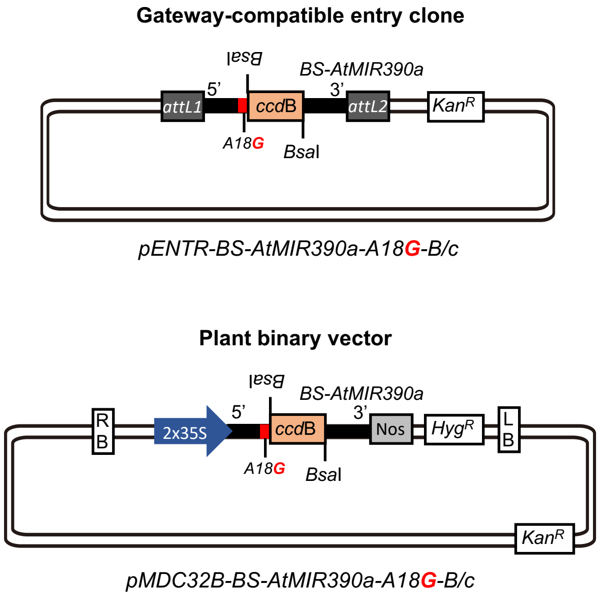

Figure S1. *BS-AtMIR390a-A18G-B/c*-based vectors for direct cloning of amiRNAs. Top, diagram of the Gateway-compatible *pENTR-BS-AtMIR390a-A18G-B/c* entry vector. Bottom, diagram of the *pMDC32B-BS-AtMIR390a-B/c* binary vector for in plant expression of amiRNAs. RB: right border; BS, basal stem; 35S: Cauliflower mosaic virus promoter; *Bsa*I: *Bsa*I recognition site, *ccd*B: gene encoding the gyrase toxin; LB: left border; attL1 and attL2: GATEWAY recombination sites. *Kan*^{R}: kanamycin resistance gene; *Hyg*^{R}: hygromycin resistance gene. The A18G mutation at the 5' BS is shown in red.

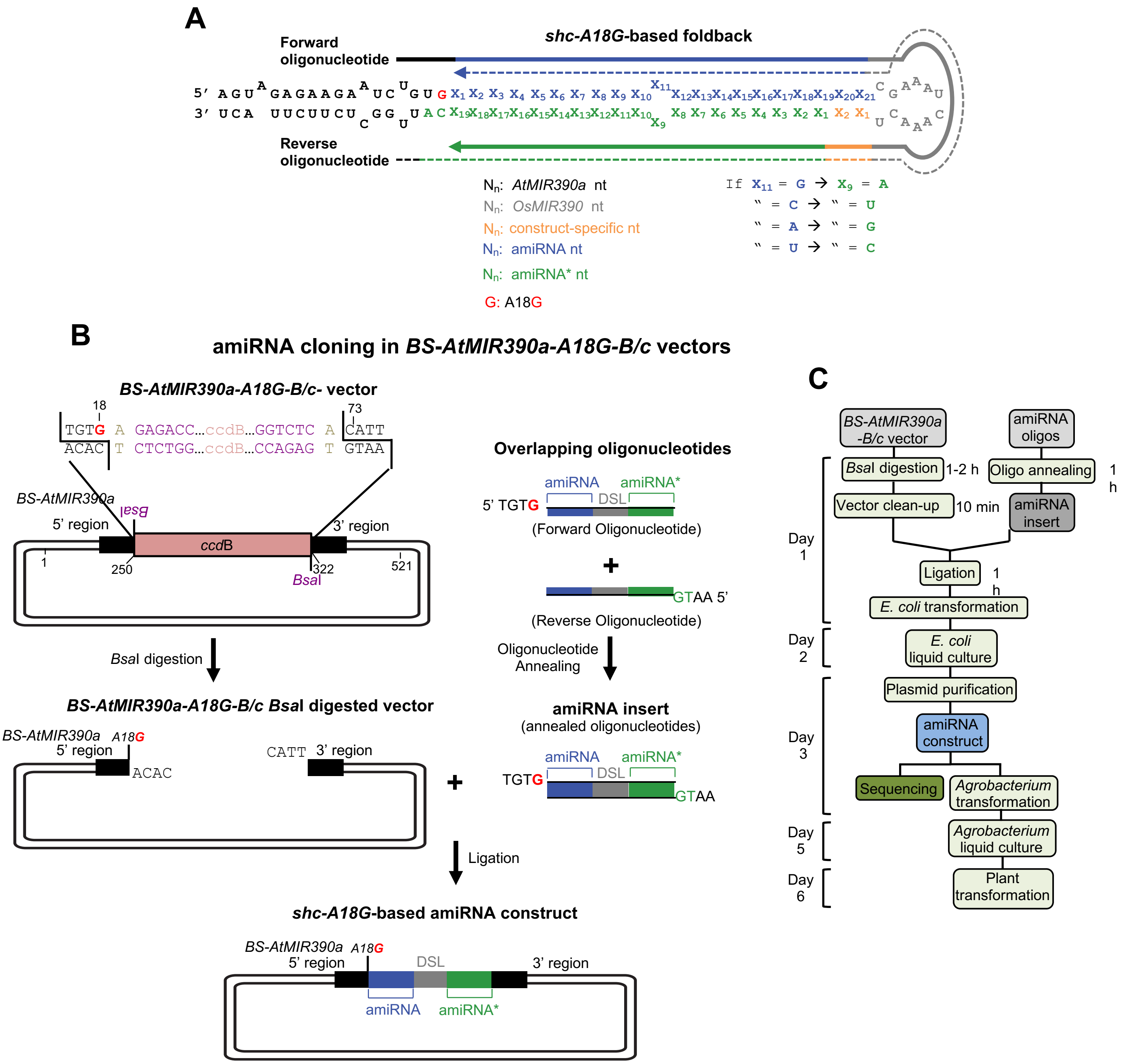

Figure S2. Direct cloning of amiRNAs in vectors containing a modified version of *BS-AtMIR390a-A18G* that includes a *ccd*B cassette flanked by two *Bsa*I sites (*Bsa*I/*ccd*B or 'B/c' vectors). A, Design of two overlapping oligonucleotides for amiRNA cloning in *BS-AtMIR390a-A18G*-based "B/c" vectors including *OsMIR390* DSL sequences to produce a final *shc* precursor. Sequences covered by the forward and the reverse oligonucleotides are represented with continuous or dotted lines, respectively. Nucleotides of *BS-AtMIR390a-A18G* precursor, *OsMIR390*-derived distal stem loop (DSL), amiRNA guide strand and amiRNA* strand are in black, grey, blue and green, respectively, except A18G which is in red. Other nucleotides that may be modified for preserving authentic *OsMIR390a* foldback secondary structure are in orange. Rules for assigning identity to position 9 of the amiRNA* are indicated. B, Diagram of the steps for amiRNA cloning in *BS-AtMIR390a-A18G-B/c* vectors. The amiRNA insert obtained after annealing the two overlapping oligonucleotides has 5'-TGTG and 5'-AATG overhangs and is directly inserted in a directional manner into a *BS-AtMIR390a-A18G-B/c* vector previously linearized with *Bsa*I. Nucleotides of the *Bsa*I sites and those arbitrarily chosen and used as spacers between the *Bsa*I recognition sites and the *BS-AtMIR390a-A18G* sequence are in purple and light brown, respectively. Other details are as described in panel A. C, Flowchart of steps from amiRNA construct generation to plant transformation.

Figure S3. sRNA blots and densitometry analyses.

All northern blots used in this study and the corresponding densitometry analyses are shown for each main figure. Three blots corresponding each to a biological replicate were used for densitometry. Graphs show the mean (n = 3) + standard deviation of miRNA/amiRNA relative accumulation. Bars marked with an asterisk '*' are significantly different from that of the corresponding wild-type precursor control (P < 0.05 in pairwise Student's t-test).

Figure 1

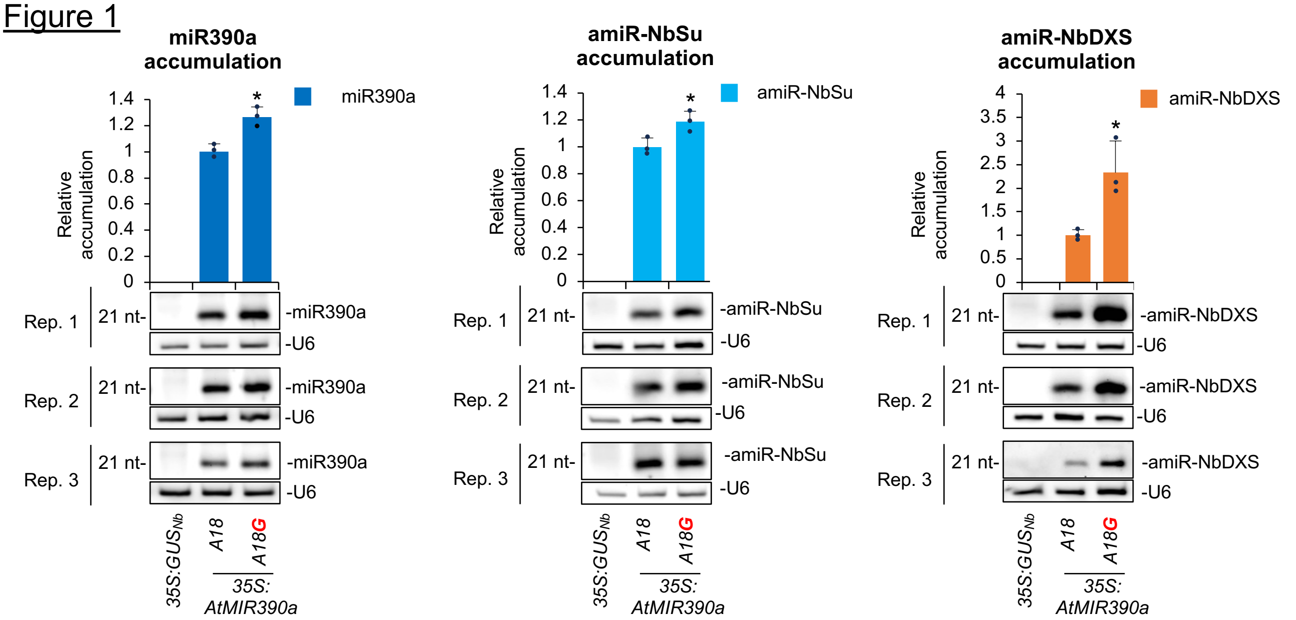

Figure 2

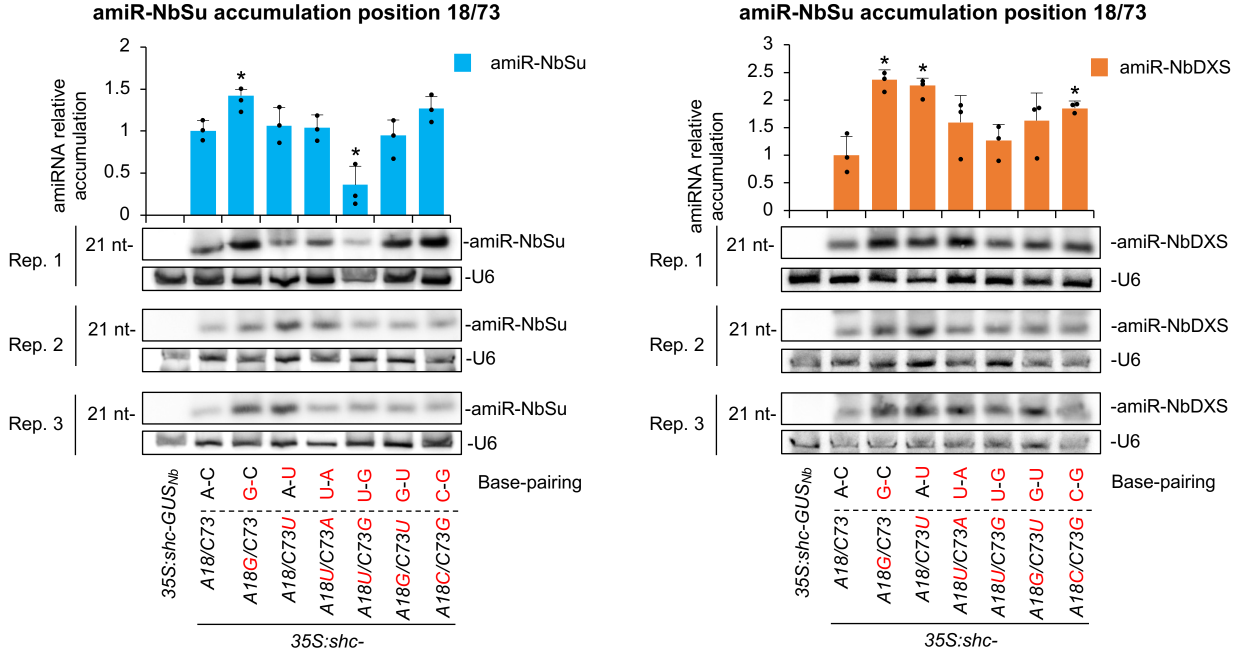

Figure 3

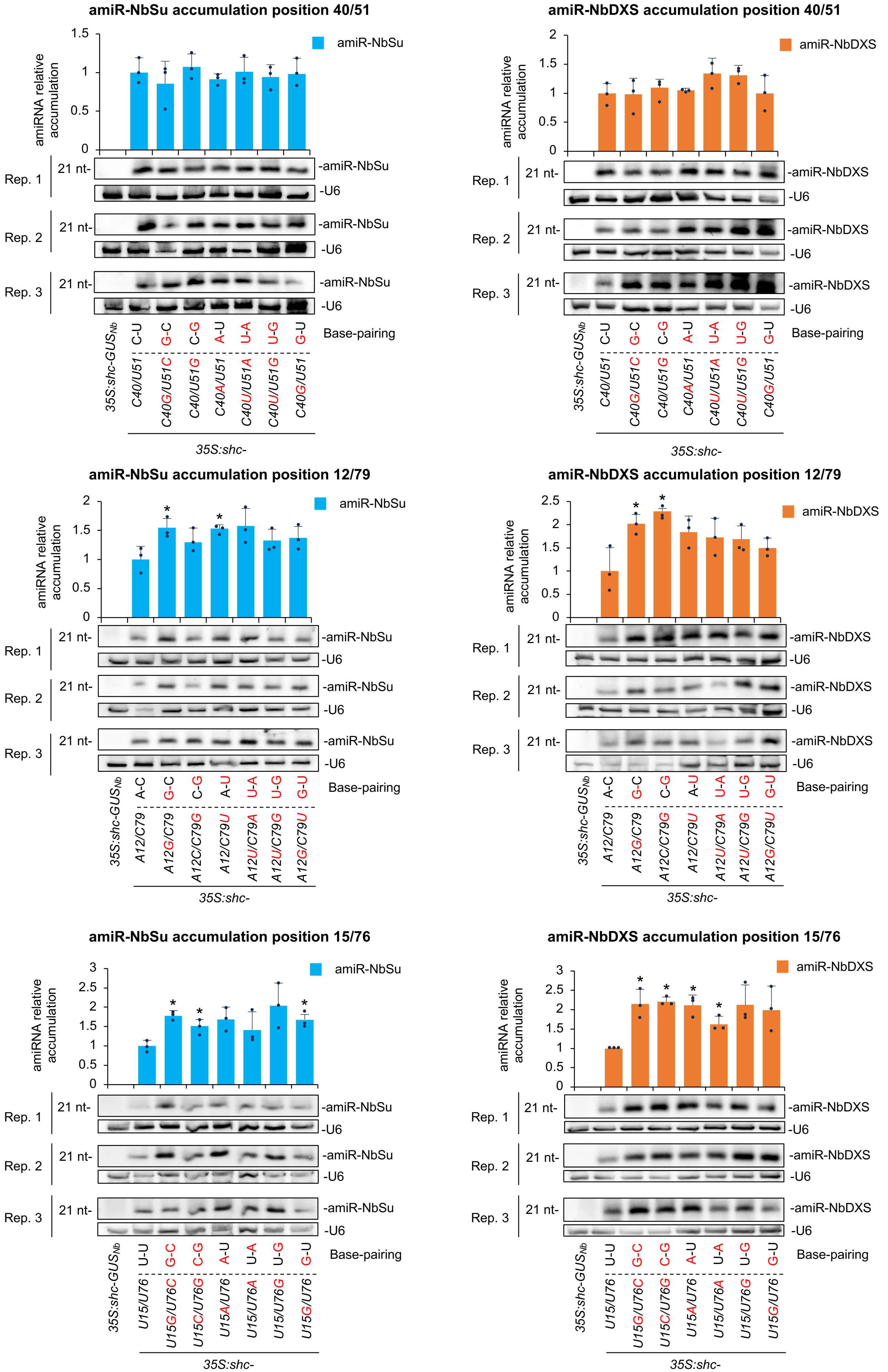

amiR-NbSu accumulation position 40/51
amiRNA relative accumulation
amiR-NbSu
Rep. 1
Rep. 2
Rep. 3
21 nt-
-amiR-NbSu
-U6
Base-pairing
C-U
G-C
C-G
A-U
U-A
U-G
G-U
35S:shc-GUS$_{Nb}$
C40/U51
C40G/U51C
C40/U51G
C40A/U51
C40U/U51A
C40U/U51G
C40G/U51
35S:shc-
amiR-NbDXS accumulation position 40/51
amiRNA relative accumulation
amiR-NbDXS
-amiR-NbDXS
amiR-NbSu accumulation position 12/79
A-C
A12/C79
A12G/C79
A12C/C79G
A12/C79U
A12U/C79A
A12U/C79G
A12G/C79U
amiR-NbDXS accumulation position 12/79
amiR-NbSu accumulation position 15/76
U-U
U15/U76
U15G/U76C
U15C/U76G
U15A/U76
U15/U76A
U15/U76G
U15G/U76
amiR-NbDXS accumulation position 15/76

Figure 4

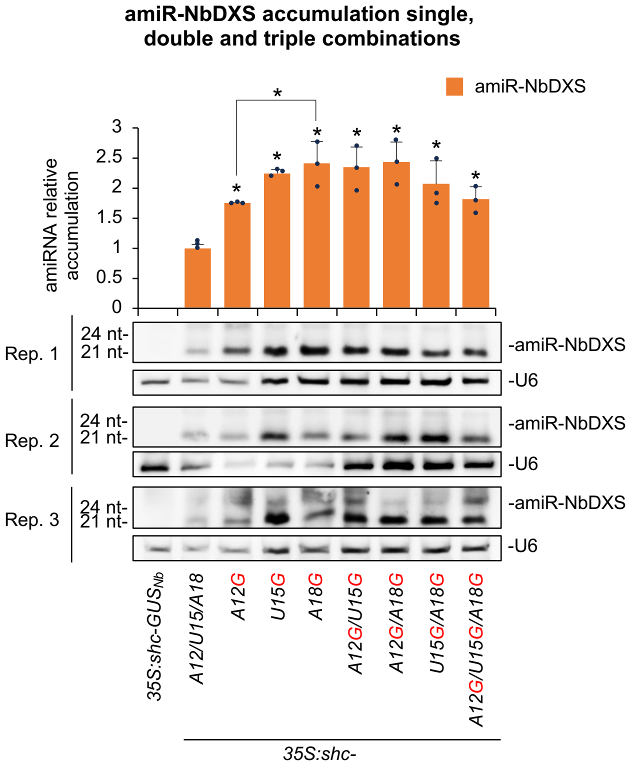

Figure 7

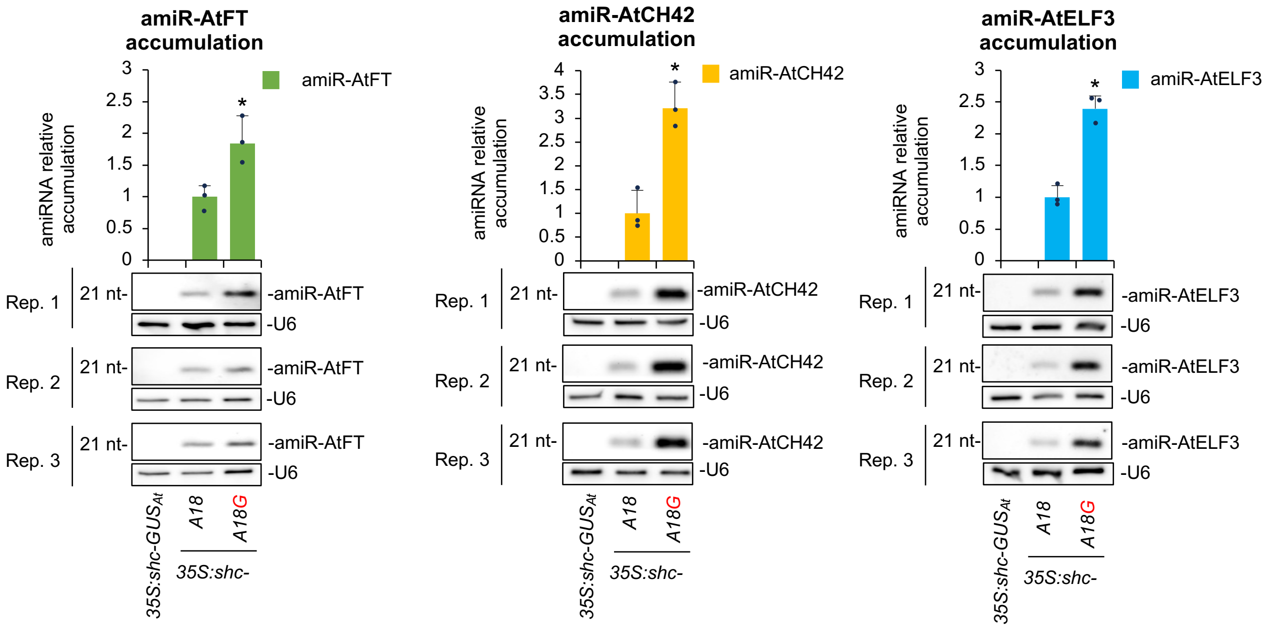

Table S1: Phenotypic penetrance of amiRNAs expressed in *A. thaliana* Col-0 T1 transgenic plants

**Construct**	**T1 analyzed**	**Phenotypic penetrance[a]**
35S:shc-amiR-GUS$_{At}$	44	0%
35S:shc-amiR-AtFT	43	100%
35S:shc-A18G-amiR-AtFT	34	100%
35S:shc-amiR-GUS$_{At}$	278	0%
35S:shc-amiR-AtELF3	398	72.4%
35S:shc-A18G-amiR-AtELF3	335	89%
35S:shc-amiR-GUS$_{At}$	260	0%
35S:shc-amiR-AtCH42	125	100%<br>13.6% weak<br>37.6% intermediate<br>28.8 % severe
35S:shc-A18G-amiR-AtCH42	230	100%<br>4.3% weak<br>29.6% intermediate<br>48.7 % severe

[a] The FT phenotype was defined as a higher 'days to flowering' value when compared to the average 'days to flowering' value of the *35S:pri-amiR-GUS*$_{Ath}$ control set. The ELF3 phenotype is scored in 10 days-old seedlings and was defined as a higher 'hypocotyl' value when compared to the average hypocotyl length value of the *35S:shc-amiR-GUS*$_{At}$ control set. CH42 phenotype is scored in 10 days-old seedling and is considered 'weak', 'intermediate' or 'severe' if seedlings have >2 leaves, exactly two leaves or no leaves (only two cotyledons), respectively.

Table S2. Name, sequence and use of DNA oligonucleotides used in this study.

Name	Sequence	Type*	Construct/Aim
AC-55	AGGGGCCATGCTAATCTTCTC	ssDNA	Probe for U6 detection
AC-157	GGCCTCTTCCTTTATAACCAA	ssDNA	Probe for amiR-AtFT detection
AC-158	AGGGATTTCCGTGACACTTAA	ssDNA	Probe for amiR-AtCH42 detection
AC-159	AAAAATGGCTGAGGCTGATGA	ssDNA	qPCR amplification of *AtACT2* mRNA
AC-160	GAAAAACAGCCCTGGGAGC	ssDNA	
AC-163	CATGCACAAGTAGGGACGGTT	ssDNA	qPCR amplification of *AtCH42* mRNA
AC-164	GTCACGGAAATCCTTTGGGTT	ssDNA	
AC-169	TGGAACAACCTTTGGCAATG	ssDNA	qPCR amplification of *AtFT* mRNA
AC-170	CGACACGATGAATTCCTGCA	ssDNA	
AC-355	GACCCTGATGTTGATGTTCGCT	ssDNA	qPCR amplification of *NbSu* mRNA
AC-356	GAGGGATTTGAAGAGAGATTTC	ssDNA	
AC-359	GGTGGTGGGACTGGTATGAA	ssDNA	qPCR amplification of *NbDXS* mRNA
AC-360	GCAAATCTCACTGGCAGCTT	ssDNA	
AC-365	GACCCTGATGTTGATGTTCGCT	ssDNA	qPCR amplification of *NbPP2A* mRNA
AC-366	GAGGGATTTGAAGAGAGATTTC	ssDNA	
AC-417	G+CGG+GAA+GTC+CAC+CAC+GGT+TA	ssLNA	Probe for amiR-NbSu detection
AC-418	C+TGT+TAG+GAA+CCC+GCG+GTT+TA	ssLNA	Probe for amiR-NbDXS detection
AC-587	AAGGGATCAGGTCAAGGCGAA	ssDNA	Probe for amiR-AtPDS3 detection
AC-591	ATTGCTGCATCACCGGATCT	ssDNA	qPCR amplification of *AtELF3*
AC-592	TCACCCCTTTGTTTGACGACA	ssDNA	
AC-800	TGTATCTTGTAACGCGCTTTCCCAGCGAAATCAAACTCT GGGAAAGCTCGTTACAAGA	ssDNA	Generation of *35S:shc-*GUS_{Nb}
AC-801	AATGTCTTGTAACGAGCTTTCCCAGAGTTTGATTTCGCTG GGAAAGCGCGTTACAAGA	ssDNA	
AC-878	TGTAAGTAGAGAAGAATCTGTGTAAACCGCGGGTTCCTA ACAGCGAAATCAAACTCTGTTAGGAAACCGCGGTTTACA TTGGCTCTTCTTACT	ssDNA	Generation of *35S:shc-A18G-NbDXS*
AC-879	AATGAGTAAGAAGAGCCAATGTAAACCGCGGTTTCCTA ACAGAGTTTGATTTCGCTGTTAGGAACCCGCGGTTTACA CAGATTCTTCTCTACT	ssDNA	
AC-882	TGTAAGTAGAGAAGAGTCTGTATAAACCGCGGGTTCCTA ACAGCGAAATCAAACTCTGTTAGGAAACCGCGGTTTACA TTGGCTCTTCTTACT	ssDNA	Generation of *35S:shc-A12G-NbDXS*
AC-883	AATGAGTAAGAAGAGCCAATGTAAACCGCGGTTTCCTA ACAGAGTTTGATTTCGCTGTTAGGAACCCGCGGTTTATA CAGACTCTTCTCTACT	ssDNA	
AC-884	TGTAAGTAGAGAAGAATCAGTATAAACCGCGGGTTCCT AACAGCGAAATCAAACTCTGTTAGGAAACCGCGGTTTAC ATTGGCTCTTCTTACT	ssDNA	Generation of *35S:shc-U15A-NbDXS*
AC-885	AATGAGTAAGAAGAGCCAATGTAAACCGCGGTTTCCTA ACAGAGTTTGATTTCGCTGTTAGGAACCCGCGGTTTATA CTGATTCTTCTCTACT	ssDNA	
AC-886	TGTATAAACCGCGGGTTCCTAACAGCGAAATCAAACGCT GTTAGGAAACCGCGGTTTA	ssDNA	Generation of *35S:shc-U51G-NbDXS*
AC-887	AATGTAAACCGCGGTTTCCTAACAGCGTTTGATTTCGCT GTTAGGAACCCGCGGTTTA	ssDNA	
AC-949	TGTAAGTAGAGAAGAATCTGTGTAACCGTGGTGGACTTC CCGCCGAAATCAAACTGCGGGAAGTCAACCACGGTTAC ATTGGCTCTTCTTACT	ssDNA	Generation of *35S:shc-A18G-NbSu*
AC-950	AATGAGTAAGAAGAGCCAATGTAACCGTGGTTGACTTCC CGCAGTTTGATTTCGGCGGGAAGTCCACCACGGTTACAC AGATTCTTCTCTACT	ssDNA	
AC-951	TGTAAGTAGAGAAGAATCTGTATAACCGTGGTGGACTTC CCGCCGAAATCAAACTGCGGGAAGTCAACCACGGTTAT ATTGGCTCTTCTTACT	ssDNA	Generation of *35S:shc-C73U-NbSu*

AC-952	AATGAGTAAGAAGAGCCAATATAACCGTGGTTGACTTCCCGCAGTTTGATTTCGGCGGGAAGTCCACCACGGTTATACAGATTCTTCTCTACT	ssDNA	
AC-953	TGTAAGTAGAGAAGAATCTGTTTAACCGTGGTGGACTTCCCGCCGAAATCAAACTGCGGGAAGTCAACCACGGTTAAATTGGCTCTTCTTACT	ssDNA	Generation of *35S:shc-A18U/C73A-NbSu*
AC-954	AATGAGTAAGAAGAGCCAATTTAACCGTGGTTGACTTCCCGCAGTTTGATTTCGGCGGGAAGTCCACCACGGTTAAACAGATTCTTCTCTACT	ssDNA	
AC-955	TGTAAGTAGAGAAGAATCTGTTTAACCGTGGTGGACTTCCCGCCGAAATCAAACTGCGGGAAGTCAACCACGGTTAGATTGGCTCTTCTTACT	ssDNA	Generation of *35S:shc-A18U/C73G-NbSu*
AC-956	AATGAGTAAGAAGAGCCAATCTAACCGTGGTTGACTTCCCGCAGTTTGATTTCGGCGGGAAGTCCACCACGGTTAAACAGATTCTTCTCTACT	ssDNA	
AC-957	TGTAAGTAGAGAAGAATCTGTGTAACCGTGGTGGACTTCCCGCCGAAATCAAACTGCGGGAAGTCAACCACGGTTATATTGGCTCTTCTTACT	ssDNA	Generation of *35S:shc-A18G/C73U-NbSu*
AC-958	AATGAGTAAGAAGAGCCAATATAACCGTGGTTGACTTCCCGCAGTTTGATTTCGGCGGGAAGTCCACCACGGTTACACAGATTCTTCTCTACT	ssDNA	
AC-959	TGTAAGTAGAGAAGAATCTGTCTAACCGTGGTGGACTTCCCGCCGAAATCAAACTGCGGGAAGTCAACCACGGTTAGATTGGCTCTTCTTACT	ssDNA	Generation of *35S:shc-A18C/C73G-NbSu*
AC-960	AATGAGTAAGAAGAGCCAATCTAACCGTGGTTGACTTCCCGCAGTTTGATTTCGGCGGGAAGTCCACCACGGTTAGACAGATTCTTCTCTACT	ssDNA	
AC-961	TGTAAGTAGAGAAGAATCTGTATAAACCGCGGGTTCCTAACAGCGAAATCAAACTCTGTTAGGAAACCGCGGTTTATATTGGCTCTTCTTACT	ssDNA	Generation of *35S:shc-C73U-NbDXS*
AC-962	AATGAGTAAGAAGAGCCAATATAAACCGCGGTTTCCTAACAGAGTTTGATTTCGCTGTTAGGAACCCGCGGTTTATACAGATTCTTCTCTACT	ssDNA	
AC-963	TGTAAGTAGAGAAGAATCTGTTTAAACCGCGGGTTCCTAACAGCGAAATCAAACTCTGTTAGGAAACCGCGGTTTAAATTGGCTCTTCTTACT	ssDNA	Generation of *35S:shc-A18U/C73A-NbDXS*
AC-964	AATGAGTAAGAAGAGCCAATTTAAACCGCGGTTTCCTAACAGAGTTTGATTTCGCTGTTAGGAACCCGCGGTTTAAACAGATTCTTCTCTACT	ssDNA	
AC-965	TGTAAGTAGAGAAGAATCTGTTTAAACCGCGGGTTCCTAACAGCGAAATCAAACTCTGTTAGGAAACCGCGGTTTAGATTGGCTCTTCTTACT	ssDNA	Generation of *35S:shc-A18U/C73G-NbDXS*
AC-966	AATGAGTAAGAAGAGCCAATCTAAACCGCGGTTTCCTAACAGAGTTTGATTTCGCTGTTAGGAACCCGCGGTTTAAACAGATTCTTCTCTACT	ssDNA	
AC-967	TGTAAGTAGAGAAGAATCTGTGTAAACCGCGGGTTCCTAACAGCGAAATCAAACTCTGTTAGGAAACCGCGGTTTATATTGGCTCTTCTTACT	ssDNA	Generation of *35S:shc-A18G/C73U-NbDXS*
AC-968	AATGAGTAAGAAGAGCCAATATAAACCGCGGTTTCCTAACAGAGTTTGATTTCGCTGTTAGGAACCCGCGGTTTACACAGATTCTTCTCTACT	ssDNA	
AC-969	TGTAAGTAGAGAAGAATCTGTCTAAACCGCGGGTTCCTAACAGCGAAATCAAACTCTGTTAGGAAACCGCGGTTTAGATTGGCTCTTCTTACT	ssDNA	Generation of *35S:shc-A18C/C73G-NbDXS*
AC-970	AATGAGTAAGAAGAGCCAATCTAAACCGCGGTTTCCTAACAGAGTTTGATTTCGCTGTTAGGAACCCGCGGTTTAGACAGATTCTTCTCTACT	ssDNA	
AC-974	TGTAAGTAGAGAAGAGTCTGTATAACCGTGGTGGACTTCCCGCCGAAATCAAACTGCGGGAAGTCAACCACGGTTACATTGGCTCTTCTTACT	ssDNA	Generation of *35S:shc-A12G-NbSu*
AC-975	AATGAGTAAGAAGAGCCAATGTAACCGTGGTTGACTTCCCGCAGTTTGATTTCGGCGGGAAGTCCACCACGGTTATACAGACTCTTCTCTACT	ssDNA	
AC-976	TGTAAGTAGAGAAGAATCAGTATAACCGTGGTGGACTTCCCGCCGAAATCAAACTGCGGGAAGTCAACCACGGTTACATTGGCTCTTCTTACT	ssDNA	Generation of *35S:shc-U15A-NbSu*

AC-977	AATGAGTAAGAAGAGCCAATGTAACCGTGGTTGACTTCCCGCAGTTTGATTTCGGCGGGAAGTCCACCACGGTTATACTGATTCTTCTCTACT	ssDNA	
AC-982	TGTATAACCGTGGTGGACTTCCCGCCGAAATCAAACGGCGGGAAGTCAACCACGGTTA	ssDNA	Generation of *35S:shc-U51G-NbSu*
AC-983	AATGTAACCGTGGTTGACTTCCCGCCGTTTGATTTCGGCGGGAAGTCCACCACGGTTA	ssDNA	
AC-1077	TGTAAGTAGAGAAGAATCGGTATAACCGTGGTGGACTTCCCGCCGAAATCAAACTGCGGGAAGTCAACCACGGTTACATCGGCTCTTCTTACT	ssDNA	Generation of *35S:shc-U15G/U76C-NbSu*
AC-1078	AATGAGTAAGAAGAGCCGATGTAACCGTGGTTGACTTCCCGCAGTTTGATTTCGGCGGGAAGTCCACCACGGTTATACCGATTCTTCTCTACT	ssDNA	
AC-1085	TGTAAGTAGAGAAGAATCGGTATAAACCGCGGGTTCCTAACAGCGAAATCAAACTCTGTTAGGAAACCGCGGTTTACATCGGCTCTTCTTACT	ssDNA	Generation of *35S:shc-U15G/U76C-NbDXS*
AC-1086	AATGAGTAAGAAGAGCCGATGTAAACCGCGGTTTCCTAACAGAGTTTGATTTCGCTGTTAGGAACCCGCGGTTTATACCGATTCTTCTCTACT	ssDNA	
AC-1087	TGTAAGTAGAGAAGAGTCTGTGTAAACCGCGGGTTCCTAACAGCGAAATCAAACTCTGTTAGGAAACCGCGGTTTACATTGGCTCTTCTTACT	ssDNA	Generation of *35S:shc-A12G/A18G-NbDXS*
AC-1088	AATGAGTAAGAAGAGCCAATGTAAACCGCGGTTTCCTAACAGAGTTTGATTTCGCTGTTAGGAACCCGCGGTTTACACAGACTCTTCTCTACT	ssDNA	
AC-1114	TGTATAACCGTGGTGGACTTCCCGCGGAAATCAAACCGCGGGAAGTCAACCACGGTTA	ssDNA	Generation of *35S:shc-C40G/U51C-NbSu*
AC-1115	AATGTAACCGTGGTTGACTTCCCGCGGTTTGATTTCCGCGGGAAGTCCACCACGGTTA	ssDNA	
AC-1116	TGTATAACCGTGGTGGACTTCCCGCAGAAATCAAACTGCGGGAAGTCAACCACGGTTA	ssDNA	Generation of *35S:shc-C40A-NbSu*
AC-1117	AATGTAACCGTGGTTGACTTCCCGCAGTTTGATTTCTGCGGGAAGTCCACCACGGTTA	ssDNA	
AC-1118	TGTATAACCGTGGTGGACTTCCCGCTGAAATCAAACAGCGGGAAGTCAACCACGGTTA	ssDNA	Generation of *35S:shc-C40U/U51A-NbSu*
AC-1119	AATGTAACCGTGGTTGACTTCCCGCTGTTTGATTTCAGCGGGAAGTCCACCACGGTTA	ssDNA	
AC-1120	TGTATAACCGTGGTGGACTTCCCGCTGAAATCAAACGGCGGGAAGTCAACCACGGTTA	ssDNA	Generation of *35S:shc-C40U/U51G-NbSu*
AC-1121	AATGTAACCGTGGTTGACTTCCCGCCGTTTGATTTCAGCGGGAAGTCCACCACGGTTA	ssDNA	
AC-1122	TGTATAACCGTGGTGGACTTCCCGCGGAAATCAAACTGCGGGAAGTCAACCACGGTTA	ssDNA	Generation of *35S:shc-C40G-NbSu*
AC-1123	AATGTAACCGTGGTTGACTTCCCGCAGTTTGATTTCCGCGGGAAGTCCACCACGGTTA	ssDNA	
AC-1124	TGTATAAACCGCGGGTTCCTAACAGGGAAATCAAACCCTGTTAGGAAACCGCGGTTTA	ssDNA	Generation of *35S:shc-C40G/U51C-NbDXS*
AC-1125	AATGTAAACCGCGGTTTCCTAACAGGGTTTGATTTCCCTGTTAGGAACCCGCGGTTTA	ssDNA	
AC-1126	TGTATAAACCGCGGGTTCCTAACAGAGAAATCAAACTCTGTTAGGAAACCGCGGTTTA	ssDNA	Generation of *35S:shc-C40A-NbDXS*
AC-1127	AATGTAAACCGCGGTTTCCTAACAGAGTTTGATTTCTCTGTTAGGAACCCGCGGTTTA	ssDNA	
AC-1128	TGTATAAACCGCGGGTTCCTAACAGTGAAATCAAACACTGTTAGGAAACCGCGGTTTA	ssDNA	Generation of *35S:shc-C40U/U51A-NbDXS*
AC-1129	AATGTAAACCGCGGTTTCCTAACAGTGTTTGATTTCACTGTTAGGAACCCGCGGTTTA	ssDNA	
AC-1130	TGTATAAACCGCGGGTTCCTAACAGTGAAATCAAACGCTGTTAGGAAACCGCGGTTTA	ssDNA	Generation of *35S:shc-C40U/U51G-NbDXS*
AC-1131	AATGTAAACCGCGGTTTCCTAACAGCGTTTGATTTCACTGTTAGGAACCCGCGGTTTA	ssDNA	
AC-1132	TGTATAAACCGCGGGTTCCTAACAGGGAAATCAAACTCTGTTAGGAAACCGCGGTTTA	ssDNA	Generation of *35S:shc-C40G-NbDXS*
AC-1133	AATGTAAACCGCGGTTTCCTAACAGAGTTTGATTTCCCTGTTAGGAACCCGCGGTTTA	ssDNA	

AC-1134	TGTAAGTAGAGAAGAATCCGTATAACCGTGGTGGACTTC CCGCCGAAATCAAACTGCGGGAAGTCAACCACGGTTAC ATGGGCTCTTCTTACT	ssDNA	Generation of *35S:shc-U15C/U76G-NbSu*
AC-1135	AATGAGTAAGAAGAGCCCATGTAACCGTGGTTGACTTCC CGCAGTTTGATTTCGGCGGGAAGTCCACCACGGTTATAC GGATTCTTCTCTACT	ssDNA	
AC-1136	TGTAAGTAGAGAAGAATCTGTATAACCGTGGTGGACTTC CCGCCGAAATCAAACTGCGGGAAGTCAACCACGGTTAC ATAGGCTCTTCTTACT	ssDNA	Generation of *35S:shc-U76A-NbSu*
AC-1137	AATGAGTAAGAAGAGCCTATGTAACCGTGGTTGACTTCC CGCAGTTTGATTTCGGCGGGAAGTCCACCACGGTTATAC AGATTCTTCTCTACT	ssDNA	
AC-1138	TGTAAGTAGAGAAGAATCGGTATAACCGTGGTGGACTTC CCGCCGAAATCAAACTGCGGGAAGTCAACCACGGTTAC ATTGGCTCTTCTTACT	ssDNA	Generation of *35S:shc-U15G-NbSu*
AC-1139	AATGAGTAAGAAGAGCCAATGTAACCGTGGTTGACTTCC CGCAGTTTGATTTCGGCGGGAAGTCCACCACGGTTATAC CGATTCTTCTCTACT	ssDNA	
AC-1140	TGTAAGTAGAGAAGAATCTGTATAACCGTGGTGGACTTC CCGCCGAAATCAAACTGCGGGAAGTCAACCACGGTTAC ATGGGCTCTTCTTACT	ssDNA	Generation of *35S:shc-U76G-NbSu*
AC-1141	AATGAGTAAGAAGAGCCCATGTAACCGTGGTTGACTTCC CGCAGTTTGATTTCGGCGGGAAGTCCACCACGGTTATAC AGATTCTTCTCTACT	ssDNA	
AC-1142	TGTAAGTAGAGAAGAATCCGTATAAACCGCGGGTTCCTA ACAGCGAAATCAAACTCTGTTAGGAAACCGCGGTTTACA TGGGCTCTTCTTACT	ssDNA	Generation of *35S:shc-U15C/U76G-NbDXS*
AC-1143	AATGAGTAAGAAGAGCCCATGTAAACCGCGGTTTCCTA ACAGAGTTTGATTTCGCTGTTAGGAACCCGCGGTTTATA CGGATTCTTCTCTACT	ssDNA	
AC-1144	TGTAAGTAGAGAAGAATCTGTATAAACCGCGGGTTCCTA ACAGCGAAATCAAACTCTGTTAGGAAACCGCGGTTTACA TAGGCTCTTCTTACT	ssDNA	Generation of *35S:shc-U76A-NbDXS*
AC-1145	AATGAGTAAGAAGAGCCTATGTAAACCGCGGTTTCCTAA CAGAGTTTGATTTCGCTGTTAGGAACCCGCGGTTTATAC AGATTCTTCTCTACT	ssDNA	
AC-1146	TGTAAGTAGAGAAGAATCGGTATAAACCGCGGGTTCCT AACAGCGAAATCAAACTCTGTTAGGAAACCGCGGTTTAC ATTGGCTCTTCTTACT	ssDNA	Generation of *35S:shc-U15G-NbDXS*
AC-1147	AATGAGTAAGAAGAGCCAATGTAAACCGCGGTTTCCTA ACAGAGTTTGATTTCGCTGTTAGGAACCCGCGGTTTATA CCGATTCTTCTCTACT	ssDNA	
AC-1148	TGTAAGTAGAGAAGAATCTGTATAAACCGCGGGTTCCTA ACAGCGAAATCAAACTCTGTTAGGAAACCGCGGTTTACA TGGGCTCTTCTTACT	ssDNA	Generation of *35S:shc-U76G-NbDXS*
AC-1149	AATGAGTAAGAAGAGCCCATGTAAACCGCGGTTTCCTA ACAGAGTTTGATTTCGCTGTTAGGAACCCGCGGTTTATA CAGATTCTTCTCTACT	ssDNA	
AC-1150	TGTAAGTAGAGAAGACTCTGTATAACCGTGGTGGACTTC CCGCCGAAATCAAACTGCGGGAAGTCAACCACGGTTAC ATTGGGTCTTCTTACT	ssDNA	Generation of *35S:shc-A12C/C79G-NbSu*
AC-1151	AATGAGTAAGAAGACCCAATGTAACCGTGGTTGACTTCC CGCAGTTTGATTTCGGCGGGAAGTCCACCACGGTTATAC AGAGTCTTCTCTACT	ssDNA	
AC-1152	TGTAAGTAGAGAAGAATCTGTATAACCGTGGTGGACTTC CCGCCGAAATCAAACTGCGGGAAGTCAACCACGGTTAC ATTGGTTCTTCTTACT	ssDNA	Generation of *35S:shc-C79U-NbSu*
AC-1153	AATGAGTAAGAAGAACCAATGTAACCGTGGTTGACTTCC CGCAGTTTGATTTCGGCGGGAAGTCCACCACGGTTATAC AGATTCTTCTCTACT	ssDNA	
AC-1154	TGTAAGTAGAGAAGATTCTGTATAACCGTGGTGGACTTC CCGCCGAAATCAAACTGCGGGAAGTCAACCACGGTTAC ATTGGATCTTCTTACT	ssDNA	Generation of *35S:shc-A12U/C79A-NbSu*
AC-1155	AATGAGTAAGAAGATCCAATGTAACCGTGGTTGACTTCC CGCAGTTTGATTTCGGCGGGAAGTCCACCACGGTTATAC AGAATCTTCTCTACT	ssDNA	

AC-1156	TGTAAGTAGAGAAGAGTCTGTATAACCGTGGTGGACTTCCCGCCGAAATCAAACTGCGGGAAGTCAACCACGGTTACATTGGTTCTTCTTACT	ssDNA	Generation of *35S:shc-A12G/C79U-NbSu*
AC-1157	AATGAGTAAGAAGAACCAATGTAACCGTGGTTGACTTCCCGCAGTTTGATTTCGGCGGGAAGTCCACCACGGTTATACAGACTCTTCTCTACT	ssDNA	
AC-1158	TGTAAGTAGAGAAGATTCTGTATAACCGTGGTGGACTTCCCGCCGAAATCAAACTGCGGGAAGTCAACCACGGTTACATTGGGTCTTCTTACT	ssDNA	Generation of *35S:shc-A12U/C79G-NbSu*
AC-1159	AATGAGTAAGAAGACCCAATGTAACCGTGGTTGACTTCCCGCAGTTTGATTTCGGCGGGAAGTCCACCACGGTTATACAGAATCTTCTCTACT	ssDNA	
AC-1160	TGTAAGTAGAGAAGACTCTGTATAAACCGCGGGTTCCTAACAGCGAAATCAAACTCTGTTAGGAAACCGCGGTTTACATTGGGTCTTCTTACT	ssDNA	Generation of *35S:shc-A12C/C79G*
AC-1161	AATGAGTAAGAAGACCCAATGTAAACCGCGGTTTCCTAACAGAGTTTGATTTCGCTGTTAGGAACCCGCGGTTTATACAGAGTCTTCTCTACT	ssDNA	
AC-1162	TGTAAGTAGAGAAGAATCTGTATAAACCGCGGGTTCCTAACAGCGAAATCAAACTCTGTTAGGAAACCGCGGTTTACATTGGTTCTTCTTACT	ssDNA	Generation of *35S:shc-C79U-NbDXS*
AC-1163	AATGAGTAAGAAGAACCAATGTAAACCGCGGTTTCCTAACAGAGTTTGATTTCGCTGTTAGGAACCCGCGGTTTATACAGATTCTTCTCTACT	ssDNA	
AC-1164	TGTAAGTAGAGAAGATTCTGTATAAACCGCGGGTTCCTAACAGCGAAATCAAACTCTGTTAGGAAACCGCGGTTTACATTGGATCTTCTTACT	ssDNA	Generation of *35S:shc-A12U/C79A-NbDXS*
AC-1165	AATGAGTAAGAAGATCCAATGTAAACCGCGGTTTCCTAACAGAGTTTGATTTCGCTGTTAGGAACCCGCGGTTTATACAGAATCTTCTCTACT	ssDNA	
AC-1166	TGTAAGTAGAGAAGAGTCTGTATAAACCGCGGGTTCCTAACAGCGAAATCAAACTCTGTTAGGAAACCGCGGTTTACATTGGTTCTTCTTACT	ssDNA	Generation of *35S:shc-A12G/C79U-NbDXS*
AC-1167	AATGAGTAAGAAGAACCAATGTAAACCGCGGTTTCCTAACAGAGTTTGATTTCGCTGTTAGGAACCCGCGGTTTATACAGACTCTTCTCTACT	ssDNA	
AC-1168	TGTAAGTAGAGAAGATTCTGTATAAACCGCGGGTTCCTAACAGCGAAATCAAACTCTGTTAGGAAACCGCGGTTTACATTGGGTCTTCTTACT	ssDNA	Generation of *35S:shc-A12U/C79G-NbDXS*
AC-1169	AATGAGTAAGAAGACCCAATGTAAACCGCGGTTTCCTAACAGAGTTTGATTTCGCTGTTAGGAACCCGCGGTTTATACAGAATCTTCTCTACT	ssDNA	
AC-1180	TGTATGCGCTTGCTGAGTTTCCCCCCGAAATCAAACTGGGGGAAACTAAGCAAGCGCA	ssDNA	Generation of *35S:shc-GUS*$_{At}$
AC-1181	AATGTGCGCTTGCTTAGTTTCCCCCAGTTTGATTTCGGGGGGAAACTCAGCAAGCGCA	ssDNA	
AC-1237	TGTAAGTAGAGAAGAGTCGGTATAAACCGCGGGTTCCTAACAGCGAAATCAAACTCTGTTAGGAAACCGCGGTTTACATCGGCTCTTCTTACT	ssDNA	Generation of *35S:shc-A12G/U15G-NbDXS*
AC-1238	AATGAGTAAGAAGAGCCGATGTAAACCGCGGTTTCCTAACAGAGTTTGATTTCGCTGTTAGGAACCCGCGGTTTATACCGACTCTTCTCTACT	ssDNA	
AC-1239	TGTAAGTAGAGAAGAATCGGTGTAAACCGCGGGTTCCTAACAGCGAAATCAAACTCTGTTAGGAAACCGCGGTTTACATCGGCTCTTCTTACT	ssDNA	Generation of *35S:shc-U15G/A18G-NbDXS*
AC-1240	AATGAGTAAGAAGAGCCGATGTAAACCGCGGTTTCCTAACAGAGTTTGATTTCGCTGTTAGGAACCCGCGGTTTACACCGATTCTTCTCTACT	ssDNA	
AC-1241	TGTAAGTAGAGAAGAGTCGGTGTAAACCGCGGGTTCCTAACAGCGAAATCAAACTCTGTTAGGAAACCGCGGTTTACATCGGCTCTTCTTACT	ssDNA	Generation of *35S:shc-A12G/U15G/A18G-NbDXS*
AC-1242	AATGAGTAAGAAGAGCCGATGTAAACCGCGGTTTCCTAACAGAGTTTGATTTCGCTGTTAGGAACCCGCGGTTTACACCGACTCTTCTCTACT	ssDNA	
AC-1268	CACCAGTAGAGAAGAATCTGTGAGAGACCggtctcAcattggctcttcttact	ssDNA	Generation of *pENTR-BS-AtMIR390a-A18G-*

AC-1269	agtaagaagagccaatgTgagaccGGTCTCTCACAGATTCTTCTCTACTGGTG	ssDNA	BB and pMDC32B-*BS-AtMIR390a-A18G-BB*
AC-1270	CACCTGTGAGAGACCATTAGGCACCCC	ssDNA	Generation of *pENTR-BS-AtMIR390a-A18G-B/c* and *pMDC32B-BS-AtMIR390a-A18G-B/c*
AC-1271	aatgTgagaccGTCGAGGTGC	ssDNA	
AC-1272	TGTAAAGCTCAGGAGGGATAGCGCCATGATGATCACATTCGTTATCTATTTTTTGGCGCTATCCATCCTGAGTTT	ssDNA	Generation of *35S:AtMIR390a*
AC-1273	AATGAAACTCAGGATGGATAGCGCCAAAAAATAGATAACGAATGTGATCATCATGGCGCTATCCCTCCTGAGCTT	ssDNA	
AC-1274	TGTAAGTAGAGAAGAATCTGTGAAGCTCAGGAGGGATAGCGCCATGATGATCACATTCGTTATCTATTTTTTGGCGCTATCCATCCTGAGTTTCATTGGCTCTTCTTACT	ssDNA	Generation of *35S:AtMIR390a-A18G*
AC-1275	AATGAGTAAGAAGAGCCAATGAAACTCAGGATGGATAGCGCCAAAAAATAGATAACGAATGTGATCATCATGGCGCTATCCCTCCTGAGCTTCACAGATTCTTCTCTACT	ssDNA	
AC-1276	TGTGTTGGTTATAAAGGAAGAGGCCCGAAATCAAACTGGCCTCTTCCGTTATAACCAA	ssDNA	Generation of *35S:shc-A18G-AtFT*
AC-1277	AATGTTGGTTATAACGGAAGAGGCCAGTTTGATTTCGGGCCTCTTCCTTTATAACCAA	ssDNA	
AC-1278	TGTGTTAAGTGTCACGGAAATCCCTCGAAATCAAACTAGGGATTTCCTTGACACTTAA	ssDNA	Generation of *35S:shc-A18G-AtCH42*
AC-1279	AATGTTAAGTGTCAAGGAAATCCCTAGTTTGATTTCGAGGGATTTCCGTGACACTTAA	ssDNA	
AC-1280	TGTATTCGCCTTGACCTGATCCCTTCGAAATCAAACTAAGGGATCAGTTCAAGGCGAA	ssDNA	Generation of *35S:shc-AtELF3*
AC-1281	AATGTTCGCCTTGAACTGATCCCTTAGTTTGATTTCGAAGGGATCAGGTCAAGGCGAA	ssDNA	
AC-1282	TGTGTTCGCCTTGACCTGATCCCTTCGAAATCAAACTAAGGGATCAGTTCAAGGCGAA	ssDNA	Generation of *35S:shc-A18G-AtELF3*
AC-1286	TGTAAGTAGAGAAGAATCTGTGTAACCGTGGTGGACTTCCCGCATGATGATCACATTCGTTATCTATTTTTTGCGGGAAGTCAACCACGGTTACATTGGCTCTTCTTACT	ssDNA	Generation of *35S:AtMIR390a-A18G-NbSu*
AC-1287	AATGAGTAAGAAGAGCCAATGTAACCGTGGTTGACTTCCCGCAAAAAATAGATAACGAATGTGATCATCATGCGGGAAGTCCACCACGGTTACACAGATTCTTCTCTACT	ssDNA	
AC-1288	TGTAAGTAGAGAAGAATCTGTGTAAACCGCGGGTTCCTAACAGATGATGATCACATTCGTTATCTATTTTTTCTGTTAGGAAACCGCGGTTTACATTGGCTCTTCTTACT	ssDNA	Generation of *35S:AtMIR390a-A18G-NbDXS*
AC-1289	AATGAGTAAGAAGAGCCAATGTAAACCGCGGTTTCCTAACAGAAAAAATAGATAACGAATGTGATCATCATCTGTTAGGAACCCGCGGTTTACACAGATTCTTCTCTACT	ssDNA	

*ssDNA: single-stranded DNA; dsDNA: double-stranded DNA; LNA: locked nucleic acid.

Text S1

Protocol to design and clone amiRNAs downstream the BS region in *BS-AtMIR390a-A18G-Bsa*I/*ccd*B-based ('B/c') vectors.

1. Selection of the amiRNA sequence

Use the amiRNA Designer tool from the P-SAMS script at https://github.com/carringtonlab/p-sams.

2. Design of amiRNA oligonucleotides

Use amiRNA Designer tool from the P-SAMS script at https://github.com/carringtonlab/p-sams.

2.2.1 Sequence of the *BS-AtMIR390a-A18G* cassette containing the amiRNA

The following FASTA sequence includes amiRNA/amiRNA* sequences inserted in the *BS-AtMIR390a-A18G* precursor sequence downstream the BS region:

>amiRNA in *BS-AtMIR390a-A18G*

AGTAGAGAAGAATCTTGTGG$X_1X_2X_3X_4X_5X_6X_7X_8X_9X_{10}X_{11}X_{12}X_{13}X_{14}X_{15}X_{16}X_{17}X_{18}X_{19}X_{20}X_{21}$CGAAATCAAACT$X_1X_2X_1X_2X_3X_4X_5X_6X_7X_8X_9X_{10}X_{11}X_{12}X_{13}X_{14}X_{15}X_{16}X_{17}X_{18}X_{19}$**CATTGGCTCTTCTTACT**

Where:

- -X is a DNA base of the amiRNA sequence, and the subscript number is the base position in the amiRNA 21-mer
- -X is a DNA base of the amiRNA* sequence, and the subscript number is the base position in the amiRNA* 21-mer
- -**X** is a DNA base of the BS region of the *AtMIR390a* precursor
- -X is a DNA base of the *OsMIR390* precursor included in the oligonucleotides required to clone the amiRNA insert in B/c vectors
- -G is A18G modification
- -**X** is a DNA base of the *AtMIR390a* precursor included in the oligonucleotides required to clone the amiRNA insert in B/c vectors
- -X is a DNA base of the *OsMIR390a* precursor that may be modified to preserve the authentic *AtMIR390a* duplex structure

In the sequence above:

-Insert the amiRNA sequence where you see

$X_1X_2X_3X_4X_5X_6X_7X_8X_9X_{10}X_{11}X_{12}X_{13}X_{14}X_{15}X_{16}X_{17}X_{18}X_{19}X_{20}X_{21}$

-Insert the amiRNA* sequence that has to verify the following base-pairing:

$X_1\ X_2\ X_3\ X_4\ X_5\ X_6\ X_7\ X_8\ X_9\ X_{10}X_{11}X_{12}X_{13}X_{14}X_{15}X_{16}X_{17}X_{18}\ X_{19}\ X_{20}X_{21}$

| | | | | | | | | | | | | | | | | | | |

$X_{19}X_{18}X_{17}X_{16}X_{15}X_{14}X_{13}X_{12}X_{11}X_{10}X_9\ X_8\ X_7\ X_6\ X_5\ X_4\ X_3\ X_2\ X_1\ X_2\ X_1$

Note that:

-In general, X_1=T for amiRNA association with AGO1. In this case, X_{19}=A

-Bases X_{11} and X_9 DO NOT base-pair to preserve the central bulge of the authentic *AtMIR390a* duplex. The following base-pair rule applies:

-If X_{11}=G, then X_9=A

-If X_{11}=C, then X_9=T

-If X_{11}=A, then X_9=G

-If X_{11}=U, then X_9=C

2.2.2. Sequence of the amiRNA oligonucleotides

The sequences of the two amiRNA oligonucleotides are:

-Forward oligonucleotide (58 b),

TGTG$X_1X_2X_3X_4X_5X_6X_7X_8X_9X_{10}X_{11}X_{12}X_{13}X_{14}X_{15}X_{16}X_{17}X_{18}X_{19}X_{20}X_{21}$CGAAATCAAACT$X_1X_2X_1X_2X_3X_4$
$X_5X_6X_7X_8X_9X_{10}X_{11}X_{12}X_{13}X_{14}X_{15}X_{16}X_{17}X_{18}X_{19}$

-Reverse oligonucleotide (58 b),

AATG$Y_{19}Y_{18}Y_{17}Y_{16}Y_{15}Y_{14}Y_{13}Y_{12}Y_{11}Y_{10}Y_9Y_8Y_7Y_6Y_5Y_4Y_3Y_2Y_1Y_2Y_1$AGTTTGATTTCG$Y_{21}Y_{20}Y_{19}Y_{18}Y_{17}$
$Y_{16}Y_{15}Y_{14}Y_{13}Y_{12}Y_{11}Y_{10}Y_9Y_8Y_7Y_6Y_5Y_4Y_3Y_2Y_1$

Where:

-$X_1X_2X_3X_4X_5X_6X_7X_8X_9X_{10}X_{11}X_{12}X_{13}X_{14}X_{15}X_{16}X_{17}X_{18}X_{19}X_{20}X_{21}$=amiRNA sequence

-$X_1X_2X_3X_4X_5X_6X_7X_8X_9X_{10}X_{11}X_{12}X_{13}X_{14}X_{15}X_{16}X_{17}X_{18}X_{19}$=partial amiRNA* sequence

-$Y_{21}Y_{20}Y_{19}Y_{18}Y_{17}Y_{16}Y_{15}Y_{14}Y_{13}Y_{12}Y_{11}Y_{10}Y_9Y_8Y_7Y_6Y_5Y_4Y_3Y_2Y_1$=amiRNA reverse-complement sequence

-TG$Y_{19}Y_{18}Y_{17}Y_{16}Y_{15}Y_{14}Y_{13}Y_{12}Y_{11}Y_{10}Y_9Y_8Y_7Y_6Y_5Y_4Y_3Y_2Y_1$=amiRNA* reverse-complement sequence

-X_1X_2 = *OsMIR390* sequence that may be modified to preserve authentic *OsMIR390a* duplex structure.

-Y_2Y_1 = reverse-complement of X_1X_2

Example:

The sequences of the two oligonucleotides to clone the amiRNA 'amiR-NbSu' (TCCCATTCGATACTGCTCGCC) are:

-Sense oligonucleotide (58 b),

TGTGTAACCGTGGTGGACTTCCCGCCGAAATCAAACTGC**GGGAAGTCAACCACGGTTA**

-Antisense oligonucleotide (58 b),

AATGTAACCGTGGTTGACTTCCCGCAGTTTGATTTCG**GCGGGAAGTCCACCACGGTTA**

Note: *the 58 b long oligonucleotides can be ordered desalted, no purification is required.*

3. Cloning of amiRNA sequence(s) in *BS-AtMIR390a-A18G-B/c*-based vectors

Notes:

-Available BS-AtMIR390a-A18G-B/c vectors are listed in Table I at the end of the section.

-BS-AtMIR390a-A18G-B/c-based vectors must be propagated in a ccd*B resistant* E. coli *strain such as DB3.1.*

-Alternatively, Bsa*I digestion of the B/c vector and subsequent ligation of the amiRNA oligonucleotide insert can be done in separate reactions*

3.1. Oligonucleotide annealing

-Dilute sense oligonucleotide and antisense oligonucleotide in sterile H_2O to a final concentration of 100 µM.

-Prepare Oligo Annealing Buffer:

60 mM Tris-HCl (pH 7.5)

500 mM NaCl

60 mM $MgCl_2$

10 mM DTT

Note: *Prepare 1 ml aliquots of Oligo Annealing Buffer and store at -20^{0}C.*

-Assemble the annealing reaction in a PCR tube as described below:

Forward oligonucleotide (100 µM)	2 µL
Reverse oligonucleotide (100 µM)	2 µL
Oligo Annealing Buffer	46 µL

Total volume	50 µL

The final concentration of each oligonucleotide is 4 µM.

-Use a thermocycler to heat the annealing reaction 5 min at 94°C and then cool down (0.05ºC/sec) to 20°C.

-Dilute the annealed oligonucleotides just prior to assembling the digestion-ligation reaction as described below:

Annealed oligonucleotides	3 µL
dH_2O	37 µL
Total volume	40 µL

The final concentration of each oligonucleotide is 0.15 µM.

***Note**: Do not store the diluted oligonucleotides.*

3.2. Digestion-ligation reaction

- Assemble the digestion-ligation reaction as described below:

B/c vector (x ug/uL)	Y µL (50 ng)
Diluted annealed oligonucleotides	1 µL
10x T4 DNA ligase buffer	1 µL
T4 DNA ligase (400 U/µL)	1 µL
*Bsa*I (10U/ µL, NEB)	1 µL
dH_2O	to 10 µL
Total volume	10 µL

Prepare a negative control reaction lacking *Bsa*I.

-Mix the reactions by pipetting. Incubate the reactions at room temperature for 5 minutes at 37°C.

3.3. *E. coli* transformation and analysis of transformants

-Transform 1-5 ul of the digestion-ligation reaction into an *E. coli* strain that doesn't have *ccd*B resistance (e.g. DH10B, TOP10, …) to do counter-selection.

-Pick two colonies/construct, grow LB-Kan (100 mg/ml) cultures and purify plasmids.

-Sequence with appropriate primers: M13-F (CCCAGTCACGACGTTGTAAAACGACGG) and M13-R (CAGAGCTGCCAGGAAACAGCTATGACC) for *pENTR*-based vectors; attB1 (ACAAGTTTGTACAAAAAAGCAGGCT) and attB2 (ACCACTTTGTACAAGAAAGCTGGGT) primers for *pMDC32B*-based vectors).

Table I: *Bsa*I/*ccd*B-based ('B/c') vectors for direct cloning of amiRNAs downstream the BS region in *AtMIR390a-A18G* precursor.

Vector	Small RNA expressed	Bacterial antibiotic resistance	Plant antibiotic resistance	GATEWAY use	Backbone	Promoter of syn-tasiRNA cassette	Terminator of syn-tasiRNA cassette	Plant species tested
pENTR-BS-AtMIR390a-A18G-B/c	–	Kanamycin	–	Donor	*pENTR*	–	–	–
pMDC32B-BS-AtMIR390a-A18GB/c	amiRNA	Kanamycin<br>Hygromycin	Hygromycin	–	*pMDC32*	*CaMV* 2x35S	*Nos*	*A. thaliana*<br>*N. benthamiana*

Text S2.

FASTA sequences of miRNA/amiRNA-producing precursors.

AtMIR390a
OsMIR390
miRNA/amiRNA
miRNA*/amiRNA*
N and N: mutations tested
N is a DNA base of the *OsMIR390a* precursor that may be modified to preserve the authentic *AtMIR390a* duplex structure

>AtMIR390a
AGTAGAGAAGAATCTGTAAAGCTCAGGAGGGATAGCGCCATGATGATCACATTCGTTATCTATTTTTTGGCGCTA
TCCATCCTGAGTTTCATTGGCTCTTCTTACT

>AtMIR390a-A18G
AGTAGAGAAGAATCTGTGAAGCTCAGGAGGGATAGCGCCATGATGATCACATTCGTTATCTATTTTTTGGCGCTA
TCCATCCTGAGTTTCATTGGCTCTTCTTACT

>AtMIR390a-GUS$_{Nb}$
AGTAGAGAAGAATCTGTATCTTGTAACGCGCTTTCCCAGATGATGATCACATTCGTTATCTATTTTTTCTGGGAA
AGCTCGTTACAAGACATTGGCTCTTCTTACT

>AtMIR390a-NbSu
AGTAGAGAAGAATCTGTATAACCGTGGTGGACTTCCCGCATGATGATCACATTCGTTATCTATTTTTTGCGGGAA
GTCAACCACGGTTACATTGGCTCTTCTTACT

>AtMIR390a-A18G-NbSu
AGTAGAGAAGAATCTGTGTAACCGTGGTGGACTTCCCGCATGATGATCACATTCGTTATCTATTTTTTGCGGGAA
GTCAACCACGGTTACATTGGCTCTTCTTACT

>AtMIR390a-NbDXS
AGTAGAGAAGAATCTGTATAAACCGCGGGTTCCTAACAGATGATGATCACATTCGTTATCTATTTTTTCTGTTAG
GAAACCGCGGTTTACATTGGCTCTTCTTACT

>AtMIR390a-A18G-NbDXS
AGTAGAGAAGAATCTGTGTAAACCGCGGGTTCCTAACAGATGATGATCACATTCGTTATCTATTTTTTCTGTTAG
GAAACCGCGGTTTACATTGGCTCTTCTTACT

>shc-GUS$_{Nb}$
AGTAGAGAAGAATCTGTATCTTGTAACGCGCTTTCCCAGCGAAATCAAACTCTGGGAAAGCTCGTTACAAGACAT
TGGCTCTTCTTACT

>shc-NbSu
AGTAGAGAAGAATCTGTATAACCGTGGTGGACTTCCCGCCGAAATCAAACTGCGGGAAGTCAACCACGGTTACAT
TGGCTCTTCTTACT

>shc-A18G-NbSu
AGTAGAGAAGAATCTGTGTAACCGTGGTGGACTTCCCGCCGAAATCAAACTGCGGGAAGTCAACCACGGTTACAT
TGGCTCTTCTTACT

>shc-A18U/C73A-NbSu
AGTAGAGAAGAATCTGTTTAACCGTGGTGGACTTCCCGCCGAAATCAAACTGCGGGAAGTCAACCACGGTTAAAT
TGGCTCTTCTTACT

>shc-A18U/C73G-NbSu

AGTAGAGAAGAATCTGTTTAACCGTGGTGGACTTCCCGCCGAAATCAAACTGCGGGAAGTCAACCACGGTTAGAT
TGGCTCTTCTTACT

>shc-A18G/C73U-NbSu
AGTAGAGAAGAATCTGTGTAACCGTGGTGGACTTCCCGCCGAAATCAAACTGCGGGAAGTCAACCACGGTTATAT
TGGCTCTTCTTACT

>shc-A18C/C73G-NbSu
AGTAGAGAAGAATCTGTCTAACCGTGGTGGACTTCCCGCCGAAATCAAACTGCGGGAAGTCAACCACGGTTAGAT
TGGCTCTTCTTACT

>shc-NbDXS
AGTAGAGAAGAATCTGTATAAACCGCGGGTTCCTAACAGCGAAATCAAACTCTGTTAGGAAACCGCGGTTTACAT
TGGCTCTTCTTACT

>shc-A18G-NbDXS
AGTAGAGAAGAATCTGTGTAAACCGCGGGTTCCTAACAGCGAAATCAAACTCTGTTAGGAAACCGCGGTTTACAT
TGGCTCTTCTTACT

>shc-C73U-NbDXS
AGTAGAGAAGAATCTGTATAAACCGCGGGTTCCTAACAGCGAAATCAAACTCTGTTAGGAAACCGCGGTTTATAT
TGGCTCTTCTTACT

>shc-A18U/C73A-NbDXS
AGTAGAGAAGAATCTGTTTAAACCGCGGGTTCCTAACAGCGAAATCAAACTCTGTTAGGAAACCGCGGTTTAAAT
TGGCTCTTCTTACT

>shc-A18U/C73G-NbDXS
AGTAGAGAAGAATCTGTTTAAACCGCGGGTTCCTAACAGCGAAATCAAACTCTGTTAGGAAACCGCGGTTTAGAT
TGGCTCTTCTTACT

>shc-A18G/C73U-NbDXS
AGTAGAGAAGAATCTGTGTAAACCGCGGGTTCCTAACAGCGAAATCAAACTCTGTTAGGAAACCGCGGTTTATAT
TGGCTCTTCTTACT

>shc-A18C/C73G-NbDXS
AGTAGAGAAGAATCTGTCTAAACCGCGGGTTCCTAACAGCGAAATCAAACTCTGTTAGGAAACCGCGGTTTAGAT
TGGCTCTTCTTACT

>shc-C40G/U51C-NbSu
AGTAGAGAAGAATCTGTATAACCGTGGTGGACTTCCCGCGGAAATCAAACCGCGGGAAGTCAACCACGGTTACAT
TGGCTCTTCTTACT

>shc-U51G-NbSu
AGTAGAGAAGAATCTGTATAACCGTGGTGGACTTCCCGCCGAAATCAAACGGCGGGAAGTCAACCACGGTTACAT
TGGCTCTTCTTACT

>shc-C40A-NbSu
AGTAGAGAAGAATCTGTATAACCGTGGTGGACTTCCCGCAGAAATCAAACTGCGGGAAGTCAACCACGGTTACAT
TGGCTCTTCTTACT

>shc-C40U/U51A-NbSu
AGTAGAGAAGAATCTGTATAACCGTGGTGGACTTCCCGCTGAAATCAAACAGCGGGAAGTCAACCACGGTTACAT
TGGCTCTTCTTACT

>shc-C40U/U51G-NbSu
AGTAGAGAAGAATCTGTATAACCGTGGTGGACTTCCCGCTGAAATCAAACGGCGGGAAGTCAACCACGGTTACAT
TGGCTCTTCTTACT

>shc-C40G-NbSu
AGTAGAGAAGAATCTGTATAACCGTGGTGGACTTCCCGCGGAAATCAAACTGCGGGAAGTCAACCACGGTTACAT
TGGCTCTTCTTACT

>shc-C40G/U51C-NbDXS
AGTAGAGAAGAATCTGTATAAACCGCGGGTTCCTAACAGGGAAATCAAACCCTGTTAGGAAACCGCGGTTTACAT
TGGCTCTTCTTACT

>shc-U51G-NbDXS
AGTAGAGAAGAATCTGTATAAACCGCGGGTTCCTAACAGCGAAATCAAACGCTGTTAGGAAACCGCGGTTTACAT
TGGCTCTTCTTACT

>shc-C40A-NbDXS
AGTAGAGAAGAATCTGTATAAACCGCGGGTTCCTAACAGAGAAATCAAACTCTGTTAGGAAACCGCGGTTTACAT
TGGCTCTTCTTACT

>shc-C40U/U51A-NbDXS
AGTAGAGAAGAATCTGTATAAACCGCGGGTTCCTAACAGTGAAATCAAACACTGTTAGGAAACCGCGGTTTACAT
TGGCTCTTCTTACT

>shc-C40U/U51G-NbDXS
AGTAGAGAAGAATCTGTATAAACCGCGGGTTCCTAACAGTGAAATCAAACGCTGTTAGGAAACCGCGGTTTACAT
TGGCTCTTCTTACT

>shc-C40G-NbDXS
AGTAGAGAAGAATCTGTATAAACCGCGGGTTCCTAACAGGGAAATCAAACTCTGTTAGGAAACCGCGGTTTACAT
TGGCTCTTCTTACT

>shc-A12G-NbSu
AGTAGAGAAGAGTCTGTATAACCGTGGTGGACTTCCCGCCGAAATCAAACTGCGGGAAGTCAACCACGGTTACAT
TGGCTCTTCTTACT

>shc-C79U-NbSu
AGTAGAGAAGAATCTGTATAACCGTGGTGGACTTCCCGCCGAAATCAAACTGCGGGAAGTCAACCACGGTTACAT
TGGTTCTTCTTACT

>shc-A12U/C79A-NbSu
AGTAGAGAAGATTCTGTATAACCGTGGTGGACTTCCCGCCGAAATCAAACTGCGGGAAGTCAACCACGGTTACAT
TGGATCTTCTTACT

>shc-A12U/C79G-NbSu
AGTAGAGAAGATTCTGTATAACCGTGGTGGACTTCCCGCCGAAATCAAACTGCGGGAAGTCAACCACGGTTACAT
TGGGTCTTCTTACT

>shc-A12C/C79G-NbSu
AGTAGAGAAGACTCTGTATAACCGTGGTGGACTTCCCGCCGAAATCAAACTGCGGGAAGTCAACCACGGTTACAT
TGGGTCTTCTTACT

>shc-A12G-C79U-NbSu
AGTAGAGAAGAGTCTGTATAACCGTGGTGGACTTCCCGCCGAAATCAAACTGCGGGAAGTCAACCACGGTTACAT
TGGTTCTTCTTACT

>shc-A12G-NbDXS
AGTAGAGAAGAGTCTGTATAAACCGCGGGTTCCTAACAGCGAAATCAAACTCTGTTAGGAAACCGCGGTTTACAT
TGGCTCTTCTTACT

>shc-C79U-NbDXS

AGTAGAGAAGAATCTGTATAAACCGCGGGTTCCTAACAGCGAAATCAAACTCTGTTAGGAAACCGCGGTTTACAT
TGG**T**TCTTCTTACT

>shc-A12U/C79A-NbDXS
AGTAGAGAAGA**T**TCTGTATAAACCGCGGGTTCCTAACAGCGAAATCAAACTCTGTTAGGAAACCGCGGTTTACAT
TGG**A**TCTTCTTACT

>shc-A12U/C79G-NbDXS
AGTAGAGAAGA**T**TCTGTATAAACCGCGGGTTCCTAACAGCGAAATCAAACTCTGTTAGGAAACCGCGGTTTACAT
TGG**G**TCTTCTTACT

>shc-A12C/C79G-NbDXS
AGTAGAGAAGA**C**TCTGTATAAACCGCGGGTTCCTAACAGCGAAATCAAACTCTGTTAGGAAACCGCGGTTTACAT
TGG**G**TCTTCTTACT

>shc-A12G-C79U-NbDXS
AGTAGAGAAGA**G**TCTGTATAAACCGCGGGTTCCTAACAGCGAAATCAAACTCTGTTAGGAAACCGCGGTTTACAT
TGG**T**TCTTCTTACT

>shc-U15G/U76C-NbSu
AGTAGAGAAGAATC**G**GTATAACCGTGGTGGACTTCCCGCCGAAATCAAACTGCGGGAAGTCAACCACGGTTACAT
CGGCTCTTCTTACT

>shc-U15C/U76G-NbSu
AGTAGAGAAGAATC**C**GTATAACCGTGGTGGACTTCCCGCCGAAATCAAACTGCGGGAAGTCAACCACGGTTACAT
GGGCTCTTCTTACT

>shc-U15A-NbSu
AGTAGAGAAGAATC**A**GTATAACCGTGGTGGACTTCCCGCCGAAATCAAACTGCGGGAAGTCAACCACGGTTACAT
TGGCTCTTCTTACT

>shc-U76A-NbSu
AGTAGAGAAGAATCTGTATAACCGTGGTGGACTTCCCGCCGAAATCAAACTGCGGGAAGTCAACCACGGTTACAT
AGGCTCTTCTTACT

>shc-U76G-NbSu
AGTAGAGAAGAATCTGTATAACCGTGGTGGACTTCCCGCCGAAATCAAACTGCGGGAAGTCAACCACGGTTACAT
GGGCTCTTCTTACT

>shc-U15G-NbSu
AGTAGAGAAGAATCTGTATAACCGTGGTGGACTTCCCGCCGAAATCAAAC**G**GCGGGAAGTCAACCACGGTTACAT
TGGCTCTTCTTACT

>shc-U15G/U76C-NbDXS
AGTAGAGAAGAATC**G**GTATAAACCGCGGGTTCCTAACAGCGAAATCAAACTCTGTTAGGAAACCGCGGTTTACAT
CGGCTCTTCTTACT

>shc-U15C/U76G-NbDXS
AGTAGAGAAGAATC**C**GTATAAACCGCGGGTTCCTAACAGCGAAATCAAACTCTGTTAGGAAACCGCGGTTTACAT
GGGCTCTTCTTACT

>shc-U15A-NbDXS
AGTAGAGAAGAATC**A**GTATAAACCGCGGGTTCCTAACAGCGAAATCAAACTCTGTTAGGAAACCGCGGTTTACAT
TGGCTCTTCTTACT

>shc-U76A-NbDXS
AGTAGAGAAGAATCTGTATAAACCGCGGGTTCCTAACAGCGAAATCAAACTCTGTTAGGAAACCGCGGTTTACAT
AGGCTCTTCTTACT

>shc-U76G-NbDXS
AGTAGAGAAGAATCTGTATAAACCGCGGGTTCCTAACAGCGAAATCAAACTCTGTTAGGAAACCGCGGTTTACAT
GGGCTCTTCTTACT

>shc-U15G-NbDXS
AGTAGAGAAGAATC**G**GTATAAACCGCGGGTTCCTAACAGCGAAATCAAACTCTGTTAGGAAACCGCGGTTTACAT
TGGCTCTTCTTACT

>shc-A12G-U15G-NbDXS
AGTAGAGAAGA**G**TC**G**GTATAAACCGCGGGTTCCTAACAGCGAAATCAAACTCTGTTAGGAAACCGCGGTTTACAT
CGGCTCTTCTTACT

>shc-A12G/A18G-NbDXS
AGTAGAGAAGA**G**TCTGT**G**TAAACCGCGGGTTCCTAACAGCGAAATCAAACTCTGTTAGGAAACCGCGGTTTACAT
TGGCTCTTCTTACT

>shc-U15G/A18G-NbDXS
AGTAGAGAAGAATC**G**GT**G**TAAACCGCGGGTTCCTAACAGCGAAATCAAACTCTGTTAGGAAACCGCGGTTTACAT
CGGCTCTTCTTACT

>shc-A12G/U15G/A18G-NbDXS
AGTAGAGAAGA**G**TC**G**GT**G**TAAACCGCGGGTTCCTAACAGCGAAATCAAACTCTGTTAGGAAACCGCGGTTTACAT
CGGCTCTTCTTACT

>shc-GUS_{At}
AGTAGAGAAGAATCTGTATGCGCTTGCTGAGTTTCCCCCGAAATCAAACTGGGGGAAACTAAGCAAGCGCACAT
TGGCTCTTCTTACT

>shc-AtCH42
AGTAGAGAAGAATCTGTATTAAGTGTCACGGAAATCCCTCGAAATCAAACTAGGGATTTCCTTGACACTTAACAT
TGGCTCTTCTTACT

>shc-A18G-AtCH42
AGTAGAGAAGAATCTGT**G**TTAAGTGTCACGGAAATCCCTCGAAATCAAACTAGGGATTTCCTTGACACTTAACAT
TGGCTCTTCTTACT

>shc-AtFT
AGTAGAGAAGAATCTGTATTGGTTATAAAGGAAGAGGCCCGAAATCAAACTGGCCTCTTCCGTTATAACCAACAT
TGGCTCTTCTTACT

>shc-A18G-AtFT
AGTAGAGAAGAATCTGT**G**TTCGCCTTGACCTGATCCCTTCGAAATCAAACTAAGGGATCAGTTCAAGGCGAACAT
TGGCTCTTCTTACT

>shc-AtELF3
AGTAGAGAAGAATCTGTATTCGCCTTGACCTGATCCCTTCGAAATCAAACTAAGGGATCAGTTCAAGGCGAACAT
TGGCTCTTCTTACT

>shc-A18G-AtELF3
AGTAGAGAAGAATCTGT**G**TTCGCCTTGACCTGATCCCTTCGAAATCAAACTAAGGGATCAGTTCAAGGCGAACAT
TGGCTCTTCTTACT

Text S3.

DNA sequence of *Bsa*I-*ccd*B-based (B/c) vectors used for direct cloning of amiRNAs in *MIR390*-based *shc* precursors.

>*pENTR-BS-AtMIR390a-A18G-B/c* (4076 bp)
CTTTCCTGCGTTATCCCCTGATTCTGTGGATAACCGTATTACCGCCTTTGAGTGAGCTGATACCGCTCGCCGCAG
CCGAACGACCGAGCGCAGCGAGTCAGTGAGCGAGGAAGCGGAAGAGCGCCCAATACGCAAACCGCCTCTCCCCGC
GCGTTGGCCGATTCATTAATGCAGCTGGCACGACAGGTTTCCCGACTGGAAAGCGGGCAGTGAGCGCAACGCAAT
TAATACGCGTACCGCTAGCCAGGAAGAGTTTGTAGAAACGCAAAAAGGCCATCCGTCAGGATGGCCTTCTGCTTA
GTTTGATGCCTGGCAGTTTATGGCGGGCGTCCTGCCCGCCACCCTCCGGGCCGTTGCTTCACAACGTTCAAATCC
GCTCCCGGCGGATTTGTCCTACTCAGGAGAGCGTTCACCGACAAACAACAGATAAAACGAAAGGCCCAGTCTTCC
GACTGAGCCTTTCGTTTTATTTGATGCCTGGCAGTTCCCTACTCTCGCGTTAACGCTAGCATGGATGTTTTCCCA
GTCACGACGTTGTAAAACGACGGCCAGTCTTAAGCTCGGGCCCCAAATAATGATTTTATTTTGACTGATAGTGAC
CTGTTCGTTGCAACAAATTGATGAGCAATGCTTTTTTATAATGCCAACTTTGTACAAAAAAGCAGGCTCCGCGGC
CGCCCCCTTCACCGTAGAGAAGAATCTGT**G**AGAGACCATTAGGCACCCCAGGCTTTACACTTTATGCTTCCGGCT
CGTATAATGTGTGGATTTTGAGTTAGGAGCCGTCGAGATTTTCAGGAGCTAAGGAAGCTAAAatggagaaaaaaa
tcactggatataccaccgttgatatatcccaatggcatcgtaaagaacattttgaggcatttcagtcagttgctc
aatgtacctataaccagaccgttcagctggatattacggcctttttaaagaccgtaaagaaaaataagcacaagt
tttatccggcctttattcacattcttgcccgcctgatgaatgctcatccggagttccgtatggcaatgaaagacg
gtgagctggtgatatgggatagtgttcacccttgttacaccgttttccatgagcaaactgaaacgttttcatcgc
tctggagtgaataccacgacgatttccggcagtttctacacatatattcgcaagatgtggcgtgttacggtgaaa
acctggcctatttccctaaagggtttattgagaatatgtttttcgtctcagccaatccctgggtgagtttcacca
gttttgatttaaacgtggccaatatggacaacttcttcgcccccgttttcaccatgggcaaatattatacgcaag
gcgacaaggtgctgatgccgctggcgattcaggttcatcatgccgtttgtgatggcttccatgtcggcagaatgc
ttaatgaattacaacagtactgcgatgagtggcagggcggggcgtaaACGCGTGGAGCCGGCTTACTAAAAGCCA
GATAACAGTATGCGTATTTGCGCGCTGATTTTTGCGGTATAAGAATATATACTGATATGTATACCCGAAGTATGT
CAAAAAGAGGTATGCTATGAAGCAGCGTATTACAGTGACAGTTGACAGCGACAGCTATCAGTTGCTCAAGGCATA
TATGATGTCAATATCTCCGGTCTGGTAAGCACAACCATGCAGAATGAAGCCCGTCGTCTGCGTGCCGAACGCTGG
AAAGCGGAAAATCAGGAAGGGATGGCTGAGGTCGCCCGGTTTATTGAAATGAACGGCTCTTTTGCTGACGAGAAC
AGGGGCTGGTGAAATGCAGTTTAAGGTTTACACCTATAAAAGAGAGAGCCGTTATCGTCTGTTTGTGGATGTACA
GAGTGATATTATTGACACGCCCGGCCGACGGATGGTGATCCCCCTGGCCAGTGCACGTCTGCTGTCAGATAAAGT
CTCCCGTGAACTTTACCCGGTGGTGCATATCGGGGATGAAAGCTGGCGCATGATGACCACCGATATGGCCAGTGT
GCCGGTTTCCGTTATCGGGGAAGAAGTGGCTGATCTCAGCCACCGCGAAAATGACATCAAAAACGCCATTAACCT
GATGTTCTGGGGAATATAAATGTCAGGCTCCCTTATACACAGCCAGTCTGCACCTCGACggtctcAcattggctc
ttcttactAAGGGTGGGCGCGCCGACCCAGCTTTCTTGTACAAAGTTGGCATTATAAGAAAGCATTGCTTATCAA
TTTGTTGCAACGAACAGGTCACTATCAGTCAAAATAAAATCATTATTTGCCATCCAGCTGATATCCCCTATAGTG
AGTCGTATTACATGGTCATAGCTGTTTCCTGGCAGCTCTGGCCCGTGTCTCAAAATCTCTGATGTTACATTGCAC
AAGATAAAAATATATCATCATGAACAATAAAACTGTCTGCTTACATAAACAGTAATACAAGGGGTGTTatgagcc
atattcaacgggaaacgtcgaggccgcgattaaattccaacatggatgctgatttatatgggtataaatgggctc
gcgataatgtcgggcaatcaggtgcgacaatctatcgcttgtatgggaagcccgatgcgccagagttgtttctga
aacatggcaaaggtagcgttgccaatgatgttacagatgagatggtcagactaaactggctgacggaatttatgc
ctcttccgaccatcaagcattttatccgtactcctgatgatgcatggttactcaccactgcgatccccggaaaaa
cagcattccaggtattagaagaatatcctgattcaggtgaaaatattgttgatgcgctggcagtgttcctgcgcc
ggttgcattcgattcctgtttgtaattgtccttttaacagcgatcgcgtatttcgtctcgctcaggcgcaatcac
gaatgaataacggtttggttgatgcgagtgattttgatgacgagcgtaatggctggcctgttgaacaagtctgga
aagaaatgcataaactttgccattctcaccggattcagtcgtcactcatggtgatttctcacttgataacctta
tttttgacgaggggaaattaataggttgtattgatgttggacgagtcggaatcgcagaccgataccaggatcttg
ccatcctatggaactgcctcggtgagttttctccttcattacagaaacggctttttcaaaaatatggtattgata
atcctgatatgaataaattgcagtttcatttgatgctcgatgagtttttcTAATCAGAATTGGTTAATTGGTTGT
AACACTGGCAGAGCATTACGCTGACTTGACGGGACGGCGCAAGCTCATGACCAAAATCCCTTAACGTGAGTTACG
CGTCGTTCCACTGAGCGTCAGACCCCGTAGAAAAGATCAAAGGATCTTCTTGAGATCCTTTTTTTCTGCGCGTAA
TCTGCTGCTTGCAAACAAAAAAACCACCGCTACCAGCGGTGGTTTGTTTGCCGGATCAAGAGCTACCAACTCTTT
TTCCGAAGGTAACTGGCTTCAGCAGAGCGCAGATACCAAATACTGTCCTTCTAGTGTAGCCGTAGTTAGGCCACC
ACTTCAAGAACTCTGTAGCACCGCCTACATACCTCGCTCTGCTAATCCTGTTACCAGTGGCTGCTGCCAGTGGCG
ATAAGTCGTGTCTTACCGGGTTGGACTCAAGACGATAGTTACCGGATAAGGCGCAGCGGTCGGGCTGAACGGGGG
GTTCGTGCACACAGCCCAGCTTGGAGCGAACGACCTACACCGAACTGAGATACCTACAGCGTGAGCATTGAGAAA
GCGCCACGCTTCCCGAAGGGAGAAAGGCGGACAGGTATCCGGTAAGCGGCAGGGTCGGAACAGGAGAGCGCACGA
GGGAGCTTCCAGGGGGAAACGCCTGGTATCTTTATAGTCCTGTCGGGTTTCGCCACCTCTGACTTGAGCGTCGAT

```
TTTTGTGATGCTCGTCAGGGGGGCGGAGCCTATGGAAAAACGCCAGCAACGCGGCCTTTTTACGGTTCCTGGCCT
TTTGCTGGCCTTTTGCTCACATGTT
```

PURPLE/UPPERCASE: M13-F binding site
orange/lowercase: attL1
BLUE/UPPERCASE: *AtMIR390a* 5’ region
RED/UPPERCASE/BOLD: A18G mutation
RED/UPPERCASE: *Bsa*I site
magenta/lowercase: chloramphenicol resistance gene
MAGENTA/UPPERCASE: *ccd*B gene
red/lowercase: inverted *Bsa*I site
blue/lowercase: *AtMIR390a* 3' region
orange/lowercase/underlined: attL2
PURPLE/UPPERCASE/UNDERLINED: M13-Reverse binding site
brown/lowercase: Kanamycin resistance gene

>*pMDC32B-BS-AtMIR390-A18G-B/c* (11629 bp)
CCAGCCAGCCAACAGCTCCCCGACCGGCAGCTCGGCACAAAATCACCACTCGATACAGGCAGCCCATCAGTCCGG
GACGGCGTCAGCGGGAGAGCCGTTGTAAGGCGGCAGACTTTGCTCATGTTACCGATGCTATTCGGAAGAACGGCA
ACTAAGCTGCCGGGTTTGAAACACGGATGATCTCGCGGAGGGTAGCATGTTGATTGTAACGATGACAGAGCGTTG
CTGCCTGTGATCACCGCGGTTTCAAAATCGGCTCCGTCGATACTATGTTATACGCCAACTTTGAAAACAACTTTG
AAAAAGCTGTTTTCTGGTATTTAAGGTTTTAGAATGCAAGGAACAGTGAATTGGAGTTCGTCTTGTTATAATTAG
CTTCTTGGGGTATCTTTAAATACTGTAGAAAAGAGGAAGGAAATAATAAatggctaaaatgagaatatcaccgga
attgaaaaaactgatcgaaaaataccgctgcgtaaaagatacggaaggaatgtctcctgctaaggtatataagct
ggtgggagaaaatgaaaacctatatttaaaaatgacggacagccggtataaagggaccacctatgatgtggaacg
ggaaaaggacatgatgctatggctggaaggaaagctgcctgttccaaaggtcctgcactttgaacggcatgatgg
ctggagcaatctgctcatgagtgaggccgatggcgtcctttgctcggaagagtatgaagatgaacaaagccctga
aaagattatcgagctgtatgcggagtgcatcaggctctttcactccatcgacatatcggattgtccctatacgaa
tagcttagacagccgcttagccgaattggattacttactgaataacgatctggccgatgtggattgcgaaaactg
ggaagaagacactccatttaaagatccgcgcgagctgtatgatttttttaaagacggaaaagcccgaagaggaact
tgtcttttcccacggcgacctgggagacagcaacatctttgtgaaagatggcaaagtaagtggctttattgatct
tgggagaagcggcagggcggacaagtggtatgacattgccttctgcgtccggtcgatcagggaggatatcgggga
agaacagtatgtcgagctatttttttgacttactggggatcaagcctgattgggagaaaataaaatattatattt
actggatgaattgttttagTACCTAGAATGCATGACCAAAATCCCTTAACGTGAGTTTTCGTTCCACTGAGCGTC
AGACCCCGTAGAAAAGATCAAAGGATCTTCTTGAGATCCTTTTTTTCTGCGCGTAATCTGCTGCTTGCAAACAAA
AAAACCACCGCTACCAGCGGTGGTTTGTTTGCCGGATCAAGAGCTACCAACTCTTTTTCCGAAGGTAACTGGCTT
CAGCAGAGCGCAGATACCAAATACTGTCCTTCTAGTGTAGCCGTAGTTAGGCCACCACTTCAAGAACTCTGTAGC
ACCGCCTACATACCTCGCTCTGCTAATCCTGTTACCAGTGGCTGCTGCCAGTGGCGATAAGTCGTGTCTTACCGG
GTTGGACTCAAGACGATAGTTACCGGATAAGGCGCAGCGGTCGGGCTGAACGGGGGGTTCGTGCACACAGCCCAG
CTTGGAGCGAACGACCTACACCGAACTGAGATACCTACAGCGTGAGCTATGAGAAAGCGCCACGCTTCCCGAAGG
GAGAAAGGCGGACAGGTATCCGGTAAGCGGCAGGGTCGGAACAGGAGAGCGCACGAGGGAGCTTCCAGGGGGAAA
CGCCTGGTATCTTTATAGTCCTGTCGGGTTTCGCCACCTCTGACTTGAGCGTCGATTTTTGTGATGCTCGTCAGG
GGGGCGGAGCCTATGGAAAAACGCCAGCAACGCGGCCTTTTTACGGTTCCTGGCCTTTTGCTGGCCTTTTGCTCA
CATGTTCTTTCCTGCGTTATCCCCTGATTCTGTGGATAACCGTATTACCGCCTTTGAGTGAGCTGATACCGCTCG
CCGCAGCCGAACGACCGAGCGCAGCGAGTCAGTGAGCGAGGAAGCGGAAGAGCGCCTGATGCGGTATTTTCTCCT
TACGCATCTGTGCGGTATTTCACACCGCATATGGTGCACTCTCAGTACAATCTGCTCTGATGCCGCATAGTTAAG
CCAGTATACACTCCGCTATCGCTACGTGACTGGGTCATGGCTGCGCCCCGACACCCGCCAACACCCGCTGACGCG
CCCTGACGGGCTTGTCTGCTCCCGGCATCCGCTTACAGACAAGCTGTGACCGTCTCCGGGAGCTGCATGTGTCAG
AGGTTTTCACCGTCATCACCGAAACGCGCGAGGCAGGGTGCCTTGATGTGGGCGCCGGCGGTCGAGTGGCGACGG
CGCGGCTTGTCCGCGCCCTGGTAGATTGCCTGGCCGTAGGCCAGCCATTTTTGAGCGGCCAGCGGCCGCGATAGG
CCGACGCGAAGCGGCGGGGCGTAGGGAGCGCAGCGACCGAAGGGTAGGCGCTTTTTGCAGCTCTTCGGCTGTGCG
CTGGCCAGACAGTTATGCACAGGCCAGGCGGGTTTTAAGAGTTTTAATAAGTTTTAAAGAGTTTTAGGCGGAAAA
ATCGCCTTTTTTCTCTTTTATATCAGTCACTTACATGTGTGACCGGTTCCCAATGTACGGCTTTGGGTTCCCAAT
GTACGGGTTCCGGTTCCCAATGTACGGCTTTGGGTTCCCAATGTACGTGCTATCCACAGGAAAGAGAACTTTTCG
ACCTTTTTCCCCTGCTAGGGCAATTTGCCCTAGCATCTGCTCCGTACATTAGGAACCGGCGGATGCTTCGCCCTC
GATCAGGTTGCGGTAGCGCATGACTAGGATCGGGCCAGCCTGCCCCGCCTCCTCCTTCAAATCGTACTCCGGCAG
GTCATTTGACCCGATCAGCTTGCGCACGGTGAAACAGAACTTCTTGAACTCTCCGGCGCTGCCACTGCGTTCGTA
GATCGTCTTGAACAACCATCTGGCTTCTGCCTTGCCTGCGGCGCGGCGTGCCAGGCGGTAGAGAAAACGGCCGAT
GCCGGGATCGATCAAAAAGTAATCGGGGTGAACCGTCAGCACGTCCGGGTTCTTGCCTTCTGTGATCTCGCGGTA
CATCCAATCAGCTAGCTCGATCTCGATGTACTCCGGCCGCCCGGTTTCGCTCTTTACGATCTTGTAGCGGCTAAT
CAAGGCTTCACCCTCGGATACCGTCACCAGGCGGCCGTTCTTGGCCTTCTTCGTACGCTGCATGGCAACGTGCGT
GGTGTTTAACCGAATGCAGGTTTCTACCAGGTCGTCTTTCTGCTTTCCGCCATCGGCTCGCCGGCAGAACTTGAG
TACGTCCGCAACGTGTGGACGGAACACGCGGCCGGGCTTGTCTCCCTTCCCTTCCCGGTATCGGTTCATGGATTC
GGTTAGATGGGAAACCGCCATCAGTACCAGGTCGTAATCCCACACACTGGCCATGCCGGCCGGCCCTGCGGAAAC
CTCTACGTGCCCGTCTGGAAGCTCGTAGCGGATCACCTCGCCAGCTCGTCGGTCACGCTTCGACAGACGGAAAAC
GGCCACGTCCATGATGCTGCGACTATCGCGGGTGCCCACGTCATAGAGCATCGGAACGAAAAAATCTGGTTGCTC
GTCGCCCTTGGGCGGCTTCCTAATCGACGGCGCACCGGCTGCCGGCGGTTGCCGGGATTCTTTGCGGATTCGATC
AGCGGCCGCTTGCCACGATTCACCGGGGCGTGCTTCTGCCTCGATGCGTTGCCGCTGGGCGGCCTGCGCGGCCTT
CAACTTCTCCACCAGGTCATCACCCAGCGCCGCGCCGATTTGTACCGGGCCGGATGGTTTGCGACCGTCACGCCG
ATTCCTCGGGCTTGGGGGTTCCAGTGCCATTGCAGGGCCGGCAGACAACCCAGCCGCTTACGCCTGGCCAACCGC
CCGTTCCTCCACACATGGGGCATTCCACGGCGTCGGTGCCTGGTTGTTCTTGATTTTCCATGCCGCCTCCTTTAG
CCGCTAAAATTCATCTACTCATTTATTCATTTGCTCATTTACTCTGGTAGCTGCGCGATGTATTCAGATAGCAGC
TCGGTAATGGTCTTGCCTTGGCGTACCGCGTACATCTTCAGCTTGGTGTGATCCTCCGCCGGCAACTGAAAGTTG
ACCCGCTTCATGGCTGGCGTGTCTGCCAGGCTGGCCAACGTTGCAGCCTTGCTGCTGCGTGCGCTCGGACGGCCG
GCACTTAGCGTGTTTGTGCTTTTGCTCATTTTCTCTTTACCTCATTAACTCAAATGAGTTTTGATTTAATTTCAG
CGGCCAGCGCCTGGACCTCGCGGGCAGCGTCGCCCTCGGGTTCTGATTCAAGAACGGTTGTGCCGGCGGCGGCAG
TGCCTGGGTAGCTCACGCGCTGCGTGATACGGGACTCAAGAATGGGCAGCTCGTACCCGGCCAGCGCCTCGGCAA

CCTCACCGCCGATGCGCGTGCCTTTGATCGCCCGCGACACGACAAAGGCCGCTTGTAGCCTTCCATCCGTGACCT
CAATGCGCTGCTTAACCAGCTCCACCAGGTCGGCGGTGGCCCATATGTCGTAAGGGCTTGGCTGCACCGGAATCA
GCACGAAGTCGGCTGCCTTGATCGCGGACACAGCCAAGTCCGCCGCCTGGGGCGCTCCGTCGATCACTACGAAGT
CGCGCCGGCCGATGGCCTTCACGTCGCGGTCAATCGTCGGGCGGTCGATGCCGACAACGGTTAGCGGTTGATCTT
CCCGCACGGCCGCCCAATCGCGGGCACTGCCCTGGGGATCGGAATCGACTAACAGAACATCGGCCCCGGCGAGTT
GCAGGGCGCGGGCTAGATGGGTTGCGATGGTCGTCTTGCCTGACCCGCCTTTCTGGTTAAGTACAGCGATAACCT
TCATGCGTTCCCCTTGCGTATTTGTTTATTTACTCATCGCATCATATACGCAGCGACCGCATGACGCAAGCTGTT
TTACTCAAATACACATCACCTTTTTAGACGGCGGCGCTCGGTTTCTTCAGCGGCCAAGCTGGCCGGCCAGGCCGC
CAGCTTGGCATCAGACAAACCGGCCAGGATTTCATGCAGCCGCACGGTTGAGACGTGCGCGGGCGGCTCGAACAC
GTACCCGGCCGCGATCATCTCCGCCTCGATCTCTTCGGTAATGAAAAACGGTTCGTCCTGGCCGTCCTGGTGCGG
TTTCATGCTTGTTCCTCTTGGCGTTCATTCTCGGCGGCCGCCAGGGCGTCGGCCTCGGTCAATGCGTCCTCACGG
AAGGCACCGCGCCGCCTGGCCTCGGTGGGCGTCACTTCCTCGCTGCGCTCAAGTGCGCGGTACAGGGTCGAGCGA
TGCACGCCAAGCAGTGCAGCCGCCTCTTTCACGGTGCGGCCTTCCTGGTCGATCAGCTCGCGGGCGTGCGCGATC
TGTGCCGGGGTGAGGGTAGGGCGGGGGCCAAACTTCACGCCTCGGGCCTTGGCGGCCTCGCGCCCGCTCCGGGTG
CGGTCGATGATTAGGGAACGCTCGAACTCGGCAATGCCGGCGAACACGGTCAACACCATGCGGCCGGCCGGCGTG
GTGGTGTCGGCCCACGGCTCTGCCAGGCTACGCAGGCCCGCGCCGGCCTCCTGGATGCGCTCGGCAATGTCCAGT
AGGTCGCGGGTGCTGCGGGCCAGGCGGTCTAGCCTGGTCACTGTCACAACGTCGCCAGGGCGTAGGTGGTCAAGC
ATCCTGGCCAGCTCCGGGCGGTCGCGCCTGGTGCCGGTGATCTTCTCGGAAAACAGCTTGGTGCAGCCGGCCGCG
TGCAGTTCGGCCCGTTGGTTGGTCAAGTCCTGGTCGTCGGTGCTGACGCGGGCATAGCCCAGCAGGCCAGCGGCG
GCGCTCTTGTTCATGGCGTAATGTCTCCGGTTCTAGTCGCAAGTATTCTACTTTATGCGACTAAAACACGCGACA
AGAAAACGCCAGGAAAAGGGCAGGGCGGCAGCCTGTCGCGTAACTTAGGACTTGTGCGACATGTCGTTTTCAGAA
GACGGCTGCACTGAACGTCAGAAGCCGACTGCACTATAGCAGCGGAGGGGTTGGATCAAAGTACTTTGATCCCGA
GGGGAACCCTGTGGTTGGCATGCACATACAAATGGACGAACGGATAAACCTTTTCACGCCCTTTTAAATATCCGT
TATTCTAATAAACGCTCTTTTCTCTTAGGtttacccgccaatatatcctgtcaAACACTGATAGTTTAAACTGAA
GGCGGGAAACGACAATCTGATCCAAGCTCAAGCTGCTCTAGCATTCGCCATTCAGGCTGCGCAACTGTTGGGAAG
GGCGATCGGTGCGGGCCTCTTCGCTATTACGCCAGCTGGCGAAAGGGGGATGTGCTGCAAGGCGATTAAGTTGGG
TAACGCCAGGGTTTTCCCAGTCACGACGTTGTAAAACGACGGCCAGTGCCAAGCTTGGCGTGCCTGCAGGTCAAC
ATGGTGGAGCACGACACACTTGTCTACTCCAAAAATATCAAAGATACAGTCTCAGAAGACCAAAGGGCAATTGAG
ACTTTTCAACAAAGGGTAATATCCGGAAACCTCCTCGGATTCCATTGCCCAGCTATCTGTCACTTTATTGTGAAG
ATAGTGGAAAAGGAAGGTGGCTCCTACAAATGCCATCATTGCGATAAAGGAAAGGCCATCGTTGAAGATGCCTCT
GCCGACAGTGGTCCCAAAGATGGACCCCCACCCACGAGGAGCATCGTGGAAAAAGAAGACGTTCCAACCACGTCT
TCAAAGCAAGTGGATTGATGTGATAACATGGTGGAGCACGACACACTTGTCTACTCCAAAAATATCAAAGATACA
GTCTCAGAAGACCAAAGGGCAATTGAGACTTTTCAACAAAGGGTAATATCCGGAAACCTCCTCGGATTCCATTGC
CCAGCTATCTGTCACTTTATTGTGAAGATAGTGGAAAAGGAAGGTGGCTCCTACAAATGCCATCATTGCGATAAA
GGAAAGGCCATCGTTGAAGATGCCTCTGCCGACAGTGGTCCCAAAGATGGACCCCCACCCACGAGGAGCATCGTG
GAAAAAGAAGACGTTCCAACCACGTCTTCAAAGCAAGTGGATTGATGTGATATCTCCACTGACGTAAGGGATGAC
GCACAATCCCACTATCCTTCGCAAGACCCTTCCTCTATATAAGGAAGTTCATTTCATTTGGAGAGGACCTCGACT
CTAGAGGATCCCCGGGTACCGGGCCCCCCCTCGAGGCGCGCCAAGCTATCAAACAAGTTTGTACAAAAAAGCAGG
CTCCGCGGCCGCCCCCTTCACCAGTAGAGAAGAATCTGT**G**AGAGACCATTAGGCACCCCAGGCTTTACACTTTAT
GCTTCCGGCTCGTATAATGTGTGGATTTTGAGTTAGGAGCCGTCGAGATTTTCAGGAGCTAAGGAAGCTAAAatg
gagaaaaaaatcactggatataccaccgttgatatatcccaatggcatcgtaaagaacattttgaggcatttcag
tcagttgctcaatgtacctataaccagaccgttcagctggatattacggcctttttaaagaccgtaaagaaaaat
aagcacaagttttatccggcctttattcacattcttgcccgcctgatgaatgctcatccggagttccgtatggca
atgaaagacggtgagctggtgatatgggatagtgttcacccttgttacaccgttttccatgagcaaactgaaacg
ttttcatcgctctggagtgaataccacgacgatttccggcagtttctacacatatattcgcaagatgtggcgtgt
tacggtgaaaacctggcctatttccctaaagggtttattgagaatatgtttttcgtctcagccaatccctgggtg
agtttcaccagttttgatttaaacgtggccaatatggacaacttcttcgcccccgttttcaccatgggcaaatat
tatacgcaaggcgacaaggtgctgatgccgctggcgattcaggttcatcatgccgtttgtgatggcttccatgtc
ggcagaatgcttaatgaattacaacagtactgcgatgagtggcagggcggggcgtaaACGCGTGGAGCCGGCTTA
CTAAAAGCCAGATAACAGTATGCGTATTTGCGCGCTGATTTTTGCGGTATAAGAATATATACTGATATGTATACC
CGAAGTATGTCAAAAAGAGGTATGCTATGAAGCAGCGTATTACAGTGACAGTTGACAGCGACAGCTATCAGTTGC
TCAAGGCATATATGATGTCAATATCTCCGGTCTGGTAAGCACAACCATGCAGAATGAAGCCCGTCGTCTGCGTGC
CGAACGCTGGAAAGCGGAAAATCAGGAAGGGATGGCTGAGGTCGCCCGGTTTATTGAAATGAACGGCTCTTTTGC
TGACGAGAACAGGGGCTGGTGAAATGCAGTTTAAGGTTTACACCTATAAAAGAGAGAGCCGTTATCGTCTGTTTG
TGGATGTACAGAGTGATATTATTGACACGCCCGGCCGACGGATGGTGATCCCCCTGGCCAGTGCACGTCTGCTGT
CAGATAAAGTCTCCCGTGAACTTTACCCGGTGGTGCATATCGGGGATGAAAGCTGGCGCATGATGACCACCGATA
TGGCCAGTGTGCCGGTTTCCGTTATCGGGGAAGAAGTGGCTGATCTCAGCCACCGCGAAAATGACATCAAAAACG
CCATTAACCTGATGTTCTGGGGAATATAAATGTCAGGCTCCCTTATACACAGCCAGTCTGCACCTCGACggtctc
AcattggctcttcttactAAGGGTGGGCGCGCCGACCCAGCTTTCTTGTACAAAGTGGTTCGATAATTCCTTAAT
TAACTAGTTCTAGAGCGGCCGCCCACCGCGGTGGAGCTCGAATTTCCCCGATCGTTCAAACATTTGGCAATAAAG
TTTCTTAAGATTGAATCCTGTTGCCGGTCTTGCGATGATTATCATATAATTTCTGTTGAATTACGTTAAGCATGT

AATAATTAACATGTAATGCATGACGTTATTTATGAGATGGGTTTTTATGATTAGAGTCCCGCAATTATACATTTA
ATACGCGATAGAAAACAAAATATAGCGCGCAAACTAGGATAAATTATCGCGCGCGGTGTCATCTATGTTACTGAA
TTCGTAATCATGGTCATAGCTGTTTCCTGTGTGAAATTGTTATCCGCTCACAATTCCACACAACATACGAGCCGG
AAGCATAAAGTGTAAAGCCTGGGGTGCCTAATGAGTGAGCTAACTCACATTAATTGCGTTGCGCTCACTGCCCGC
TTTCCAGTCGGGAAACCTGTCGTGCCAGCTGCATTAATGAATCGGCCAACGCGCGGGGAGAGGCGGTTTGCGTAT
TGGCTAGAGCAGCTTGCCAACATGGTGGAGCACGACACTCTCGTCTACTCCAAGAATATCAAAGATACAGTCTCA
GAAGACCAAAGGGCTATTGAGACTTTTCAACAAAGGGTAATATCGGGAAACCTCCTCGGATTCCATTGCCCAGCT
ATCTGTCACTTCATCAAAAGGACAGTAGAAAAGGAAGGTGGCACCTACAAATGCCATCATTGCGATAAAGGAAAG
GCTATCGTTCAAGATGCCTCTGCCGACAGTGGTCCCAAAGATGGACCCCCACCCACGAGGAGCATCGTGGAAAAA
GAAGACGTTCCAACCACGTCTTCAAAGCAAGTGGATTGATGTGATAACatggtggagcacgacactctcgtctac
tccaagaatatcaaagatacagtctcagaagaccaaagggctattgagacttttcaacaaagggtaatatcggga
aacctcctcggattccattgcccagctatctgtcacttcatcaaaaggacagtagaaaaggaaggtggcacctac
aaatgccatcattgcgataaaggaaaggctatcgttcaagatgcctctgccgacagtggtcccaaagatggaccc
ccacccacgaggagcatcgtggaaaaagaagacgttccaaccacgtcttcaaagcaagtggattgatgtgatatc
tccactgacgtaagggatgacgcacaatcccactatccttcgcaagaccttcctctatataaggaagttcatttc
atttggagaggACACGCTGAAATCACCAGTCTCTCTCTACAAATCTATCTCTCTCGAGCTTTCGCAGATCCCGGG
GGGCAATGAGATATGAAAAAGCCTGAACTCACCGCGACGTCTGTCGAGAAGTTTCTGATCGAAAAGTTCGACAGC
GTCTCCGACCTGATGCAGCTCTCGGAGGGCGAAGAATCTCGTGCTTTCAGCTTCGATGTAGGAGGGCGTGGATAT
GTCCTGCGGGTAAATAGCTGCGCCGATGGTTTCTACAAAGATCGTTATGTTTATCGGCACTTTGCATCGGCCGCG
CTCCCGATTCCGGAAGTGCTTGACATTGGGGAGTTTAGCGAGAGCCTGACCTATTGCATCTCCCGCCGTGCACAG
GGTGTCACGTTGCAAGACCTGCCTGAAACCGAACTGCCCGCTGTTCTACAACCGGTCGCGGAGGCTATGGATGCG
ATCGCTGCGGCCGATCTTAGCCAGACGAGCGGGTTCGGCCCATTCGGACCGCAAGGAATCGGTCAATACACTACA
TGGCGTGATTTCATATGCGCGATTGCTGATCCCCATGTGTATCACTGGCAAACTGTGATGGACGACACCGTCAGT
GCGTCCGTCGCGCAGGCTCTCGATGAGCTGATGCTTTGGGCCGAGGACTGCCCCGAAGTCCGGCACCTCGTGCAC
GCGGATTTCGGCTCCAACAATGTCCTGACGGACAATGGCCGCATAACAGCGGTCATTGACTGGAGCGAGGCGATG
TTCGGGGATTCCCAATACGAGGTCGCCAACATCTTCTTCTGGAGGCCGTGGTTGGCTTGTATGGAGCAGCAGACG
CGCTACTTCGAGCGGAGGCATCCGGAGCTTGCAGGATCGCCACGACTCCGGGCGTATATGCTCCGCATTGGTCTT
GACCAACTCTATCAGAGCTTGGTTGACGGCAATTTCGATGATGCAGCTTGGGCGCAGGGTCGATGCGACGCAATC
GTCCGATCCGGAGCCGGGACTGTCGGGCGTACACAAATCGCCCGCAGAAGCGCGGCCGTCTGGACCGATGGCTGT
GTAGAAGTACTCGCCGATAGTGGAAACCGACGCCCCAGCACTCGTCCGAGGGCAAAGAAATAGAGTAGATGCCGA
CCGGATCTGTCGATCGACAAGCTCGAGtttctccataataatgtgtgagtagttcccagataagggaattagggt
tcctatagggtttcgctcatgtgttgagcatataagaaacccttagtatgtatttgtatttgtaaaatacttcta
tcaataaaatttctaattcctaaaaccaaaatccagtactaaaatccagatcCCCCGAATTAATTCGGCGTTAAT
TCAGTACATTAAAAACGTCCGCAATGTGTTATTAAGTTGTCTAAGCGTCAATTTGTTTACACCACAATATATCCT
GCCA

brown/lowercase: kanamycin resistance gene
CYAN/UPPERCASE/UNDERLINED: C->A transversion to block vector's *Bsa*I site
cyan/lowercase: T-DNA right border
GREEN/UPPERCASE: 2x35S CaMV promoter
ORANGE/UPPERCASE: attB1
BLUE/UPPERCASE: *AtMIR390a* 5' region
RED/UPPERCASE/BOLD: A18G mutation
RED/UPPERCASE: *Bsa*I site
magenta/lowercase: chloramphenicol resistance gene
MAGENTA/UPPERCASE: *ccd*B gene
red/lowercase: inverted *Bsa*I site
blue/lowercase: *AtMIR390a* 3' region
ORANGE/UPPERCASE/UNDERLINED: attB2
GREY/UPPERCASE/UNDERLINED: Nos terminator
green/lowercase: CaMV promoter
BROWN/UPPERCASE: hygromycin resistance gene
green/lowercase/underlined: CaMV terminator
CYAN/UPPERCASE: T-DNA left border
